# Localized-Statistical Quantification of Human Serum Proteome Associated with Type 2 Diabetes

**DOI:** 10.1371/journal.pone.0003224

**Published:** 2008-09-16

**Authors:** Rong-Xia Li, Hai-Bing Chen, Kang Tu, Shi-Lin Zhao, Hu Zhou, Su-Jun Li, Jie Dai, Qing-Run Li, Song Nie, Yi-Xue Li, Wei-Ping Jia, Rong Zeng, Jia-Rui Wu

**Affiliations:** 1 Key Laboratory of Systems Biology, Institute of Biochemistry and Cell Biology, Shanghai Institutes for Biological Sciences, Chinese Academy of Sciences, Shanghai, China; 2 Shanghai Diabetes Institute, Department of Endocrinology & Metabolism, Shanghai No. 6 People's Hospital Affiliated to Jiaotong University, Shanghai, China; 3 Hefei National Laboratory for Physical Sciences at Microscale and School of Life Science, University of Science & Technology of China, Hefei, Anhui, China; University of Bremen, Germany

## Abstract

**Background:**

Recent advances in proteomics have shed light to discover serum proteins or peptides as biomarkers for tracking the progression of diabetes as well as understanding molecular mechanisms of the disease.

**Results:**

In this work, human serum of non-diabetic and diabetic cohorts was analyzed by proteomic approach. To analyze total 1377 high-confident serum-proteins, we developed a computing strategy called localized statistics of protein abundance distribution (LSPAD) to calculate a significant bias of a particular protein-abundance between these two cohorts. As a result, 68 proteins were found significantly over-represented in the diabetic serum (p<0.01). In addition, a pathway-associated analysis was developed to obtain the overall pathway bias associated with type 2 diabetes, from which the significant over-representation of complement system associated with type 2 diabetes was uncovered. Moreover, an up-stream activator of complement pathway, ficolin-3, was observed over-represented in the serum of type 2 diabetic patients, which was further validated with statistic significance (p = 0.012) with more clinical samples.

**Conclusions:**

The developed LSPAD approach is well fit for analyzing proteomic data derived from biological complex systems such as plasma proteome. With LSPAD, we disclosed the comprehensive distribution of the proteins associated with diabetes in different abundance levels and the involvement of ficolin-related complement activation in diabetes.

## Introduction

Diabetes mellitus (DM) is one of the most common metabolic disorders in the world, in which more than 90% are grouped to type 2 diabetes mellitus (T2DM) [1]. Given the predicted explosion in the number of T2DM cases worldwide [2], the biomedical researchers face much stronger challenges, particularly on understanding the pathogenesis of disease and discovering biomarkers for tracking the disease process.

T2DM is characterized by abnormal glucose homeostasis leading to hyperglycemia, and the serum glucose has been used as a golden standard for diabetes diagnosis. However, T2DM is a kind of disease involving defects of multiple organs, which cannot be distinguished through the measurement of the serum-glucose level. In addition, T2DM is a multiple-stage disease, which usually covers several decades from impaired plasma glucose to various complications. The serum-glucose level only reflects the consequence of multiple physiological disorders in the given stage. Therefore, many efforts have been made to identify genetic and protein markers to reveal the molecular/cellular details or progression of diabetes [3]–[9]. The genetic defects certainly render more probability to diabetes. On the other hand, the protein markers can track real-time status of diabetes. It has been found there are changes in the protein abundances of serum in diabetes progression [10], [11]. For instance, a number of studies suggest that the elevated circulating inflammatory biomolecules such as C-reactive protein and serum amyloid A can be used for predicting the development of T2DM [12]–[15]. However, since the traditional strategy of diabetic diagnosis only relies on the individual molecules as the biomarkers, the sensitivity and accuracy of the biomarkers might be fluctuated due to ethnic or personal variance [16]–[18]. Proteomic technology might provide the new solutions for solving this problem, which can identify large set of the proteins in cells or tissues through high-throughput methods, and provide a globe view of the protein changes associated with diabetes.

It is well known that serum severs the optimal resource for discovery of disease biomarkers. Up to now, a few proteomic analyses of serum related to diabetes have been reported. For example, Dayal B *et al.* used the protein-chip to identify the high-density lipoproteins apoA-I and apoA-II and their glycosylated products in patients with diabetes and cardiovascular disease [19]. Zhang *et al.* found that the protease inhibitors including clade A and C, alpha 2-macroglobulin, fibrinogen, and the proteins involved in the classical complement pathway such as complement C3, and C4 exhibited the higher expression-levels in insulin resistance/type-2 diabetes [20]. Bergsten *et al.* analyzed the serum proteins in T2DM by SELDI-TOF-MS and peptide-mass fingerprinting (PMF), and found the expression levels of apolipoprotein, complement C3 and transthyretin were over-represented, whereas albumin and transferrin were under-represented in T2DM [21].

However, none of these above works provided the real globe view for the protein profile of the diabetic serum, since the proteomic analysis of serum is a formidable challenge for its huge complexity and dynamic range [11], [22]. Recent advances in serum sample preparation such as a depletion of high abundance proteins can be coupled to 1D or 2D-LC-MS/MS analysis, which have provided the new ways for large-scale serum proteomic analysis [23]–[25]. However, the step of the depletion of the high abundance proteins might cause some artifacts. In the present study, we used a label-free proteomic method with LC-MS/MS to investigate the protein profiling between the non-diabetic and diabetic serum without removing the high abundant proteins. After analyzing the proteomics data according to the stringent criteria, a total of 3,010 proteins and 3,224 proteins were identified from the non-diabetic and diabetic serum, respectively. In-depth bioinformatic analysis was employed for these differential proteins between the non-diabetic and diabetic serum.

## Results

### Selection of non-diabetic subjects and diabetic patients

Previous studies observed that T2DM might occurred at a greater frequency in adults who are younger than 65 years, suggesting that people who are old than 65 without diabetes mellitus usually do not anticipate the genetic susceptibility [26]. Therefore, we set age criteria for sample cohort that an adult in the present study must be old than 65 years (Non-diabetic subjects: age 67.6±1.67 years; type 2 diabetic patients: age 67±1.71 years) in order to reduce the genetic effects related to T2DM between non-diabetic and diabetic cohort. Furthermore, the careful selection of samples was performed based on the clinical parameters of non-diabetic and diabetic cohorts. Supplementary Table S1 summarized the clinical parameters of the selected non-diabetic subjects and diabetic patients, in which type 2 diabetic patients group had higher FPG, PG2H, WT, BMI, HOMA, HbA1c and C-peptide compared with control. To reduce the individual variance of serum proteins within the cohort, we pooled all the serum of each cohort for proteomic analysis, respectively.

### Semi-quantitative proteomic identification in non-diabetic and diabetic serum

**Figure 1:**
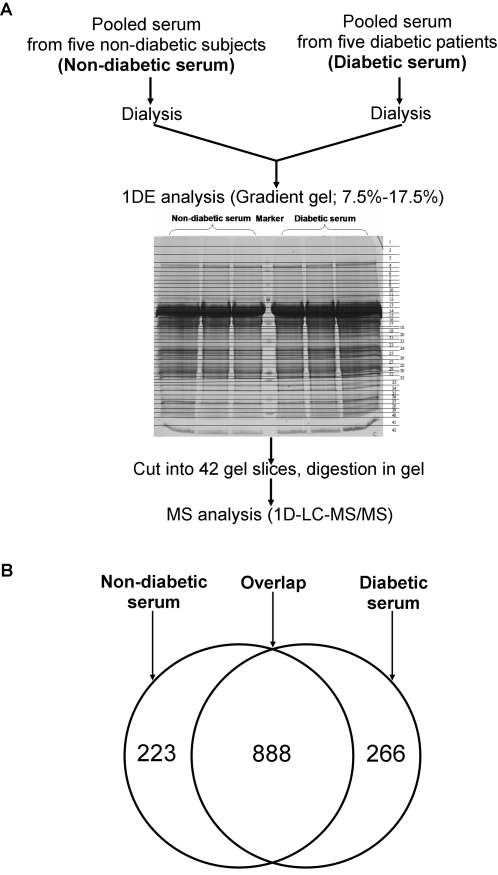
Overview of Idnetitication of proteins in non-diabetic and diabetic serum. (A) Scheme of label-free strategy to differential protein identification in non-diabetic and diabetic serum. Pooled serum samples from five non-diabetic and five diabetic sera were separated respectively by gel electrophoresis. Each gel lane was divided into 42 regions and each section was processed for mass spectrometry. (B) 1377 proteins were identified by at-least two peptide spectral counts in either serum. 888 overlapped proteins were identified both in non-diabetic and diabetic serum, whereas 223 proteins were identified uniquely from the non-diabetic serum and 266 proteins were found uniquely from the diabetic serum.

We analyzed differential protein profile in two cohorts using shotgun proteomics and label-free quantitative strategy. In order to reduce sample complexity, proteins in non-diabetic and diabetic serum were first separated on SDS-PAGE gel and the gel bands were excised and subjected to in-gel tryptic digestion, respectively (Figure 1A). The proteins were identified with criteria corresponding to an estimated false dicovery rate of 2.5%. After combining the MS/MS data generated from our experiment, we were able to assign 1,212,256 MS/MS spectra to 150,881 peptide counts, leading to identification of 5,882 unique peptides corresponding to 3,010 protein groups in non-diabetic serum, and 1,211,006 MS/MS spectra to 189,792 peptide counts, resulting in 5,960 unique peptides corresponding to 3,224 protein groups in diabetic serum (all these identified protein groups are called proteins in the text below for clarity). Supplementary Figure S1 showed the quite similar distributions of the identified peptides and proteins between non-diabetic and diabetic serum, indicating non-bias of the identified MS/MS spectra between non-diabetic and diabetic serum.

Among the identified 3,010 proteins in non-diabetic serum and 3,224 proteins in diabetic serum, 942 (30.30%) and 1,046 (32.44%) proteins were selected respectively under the condition that each identified protein contained at least two peptide spectral counts. Totally 1,377 proteins were obtained according to these more stringent filter, resulting the false discovery rate of 1.6%. There were 888 identified proteins overlapped in non-diabetic and diabetic serum, whereas 223 proteins were identified uniquely from the non-diabetic serum and 266 proteins were found uniquely from the diabetic serum (Figure 1B, Supplementary Table S2).

### Localized statistics of protein abundance distribution (LSPAD)

**Figure 2:**
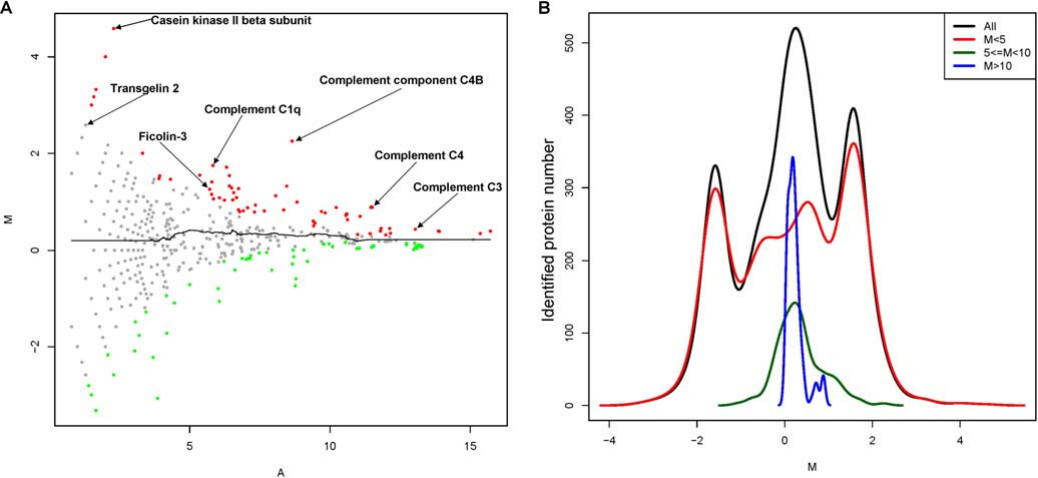
Quantitative strategy of proteins in non-diabetic and diabetic serum. (A) M-A plotting of 1377 identified proteins. “M” was defined as differential protein abundance ratios of each protein between diabetic and non-diabetic serum, and “A” was defined as protein-abundance of each protein. In addition, ret dots represented statistically significant over-represented proteins in diabetic serum, green dots represented statistically significant under-represented proteins in diabetic serum, and grey dots were proteins without statistically-significant change in diabetic serum and non-diabetic serum. (B) The distribution profiles of 1377 identified proteins (black line), identified proteins with M less than 5 (red line), between 5 and 10 (green line), and more than 10 (blue line).

Since the peptide-spectral-count distributions of identified 1377 serum-proteins were widely spread out to the range of 10^5^ (Supplementary Table S2), we developed M-A plotting referring to microarray analysis in order to display a relative protein-abundance distribution of each protein. First, for each protein, X_1_ representing its peptide spectral counts in diabetic serum was transformed into Y_1_ with formula *f*(*X_1_*) = log_2_(*X_1_*+1) as diabetic protein abundance, while the X_2_ in non-diabetic serum was transformed into Y_2_ with the same formula as a non-diabetic protein abundance. Then, we defined “M” as differential protein abundance between diabetic and non-diabetic serum by the formula of Y_1_−Y_2_, and “A” as an average protein abundance by the formula of (Y_1_+Y_2_)/2. Based on these formulas, total 1377 proteins were plotted as a scatter chart, in which the values of M were distributed on the Y-axis, and the values of A were distributed on the X-axis (Figure 2A).

This scatter chart showed that the log2-ratio-range of the differential protein-abundances between non-diabetic and diabetic serum was considerably decreased along M-axis when the protein-abundances were increased along A-axis (Figure 2A). These observations indicated that the abundance ratio based on peptide spectral counts cannot be simply used as indicators for differential significance between diabetic and non-diabetic serum. For example, the significance of 2-fold change from 2 to 1 peptide spectral counts is not equal to the significance of 2-fold change from 20000 to 10000. In addition, we realized that the protein-distribution profiles at the low, middle and high level of protein abundance, respectively, were considerably different (Figure 2B), suggesting significance-calculation of particular differential proteins should be localized to a certain range of related abundance level. Therefore, we developed a computing method called Localized Statistics of Protein Abundance Distribution (LSPAD) to evaluate the statistical significance of protein-abundance bias between diabetic and non-diabetic serum, by which the differentia significance of a particular protein should be calculated through its local protein-abundance distribution-window rather than through whole distribution range from the lowest to highest protein-abundances. Since the whole distribution range of protein abundances could be generally subdivided into three parts (high, middle and low protein-abundances, see Figure 2 and Supplementary Table S2), we postulated a width of the local window for statistics as 33%, i.e. only neighbored proteins with A value located within the 33% A-axis around a particular protein should be used for calculation.

In detail, for a particular protein, all the average peptide spectral counts of neighbored proteins whose A value were within the 33% abundance-window of the target protein were calculated as a background to evaluate the statistical significance (p value) of over-representation or under-representation of the target protein by performing fisher's exact test on a following four-fold table:

**Table:** 

	D	ND
Peptide spectral counts of a target protein	X_1_	X_2_
Sum of counts of all the other proteins in the window	S_1_	S_2_

The p-values derived from the fisher's exact test were linearly transformed into p′ in order to evaluate the bias of each protein-abundance between diabetic and non-diabetic serum. 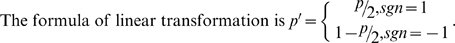
(sgn = 1 indicates that a protein is over-represented in diabetic sample, and sgn = −1 indicates that a protein is over-represented in non-diabetic sample)

To evaluate the reliability of LSPAD, we carried out the MA-plotting analyses to two duplicates of diabetic serum sample. First, the duplicates of one pooled diabetic-serum sample were separated by SDS-PAGE, and the entire gel was cut into 12 gel slices for LC-MS/MS analysis (Supplementary Figure S2A). The results showed the consistent proteomic data from these two duplicates (Supplementary Figure S2B–E). Then these data were subjected to LSPAD analysis. The result showed few protein-variants by comparing the protein-abundances between two duplicates of one pooled diabetic-serum sample with LSPAD method (Supplementary Figure S3A). Furthermore, we analyzed the expression-differentiation significance of one diabetic-serum duplicate versus a non-diabetic serum (Supplementary Figure S3B), and the other diabetic-serum duplicate versus the same non-diabetic serum (Supplementary Figure S3C). The Supplementary Figure S3D showed the high correlation coefficient of the M values between the significantly differential proteins in Supplementary Figure S3B and S3C. Taken together, these results indicate that this LSPAD method is reliable for exploring the differentiation of the protein abundances between non-disease and disease serum.

Accordingly, after 42 gel bands were analyzed in diabetic and non-diabetic serum respectively (Figure 1), 1377 identified proteins were analyzed by LSPAD approach. All the significant abundance-biases of 1377 proteins were calculated (Supplementary Table S2). Furthermore, we marked the proteins with p′<0.01 in red color as the significantly over-represented in diabetic serum, the proteins with p′>0.99 in green color as the significantly under-represented in diabetic serum, and the non-significantly differential proteins in grey color (Figure 2).

The 68 significant over-represented proteins in diabetic serum were listed in Table 1. Many known risk factors of diabetes such as C-reactive protein, serum amyloid A and haptoglobin were over-represented in diabetic serum, in agreement with the observations by traditional approaches based on the analysis of individual proteins [27]. In addition, a number of other factors including the novel proteins associated with diabetes were detected by this large-scale survey (Table 1). On the other hand, 74 proteins were found under-represented in diabetic serum (Supplementary Table S2). As far as we know, some studies reported that Keratin and IgG were associated with diabetes [28], [29]. In addition, a lot of keratins were also involved in the pathway of cell communication (Supplementary Figure S4) in our results. According to our pathway-associated differential significance analysis, we found keratin associated pathway were significantly overall bias with diabetic serum, which might not result from the bias of sample preparation.

**Table 1:** Characterization of proteins significantly over-represented in diabetic serum compared to non-diabetic serum based on LSPAD method. (P<0.01).

IPI ID	Protein name	Diabetic peptide spectral count	Non-diabetic peptide spectral count	P value
IPI00022434	ALB protein	61457	47082	4.09E-91
IPI00514824	Complement component C4B	875	183	1.44E-80
IPI00555805	Complement component 4A	3896	2109	1.63E-69
IPI00032258	Complement C4 precursor	3846	2077	3.06E-69
IPI00453459	Complement Component 4B preproprotein	3933	2141	9.77E-69
IPI00418163	C4B1	3811	2077	2.48E-66
IPI00384697	ALB protein	47105	37323	6.64E-37
IPI00556148	Complement factor H	2732	1691	1.30E-30
IPI00465313	Alpha 2 macroglobulin variant	17016	13013	7.50E-26
IPI00478003	Alpha-2-macroglobulin precursor	17344	13335	3.06E-24
IPI00385264	Ig mu heavy chain disease protein	1614	880	4.42E-23
IPI00164623	Complement C3 precursor	9754	7267	8.64E-22
IPI00479708	Immunoglobulin heavy constant mu (IGHM)	2007	1204	1.02E-21
IPI00549273	Immunoglobulin heavy constant mu (IGHM)	1995	1190	3.09E-21
IPI00019943	Afamin precursor	553	221	1.57E-20
IPI00479169	65 kDa protein	1932	1181	2.35E-18
IPI00022488	Hemopexin precursor	1952	1268	2.99E-14
IPI00426051	Hypothetical protein DKFZp686C15213	5203	3835	6.18E-14
IPI00021727	C4b-binding protein alpha chain precursor	638	321	1.01E-13
IPI00478493	Haptoglobin precursor	4214	3100	7.28E-12
IPI00550991	Alpha-1-antichymotrypsin precursor	1088	628	2.99E-11
IPI00019591	Splice Isoform 1 of Complement factor B precursor	1183	696	4.42E-11
IPI00021842	Apolipoprotein E precursor	394	181	3.28E-10
IPI00019399	Serum amyloid A-4 protein precursor	143	43	9.21E-10
IPI00021857	Apolipoprotein C-III precursor	144	49	3.87E-08
IPI00022392	Complement C1q subcomponent, A chain precursor	103	30	1.25E-07
IPI00021841	Apolipoprotein A-I precursor	4069	3112	2.14E-07
IPI00010865	Casein kinase II beta subunit	23	0	2.70E-07
IPI00396929	PREDICTED: similar to immunoglobulin M chain	165	68	1.55E-06
IPI00410714	Alpha 2 globin variant	433	244	3.33E-06
IPI00163446	The Human Immunoglobulin Heavy Diversity (IGHD)	134	53	4.03E-06
IPI00171834	Keratin, type I cytoskeletal 19	140	57	1.29E-05
IPI00399007	Hypothetical protein DKFZp686I04196	5114	4039	1.41E-05
IPI00003590	Quiescin Q6	15	0	4.53E-05
IPI00022389	Splice Isoform 1 of C-reactive protein precursor	15	0	4.53E-05
IPI00015309	Keratin, type I cytoskeletal 12	89	33	7.63E-05
IPI00290077	Keratin, type I cytoskeletal 15	142	62	8.21E-05
IPI00217963	Keratin, type I cytoskeletal 16	223	117	0.000146102
IPI00418422	The Human Immunoglobulin Heavy Diversity (IGHD)	69	23	0.000152193
IPI00423461	Hypothetical protein DKFZp686C02220	828	548	0.000223242
IPI00450768	Keratin, type I cytoskeletal 17	147	69	0.000275352
IPI00011261	Complement component C8 gamma chain precursor	266	152	0.000391696
IPI00556567	Ficolin-3 protein	80	33	0.000819734
IPI00441196	Hypothetical protein	3090	2450	0.000949718
IPI00386839	Amyloid lambda 6 light chain variable region SAR	180	98	0.001229635
IPI00017601	Ceruloplasmin precursor	2260	1816	0.001476612
IPI00383953	VH4 heavy chain variable region precursor	132	64	0.001483067
IPI00009866	Keratin, type I cytoskeletal 13	107	52	0.001918932
IPI00470798	Hypothetical protein DKFZp686E23209	4508	3647	0.002098573
IPI00017530	Ficolin-2 precursor	9	0	0.002266054
IPI00021854	Apolipoprotein A-II precursor	853	582	0.002359293
IPI00004550	Hypothetical protein FLJ20261	96	45	0.00238822
IPI00011252	Complement component C8 alpha chain precursor	81	36	0.002415224
IPI00293898	Hepatocellular carcinoma associated protein TB6	19	4	0.002727717
IPI00384444	Keratin, type I cytoskeletal 14	207	120	0.003122976
IPI00021856	Apolipoprotein C-II precursor	32	11	0.00418406
IPI00219806	S100 calcium-binding protein A7	8	0	0.004391148
IPI00446354	Hypothetical protein FLJ41805	8	0	0.004391148
IPI00479762	115 kDa protein	8	0	0.004391148
IPI00022446	Platelet factor 4 precursor	82	39	0.00501026
IPI00300725	Keratin, type II cytoskeletal 6A	158	90	0.005161139
IPI00242956	Fc fragment of IgG binding protein	24	8	0.006549075
IPI00384401	Myosin-reactive immunoglobulin kappa chain variable region	25	8	0.006595492
IPI00293665	Keratin, type II cytoskeletal 6B	141	79	0.00706398
IPI00299145	Keratin, type II cytoskeletal 6E	144	83	0.007903541
IPI00383603	Anti-thyroglobulin light chain variable region	7	0	0.008537501
IPI00452748	Serum amyloid A protein precursor	7	0	0.008537501
IPI00021304	Keratin, type II cytoskeletal 2 epidermal	810	575	0.009876282

### Pathway-associated differential significance analysis

To further reveal the significant bias of the protein abundances at the level of biological pathways in diabetic serum, we mapped those 1377 proteins into KEGG pathways [30]. Total 1377 identified proteins in the present study involved in 147 related pathways (Supplementary Table S3). Then, we calculated these proteins with their p-values at the pathway level in order to discover overall bias of pathways associated with diabetic-serum. The calculation procedure was as follows: Supposing all the proteins are non-differential expressed and independent of each other, their p-values, *p*, should follow a uniform distribution between[*0*,*1*]. Thus, *z* = *qnorm*(*p*), should follow a standard normal distribution (here qnorm is normal inverse distribution function). After the normal inverse transformation of *p_i_* to *z_i_*, the summarized Z score for a certain pathway *j* was generated by the formula, 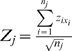. Here *n_j_* was the number of the proteins involved in the pathway *j* in our experiments, and *ix* = {*ix_i_*} denoted the index of these proteins. Because the proteins involved in the pathway *j* were supposed to be non-differential expressed and independent of each other, the summarized score for pathway *j*, *Z_j_*, should also follow a standard normal distribution. In our case, for pathway *j*, the following hypothesis test was performed:

H0: *Z_j_* follows the standard normal distribution, indicating that the pathway is not un-biased in diabetic serum.
H1: *Z_j_* doesn't follow the standard normal distribution, indicating that the pathway is over-represented or under-represented in diabetic serum

P value for pathway *j*, *P_j_*, was transformed from *Z_j_* by a normal cumulative function, *p* = *pnorm*(*z*). Under a statistic significance threshold *α*, an over-represented pathway in diabetic serum was identified with 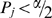 and under-represented pathway was identified with 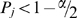. If the P value is less than 0.01, it indicates that this pathway is significantly overall overrepresented in diabetic serum compared with non-diabetic serum. If the P value is more than 0.99, it means that this pathway is significantly overall overrepresented in non-diabetic serum.

**Figure 3:**
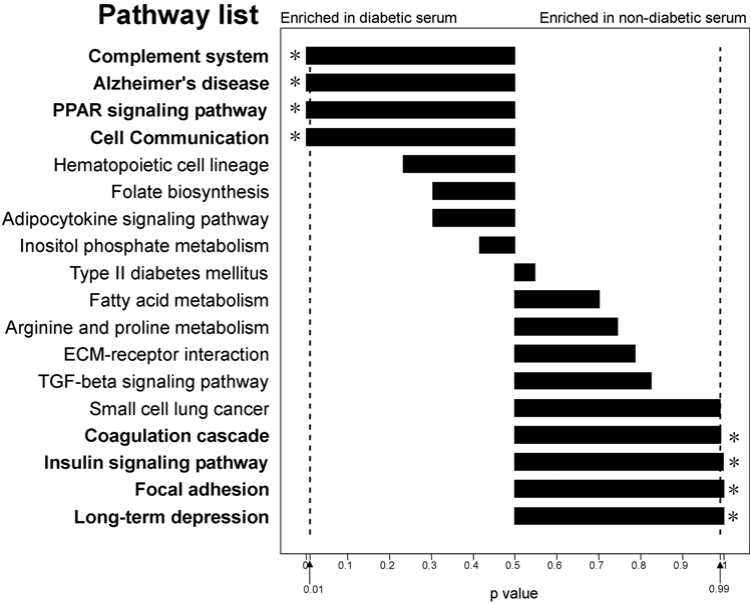
The overall bias analysis of selected pathways found in non-diabetic and diabetic serum. Proteins identified in non-diabetic and diabetic serum were mapped to known pathways using KEGG. The p value of each pathway was digitized to the length of the bar diagram.

Among the 147 pathways, we selected 18 pathways, in which each pathway should have at least 5 identified proteins as well as more than 10% coverage of all the pathway-proteins in the database, to evaluate the pathway-bias between non-diabetic and diabetic serum. All the values of the protein-abundance biases in these 18 pathways were presented in Supplementary Figure S4. Particularly, the pathways of complement system, PPAR system, cell communication and Alzheimer's disease showed the significantly overall over-representation in diabetes serum (p<0.01), while insulin signaling, coagulation cascade, focal adhesion and long-term pathways presented significantly overall bias in non-diabetic serum (p>0.99) (Figure 3).

**Figure 4:**
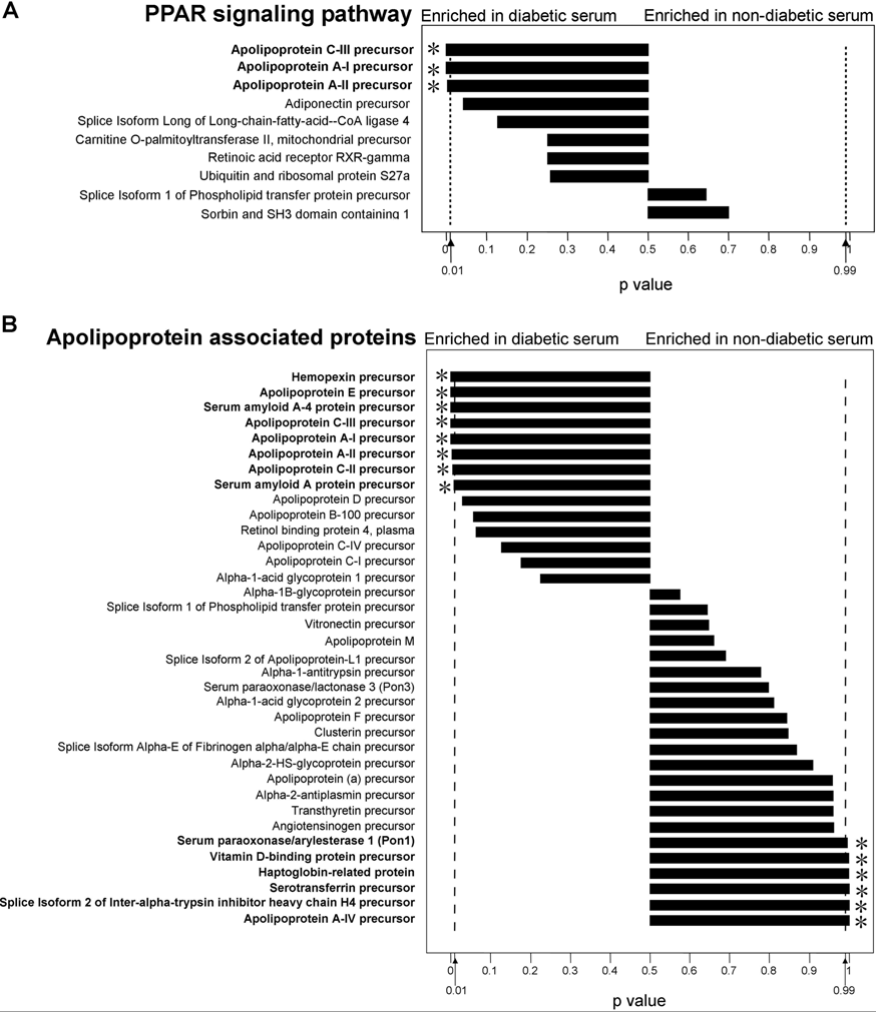
The identified proteins and abundance biases in specific pathways. (A)PPAR system, (B) Apolipoproteins associated Lipid metabolism. The p value of identified protein was digitized to the length of the bar in each pathway.

These significant differential pathways could be subdivided into two major categories: one had many significant-differential components in one pathway, and the other had a few highly significant-differential components in one pathway. For example, on the PPAR pathway, three apolipoproteins were all over-represented significantly in diabetic serum (Figure 4A). In Alzheimer's disease pathway, the apoliprotein E over-presentation also contributed the overall bias of this pathway to diabetic serum. Therefore, apolipoproteins could be considered as a kind of the important biomarkers associated with diabetes. As previous reports, many apolipoproteins were involved in lipid metabolism [31]–[43]. These proteins were further selected to show their abundance biases between non-diabetic and diabetic serum. As shown in Figure 4B, 8 proteins including apolipoprotein A-I, AII, C-II and C-III were significantly over-represented in diabetic serum, whereas 6 proteins were significantly under-represented in diabetic serum, which covered some regulatory factors such as paraoxonase 1 (PON1) in lipid metabolism.

### Over-representation of ficolin-related complement pathway in diabetic serum

**Figure 5:**
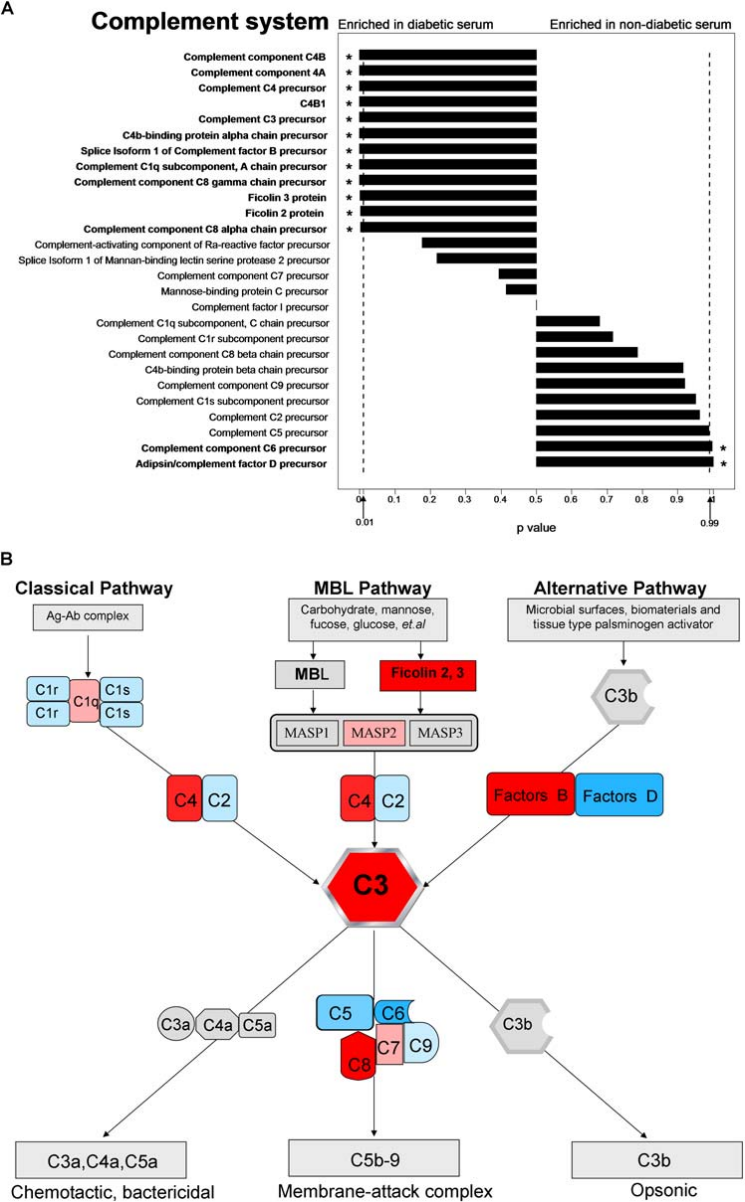
Overview of proteins associated with complement system. (A) The identified proteins and the abundance biases in complement system. The p value of identified protein was digitized to the length of the bar in each pathway. (B) The three activation pathways of complement system: the classical, mannose-binding lectin, and alternative pathways. The three pathways converge at the point of cleavage of C3. Therefore, the C3 cleavage is the crucial step in activation of the three complement pathway. Molecules of C3 are cleaved to C3a and C3b by the C3 convertase. C3b binds covalently around the site of complement activation. Some of this C3b binds to the C4b and C3b in the convertase enzymes of the classical and alternative pathways, respectively, forming C5 convertase enzymes. This C3b acts as an acceptor site for C5, which is cleaved to form the anaphylatoxin C5a and C5b, which initiates the formation of the membrane-attack complex. Excitedly, ficolin-3 is a biologically active protein of the lectin-complement activation in association with MBL-associated serine protease (MASP). In this figure, significantly up-regulated proteins in diabetic serum were denoted with red color, slightly up-regulated proteins in diabetic serum were denoted with light red color, significantly up-regulated proteins in non-diabetic serum were denoted with blue color, and slightly up-regulated proteins in non-diabetic serum were denoted with light blue color. Not identified proteins or the fragment of the complement component were denoted with light grey color.

Our results showed that 12 proteins associated with complement system were significantly over-represented in diabetic serum (Figure 5A). It has been known that the complement system can be activated through three different ways, including lectin, classical and alternative pathways (Figure 5B) [44], [45]. The present work showed that two trigger factors of lectin-complement activation, ficolin-2 and ficolin-3, were both over-represented significantly in the diabetic serum (Table 1), while the ficolin-3 was detected with much higher abundance than ficolin-2. Another kind of lectin related to complement initiation, mannose biding lectins (MBL), was not detected. These results indicate that ficolin-3 might be the major trigger of lectin-complement activation in diabetic patients.

### Validation of ficolin-3 related complement activation in diabetic serum

**Figure 6:**
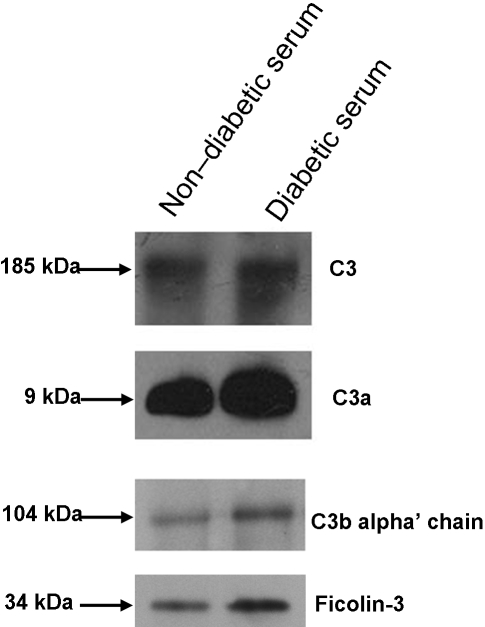
Western blot confirmation of the serum level of C3 (∼187 kD), C3a (∼9 kD), C3b (alpha' chain, ∼104 kD) and Ficolin-3 (∼34 kD). The Non-diabetic serum: the mixture of equal amount of serum from five non-diabetic subjects in Table 1, Diabetic serum: the mixture of equal amount of serum from five diabetic patients in Table 1.

When the complement system is activated, the complement C3 is cleaved to C3a and C3b, which is the common and crucial step in all complement activation pathways (as shown in Figure 5B, [46]). To validate the level of C3 and its activation, Western blotting for C3, corresponding fragment C3a and C3b were performed in the non-diabetic and diabetic serum. It was confirmed that these proteins were over-represented in diabetic serum (Figure 6).

**Figure 7:**
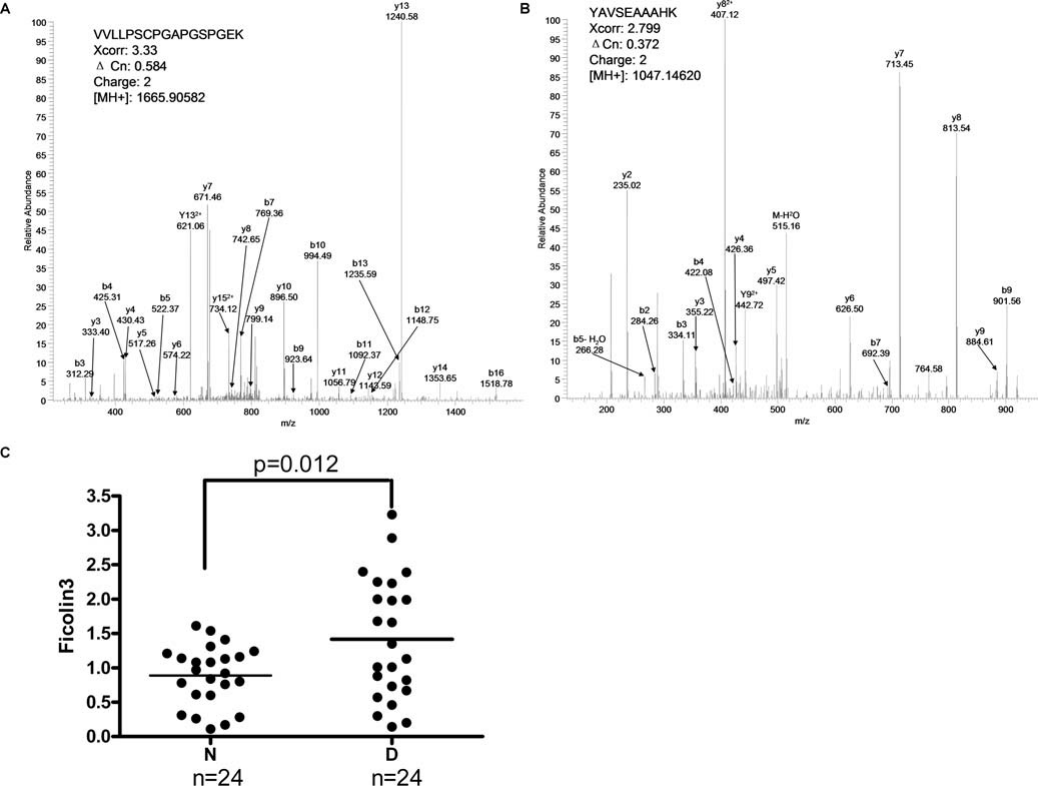
MS/MS spectra of representative peptides from ficolin-3 and validation of ficolin-3 up-regulation in diabetic sera. (A) VVLLPSCPGAPGSPGEK (B) YAVSEAAAHK (C) Western blot validation of the serum ficolin-3 level in the non-diabetic subjects and diabetic patients (n = 24, respectively) were conducted. N: non-diabetic serum; D: diabetic serum.

It has been known that lectin is one of the trigger to complement activation [46], [47]. Our studies identified 33 and 80 spectral peptide counts of ficolin-3 from non-diabetic and diabetic serum, respectively (Table 1). Among these detected peptides, two particular peptides (VVLLPSCPGAPGSPGEK and YAVSEAAAHK) were detected exclusively from diabetic serum (Figure 7A and 7B). Taken together, these findings indicate that ficolin-3 in diabetic serum are over-represented in diabetic serum. We further confirmed this observation by Western blotting (Figure 6).

In order to evaluate the correlation of ficolin-3 with diabetes, the protein-abundance of ficolin-3 was validated by Western blotting in additional clinical sera from 24 non-diabetic subjects and 24 diabetic patients (Supplementary Table S4). As shown in Figure 7C and Supplementary Figure S5, the level of serum ficolin-3 was 0.90±0.43 in non-diabetic sera and 1.43±0.87 in diabetic sera (p = 0.012). Taken together, these results suggest a ficolin-3 related complement activation in diabetic serum.

## Discussion

### The strategy for analyzing the highly dynamical range of protein abundances

In this study, LC-MS/MS coupled with a label-free quantitative strategy was applied to analyze the differential serum-protein abundance profile between non-diabetic and diabetic patients. The label-free quantitation based on peptide-spectral counts offers a high-coverage identification of proteins, and then gives a comprehensive and rapid comparison to the differential proteins, especially to the plasma proteins [48]. Since the distribution range of the peptide-spectral counts of the serum-proteins was up to 10^5^ (Supplementary Table S2), we applied M-A plotting method referring to microarray data-analysis for analyzing the effects of the different abundance-levels as well as the count-ratio of a particular protein between non-diabetic and diabetic serum (Figure 2A). From the Figure 2B, we realized that the lower the abundance-level of the peptide-spectral counts, the higher the deviation of the count-ratio. In this regard, we cannot fix a count-ratio as a threshold covering low abundance-level to high abundance-level for evaluating the bias of the protein abundance of diabetic serum. In other words, the quantitative selection of differentia proteins based on the ratio of the particular protein-abundance, which is usually used in isotope-labeling proteomic methods, seems not suitable in the peptide-spectral counts quantification for the systems with the highly dynamic range of protein-abundances, i.e. serum proteome.

Therefore, we developed a localized statistics of protein abundance distribution (LSPAD) for identifying the over- or under-represented proteins in diabetic serum. Based on this method, we can calculate the significance of the peptide-spectral-count bias for differentia proteins instead of using the count-ratio. Furthermore, we defined an abundance-window of 33% around a target protein as a localized background for calculating the statistical significance, by which we can evaluate the significant bias of a target protein-abundance compared to the abundance-distribution range of its neighbored proteins rather than to the abundance-distribution range of all identified proteins.

### Involvement of lipid metabolism and inflammation in type 2 diabetes

In this study, many individual proteins associated with T2DM reported in previous studies were also identified. In the group of apolipoproteins, for example, many components were over-represented in diabetic serum including Apolipoprotein E, CII, CIII and serum amyloid. Apo E content of postprandial TG-rich lipoproteins in subjects with both T2DM and coronary artery disease was increased [49]. Serum amyloid A, a major apoprotein (45%) in high-density lipoproteins (HDL), was increased due to inflammation [50]. Apolipoprotein C III (apo C III) plays a central role in regulating plasma metabolism of triglyceride-rich lipoprotein (TRL). Previous studies suggested that apo C III might be an independent risk factor for atherosclerotic diseases in Chinese type 2 diabetes [51]. On the other hand, we identified some under-represented regulatory factors in lipid metabolism such as paraoxonase1 (PON1). PON1 is an anti-inflammatory enzyme, which participates in the prevention of low density lipoprotein (LDL) oxidation [52], [53]. Recently, Mackness *et. al* reported that high C-reactive protein and low paraoxonase1 in diabetes might be used as risk factors of coronary heart disease [53].

We also found certain proteins associated with acute-phase response were over-represented in diabetic serum such as C-reactive protein [54], [55], serum amyloid A [56], haptoglobin [57], α-1-acid glycoprotein [12], ceruloplasmin [58] and Von Willebrand factor [59]. Recently, abundant scientific evidences suggested the elevated circulating inflammatory markers such as C-reactive protein could be used for the prediction of the development of T2DM [12]–[15]. Moreover, C- reactive protein was also as a biomarker for inflammation in uremia [60]. Studies also showed that haptoglobin and C-reactive protein were increased significantly in both diabetes and glucose intolerance [57]. There has been an explosion of interests that the chronic low-grade inflammation and the activation of the innate immune system were closely involved in the pathogenesis of T2DM [61].

### Complement activation in type2 diabetes

Cross-sectional study have demonstrated strong correlation between complement C3 and insulin resistance, which showed that C3 was associated with a increased risk of developing diabetes [47]. In the present study, the serum levels of C3 and its fragments C3a were over-represented in diabetic serum by western blot analysis, indicating the activation of complement system. Adipsin/complement factor D is a serine protease that is secreted by adipocytes into the bloodstream. Adipsin is deficient in several animal models of obesity [62]. In our study, the expressing level of adipsin was under-represented in diabetic serum. Lectin is also a trigger for complement activation. This process begins due to the binding of mannose-binding lectin (MBL) or ficolins with MBL-associated serine protease-2 (MASP-2), and leads to the formation of a C3 convertase [63]–[66]. Up to now, only a few evidences showed that the increased level of MBL can provide prognostic information in patients with T2DM [67]. In the present work, MBL was not detected by mass spectrometry in serum, while both ficolin-2 and ficolin-3 were detected over-represented in diabetic serum. However, ficolin-2 was uniquely identified in diabetic serum with only 9 spectral counts while ficolin-3 was detected with much higher spectral counts. Therefore, it seems that ficolin-3 should be the major trigger and indicator of lectin-complement activation. The Western-blotting of serum ficolin-3 with a lager clinical population supports that serum ficolin-3 was significantly over-represented and positively correlated with T2DM. Thus, we argue that ficolin-3 triggers the lectin-complement pathway, which might play an important role in the chronic low-grade inflammation and activation of the innate immune system associated with T2DM.

In summary, the LSPAD approach developed in this present work is well useful for analyzing proteomic data derived from biological complex systems such as plasma proteome, by which we disclosed the comprehensive distribution of the proteins associated with diabetes among high, medium and low abundant proteins. In addition, we found the involvement of the ficolin-related complement system in type 2 diabetes.

## Materials and Methods

### Clinical sample collection and preparation

Ten male adults were selected for this investigation, including five non-diabetic subjects (FPG 4.82±0.21 mmol/L; PG2H 4.78±1.54 mmol/L; BMI 21.67±0.81 kg/m2; HbA1c 5.68±0.54%; C-peptide 1.09±0.25 ng/mL; and homeostasis model assessment [HOMA] 1.04±0.67), and five type 2 diabetic patients (FPG 7.26±2.73 mmol/L; PG2H 12.2±1.21 mmol/L; BMI 27.03±4.23 kg/m2; HbA1c 7.14±0.42%; C-peptide 3.44±1.31 ng/mL; HOMA 5.67±3.96). The Homeostasis Model Assessment (HOMA) for insulin resistance and β-cell function was calculated from fasting plasma glucose and insulin concentrations. Informed consent was obtained from each person in written format and approved by Shanghai No. 6 People's Hospital Review Committee.

Immediately after collection, fasting blood samples were allowed to clot at room temperature for four hours, and the serum were collected and centrifugated at 3000 rpm/min for 15 min. Before pooling the samples, the protein concentration of the serum samples was determined by Bradford assay on a Microplate Reader (Bio-Rad, Model 680). Five non-diabetic serum samples were mixed as control-pool sample, and five diabetic serum samples were also mixed as disease-pool sample. The two pooled serum samples were diluted respectively to ∼20 mg/mL with 100 mM phosphate buffer (pH 2.0, containing 5% ACN). Then, the pooled serum samples were filtered through 0.22 µm filters (Agilent technologies) by spinning at 10 000 g at 4°C for 30 min and dialyzed to 100 mM phosphate buffer (pH 2.0, containing 5% ACN).

### Gel electrophoresis and In-Gel Digestion

The serum sample containing 1.8 mg proteins was reduced by adding 2 µL of 1 M DTT to 10 mM and incubated at 37°C for 2.5 hours. The mixture then was added with 10 µL of 1 M IAA and incubated for 40 min in darkness at room temperature. After these treatments, the samples were subjected to SDS-PAGE on a 7.5–17.5% gradient gel. The gel lane stained with Coomassie Blue was excised into 42 sections. Each excised section was cut into approx. 1 mm^3^ pieces and destained using 30% acetonitrile/70% 100 mM ammonium bicarbonate solution, followed by dehydration in 100% acetonitrile for 5 min. Gel pieces were placed under vacuum centrifugation until completely dried. Each gel slice was then incubated in a 50 mM ammonium bicarbonate solution containing 10 ng/µL trypsin (Promega Biotech Co., Madison, WI, USA.) overnight. Peptides were extracted with 0.1% TFA/80% acetonitrile, dried by vacuum centrifugation, and stored at −80°C for further analysis with mass spectrometry.

### Label-free shotgun proteomic identification

Each gel slice containing peptides was dissolved in 60 µL 0.1% formic acid, and then the half of this peptide-solution was loaded into the RP column. RP-HPLC was performed using an Agilent 1100 Capillary system (Agilent technologies) with C18 column (150 µm i.d., 100 mm length, Column technology Inc., Fremont, CA). The pump flow rate was 1.6 µL/min. Mobile phase A was 0.1% formic acid in water, and mobile phase B was 0.1% formic acid in acetonitrile. The tryptic peptide mixtures were eluted using a gradient of 2–55% B over 135 min. The mass spectral data were acquired on a LTQ linear ion trap mass spectrometer (Thermo, San Jose, CA) equipped with an electrospray interface operated in positive ion mode. The temperature of heated capillary was set at 170°C. A voltage of 3.0 kV applied to the ESI needle. Normalized collision energy was 35.0. The number of ions stored in the ion trap was regulated by the automatic gain control. Voltages across the capillary and the quadrupole lenses were tuned by an automated procedure to maximize the signal for the ion of interest. The mass spectrometer was set as one full MS scan was followed by ten MS/MS scans on the ten most intense ions from the MS spectrum with the following Dynamic Exclusion™ settings: repeat count, 2, repeat duration, 0.5 min, exclusion duration, 1.5 min.

### Data analysis

All .dta files were created using Bioworks 3.1, with precursor mass tolerance of 1.4 Da, threshold of 100, and minimum ion count of 15. The acquired MS/MS spectra were searched against the Human International Protein Index protein sequence database (version 3.07, www.ebi.ac.uk/IPI) combined with sequences of real protein and reverse sequences of proteins, by using the TurboSEQUEST program in the BioWorks 3.1 software suite, with a mass tolerance of 3.0 Da. All cysteine residues were searched as carboxamidomethycystein (+57.02 Da). Up to one internal cleavage sites were allowed for tryptic searches. All output results were combined together using the in-house software named BuildSummary to delete the redundant data. Searches were conducted against the Human International Protein Index protein sequence database to control the false discovery rate at 2.5% and all spectral peptide count had a ΔCn score of at least 0.1. The proteins identified by two or more peptide counts in either non-diabetic or diabetic serum were used to the following bioinformatics analysis.

### Western bolt analysis of C3 and its fragments

Each of 100 µg non-diabetic and diabetic serum-proteins was subjected to PAGE-gel electrophresis, and then proteins in the gel were transferred to a nitrocellulose membrane. The membranes were incubated first with the appropriate primary antibodies (C3b: ab11871, C3a: ab11872, purchased from Abcam Ltd, Cambridge, MA), respectively, and then incubated with HRP-conjugated secondary antibodies for 45 min. The proteins were detected by enhanced chemiluminescence (ECL-plus, Amersham Pharmacia Biotech).

### Validation of ficolin-3 over-representation in larger samples

0.4 uL of each individual serum sample (non-diabetic and diabetic subjects, n = 24, respectively) diluted to 1/10 with 1.0 M Tris (pH 6.8) buffer was separated by SDS-PAGE, and electro-transferred to a nitrocellulose membrane (Whatman International Ltd., England.). The membrane was blotted with a mouse monoclonal antibody against human ficolin-3 (R&D Systems, Inc., 1∶500). Signal detection was achieved with ECL Plus chemiluminescence system (Amersham Biosciences). Signal of bands from Western blot were scanned with PDQUEST GS-710 a flat-bed scanner and digitized with Gel-PRO Analyzer software (Media Cybernetics, Inc., USA). To decrease the system discrepancy, we used the serum of the same patient as the reference. Relative level of serum ficolin-3 was calculated by the proportion of density ratio of sample bands to that of the reference band. These density ratios were used for statistical analyses of serum ficolin-3 level between non-diabetic and diabetic subjects.

### Statistical analysis

Data were expressed as means±standard deviation (SD) for normally distributed values. Differences between groups for normally distributed variables were tested using t-test (analysis of variance). All calculations were performed with GraphPad Prism software system (GraphPad San Diego, CA, USA) and SPSS13.0 statistical package (Statistical Software, Los Angeles, CA, USA). A P value below 0.05 was considered statistically significant.

## Supporting Information

Figure S1 The distribution of proteins and peptides identified in 42 gel slices of non-diabetic serum and diabetic serum
(0.02 MB PDF)

Figure S2 Reproducibility of Gel-LC-MS/MS separations and identification.
(0.21 MB PDF)

Figure S3 Reproducibility and reliability of LSPAD method
(0.15 MB PDF)

Figure S4 The identified proteins and abundance biases in 18 pathways
(0.23 MB PDF)

Figure S5 Western blot analyses of the serum ficolin3 level in the non-diabetic subjects(n = 24)and diabetic patients(n = 24)
(0.14 MB PDF)

Table S1 Baseline characteristics of five non-diabetic subjects and five diabetic patients
(0.02 MB PDF)

Table S2 Proteins identified by two or more peptide spectral counts in non-diabetic and diabetic serum
(0.43 MB PDF)

Table S3 Pathway analysis by mapping 1377 proteins into KEGG pathways. Ratio (%): (100 Ã? Gene number found in pathway) / Totallygene number in pathway. P value: present overall bias of pathways associated with diabetic-serum or non-diabetic serum
(0.08 MB PDF)

Table S4 General and clinical parameters of non-diabetic subjects and type 2 diabetic patients
(0.05 MB PDF)
