# Supplementary material for: Localized-Statistical Quantification of Human Serum Proteome Associated with Type 2 Diabetes
Source: PLoS One. 2008 Sep 16;3(9):e3224. doi: 10.1371/journal.pone.0003224 (PMC2529402; doi:10.1371/journal.pone.0003224)

Supplementary Figure S1. The distribution of proteins and peptides identified in 42 gel slices of non-diabetic serum and diabetic serum.

A

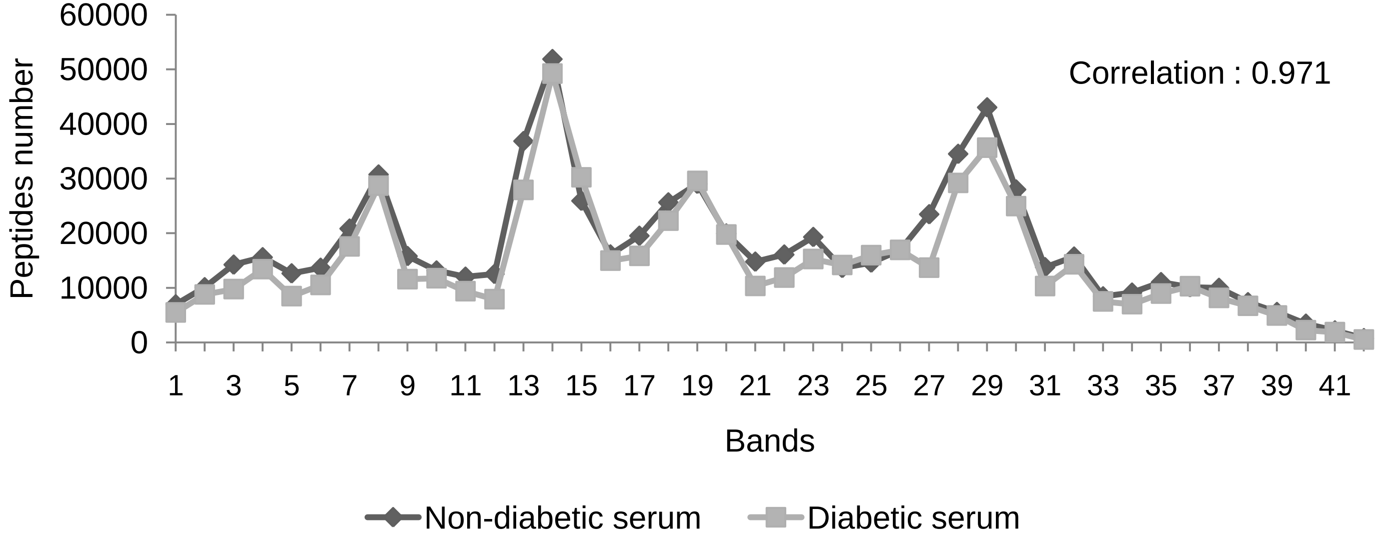

B

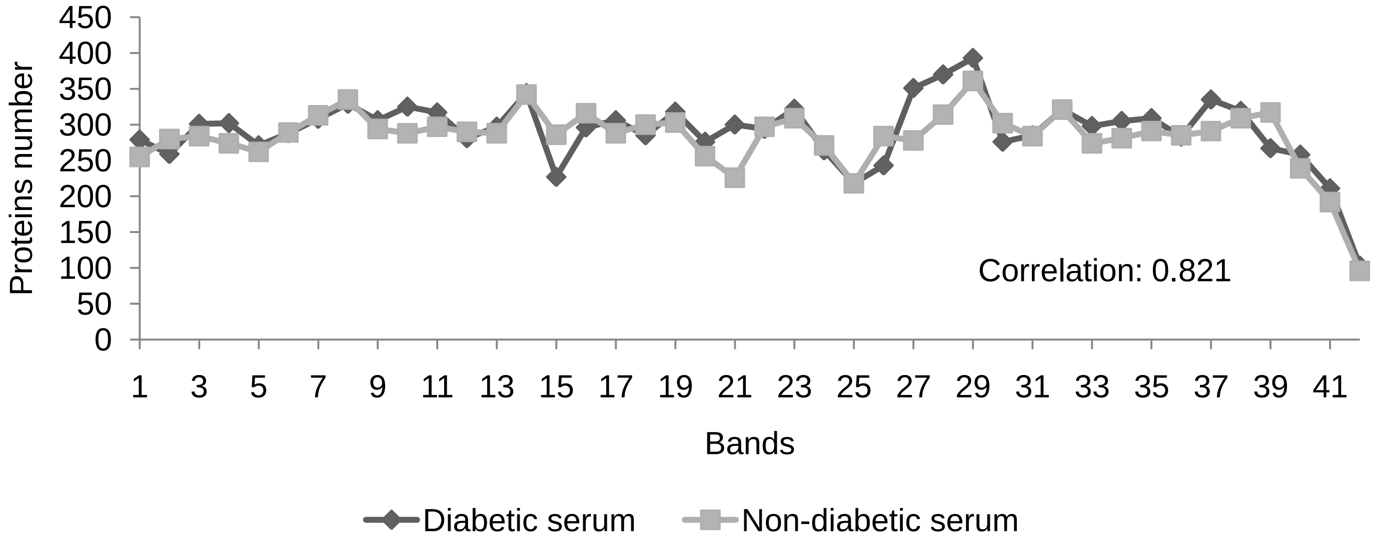

Supplement: Figure S1 — The distribution of proteins and peptides identified in 42 gel slices of non-diabetic serum and diabetic serum (0.02 MB PDF) [file pone.0003224.s001.pdf]
