# Supplementary material for: Localized-Statistical Quantification of Human Serum Proteome Associated with Type 2 Diabetes
Source: PLoS One. 2008 Sep 16;3(9):e3224. doi: 10.1371/journal.pone.0003224 (PMC2529402; doi:10.1371/journal.pone.0003224)

Supplementary Figure S2. Reproducibility of Gel-LC-MS/MS separations and identification. (A) Two duplicates of one pooled-diabetic serum (500 μg) and one pooled-nondiabetic serum (500 μg) were separated on 12% gel electrophoresis, respectively. The entire gel was cut into 12 gel slices. After digestion in gel, the peptide mixture of each slice was processed by mass spectrometry. (B) The number of identified proteins (left y-axis) and peptides (right y-axis) with at least two spectral peptide counts from two diabetic-serum duplicates. (C) The distribution of proteins identified in 12 gel slices between two diabetic-serum duplicates. (D) The distribution of proteins identified in 12 gel slices between two diabetic-serum duplicates. (E) The correlations between the data of duplicate 1 and duplicate 2. Reproducibility of two duplicates of GeLC-MS/MS separations and identifications were assessed based on the distribution of proteins, peptides identified in 12 gel slices, and spectral peptide count information of identified proteins.

Supplementary Figure S2

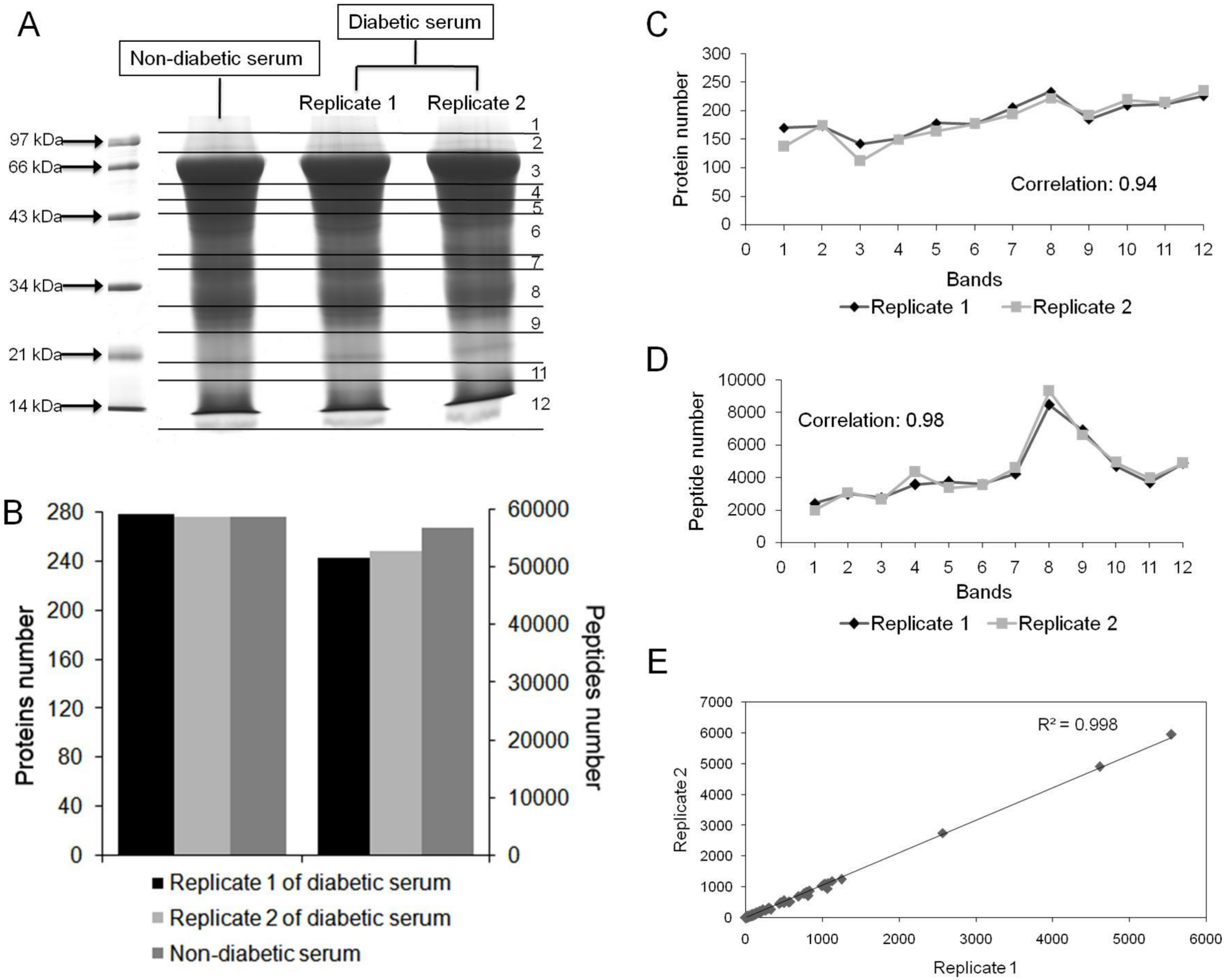

Supplement: Figure S2 — Reproducibility of Gel-LC-MS/MS separations and identification. (0.21 MB PDF) [file pone.0003224.s002.pdf]
