# Supplementary material for: Localized-Statistical Quantification of Human Serum Proteome Associated with Type 2 Diabetes
Source: PLoS One. 2008 Sep 16;3(9):e3224. doi: 10.1371/journal.pone.0003224 (PMC2529402; doi:10.1371/journal.pone.0003224)

Supplementary Figure S3. Reproducibility and reliability of LSPAD method. (A) M-A plotting of identified proteins between two replicates of diabetic serum. “M” was defined as differential protein abundance ratios of each protein between diabetic and diabetic serum, and “A” was defined as protein-abundance of each protein. In addition, ret dots represented statistically significant over-represented proteins in replicate 1 of diabetic serum, green dots represented statistically significant under-represented proteins in replicate 1 of diabetic serum, and grey dots were proteins without statistically-significant change in two cohorts. (B) M-A plotting analysis of replicate 1 of diabetic serum and non-diabetic serum samples. (C) M-A plotting analysis of replicate 2 of diabetic serum and non-diabetic serum samples. (D) The linear correlation analysis of shared significantly changed proteins in B and C based on M value resulting from B (M1) and C (M2). In which, 12 proteins were significantly over-represented in two replicates of diabetic serum compared to non-diabetic serum (more than 0), and night proteins were significantly under-represented in two replicates of diabetic serum compared to non-diabetic serum (less than 0).

Supplementary Figure S3

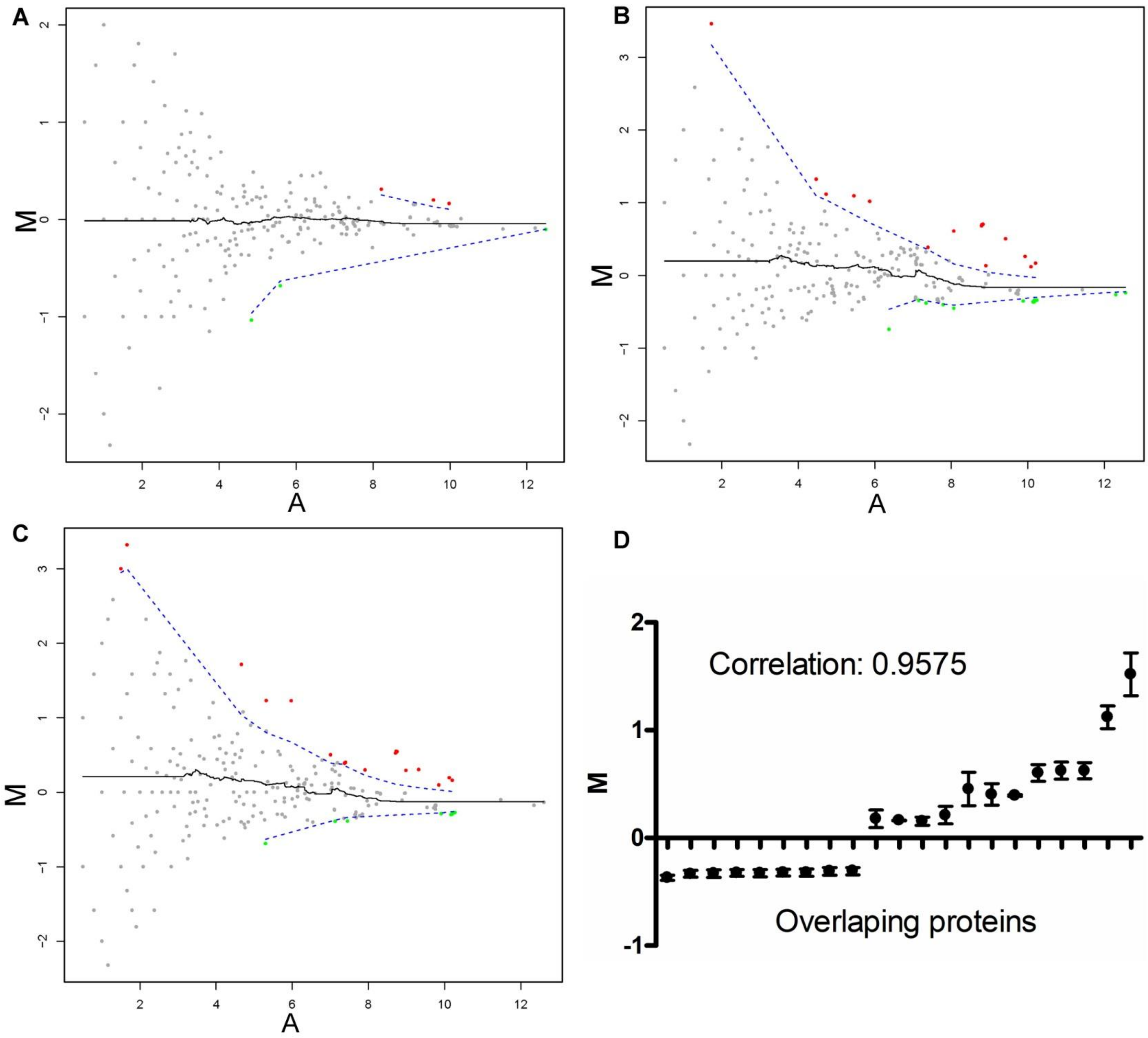

Supplement: Figure S3 — Reproducibility and reliability of LSPAD method (0.15 MB PDF) [file pone.0003224.s003.pdf]
