# Supplementary material for: Localized-Statistical Quantification of Human Serum Proteome Associated with Type 2 Diabetes
Source: PLoS One. 2008 Sep 16;3(9):e3224. doi: 10.1371/journal.pone.0003224 (PMC2529402; doi:10.1371/journal.pone.0003224)
Supplement: Figure S4 — The identified proteins and abundance biases in 18 pathways (0.23 MB PDF) [file pone.0003224.s004.pdf]

Supplementary Figure S4. The identified proteins and abundance biases in 18 pathways. These pathway included Complement system (A), Alzheimer’s disease (B), PPAR signaling pathway (C), Cell communication (D), Hematopoietic cell lineage (E), Folate biosynthesis (F), Adipocytokine signaling pathway (G), Inositol phosphate metabolism (H), Type II diabetes mellitus (I), Fatty acid metabolism (J), Arginine and praline metabolism (K), ECM-receptor interaction (L), TGF-beta signaling pathway (M), Coagulation cascade (N), Small cell lung cancer (O), Insulin signaling pathway (P), Focal adhesion (Q), Long-term depression (R). The p value of identified protein was digitized to the length of the bar in each pathway.

Supplementary Figure S4

A

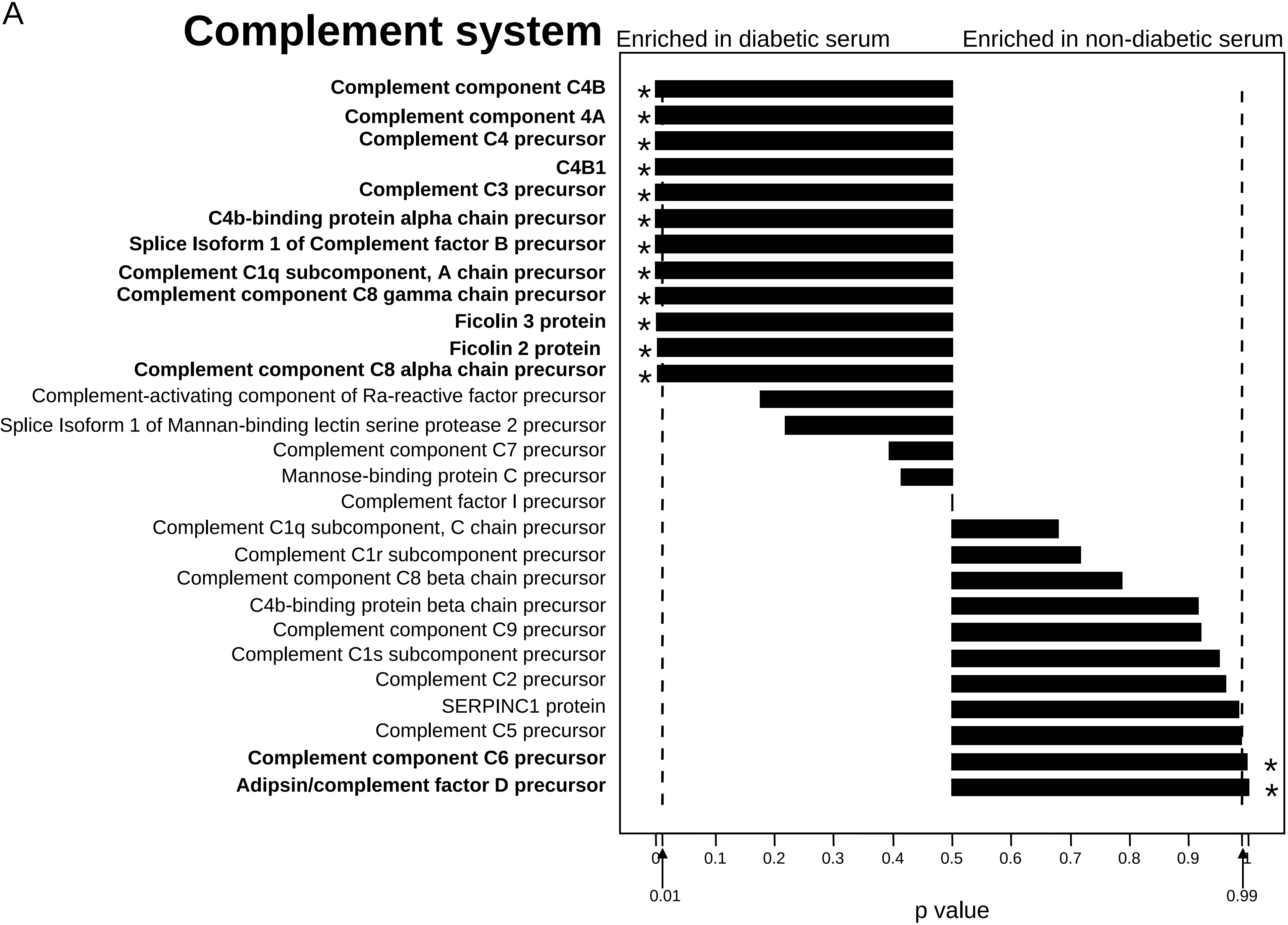

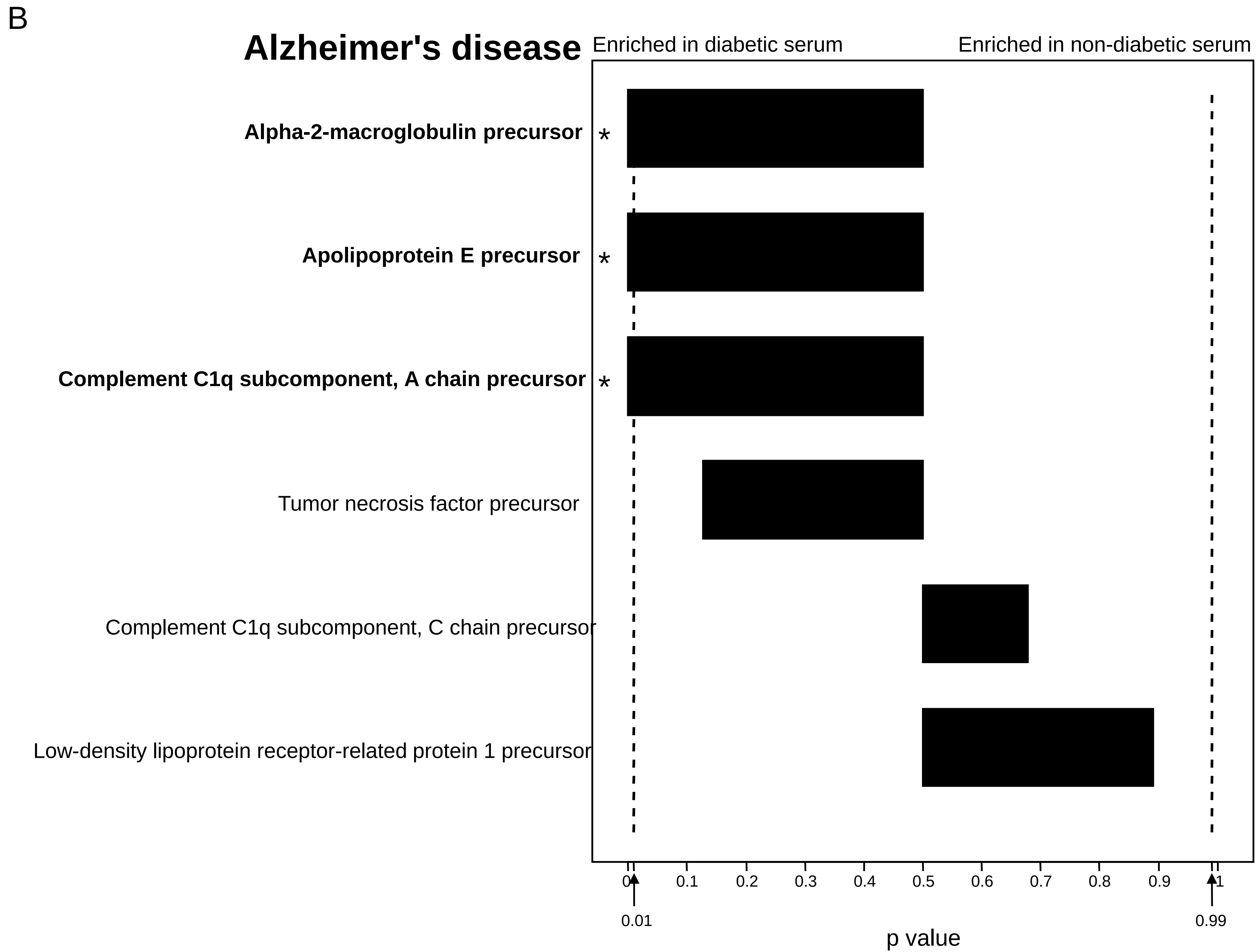
B
Alzheimer's disease
Enriched in diabetic serum
Enriched in non-diabetic serum
Alpha-2-macroglobulin precursor *
Apolipoprotein E precursor *
Complement C1q subcomponent, A chain precursor *
Tumor necrosis factor precursor
Complement C1q subcomponent, C chain precursor
Low-density lipoprotein receptor-related protein 1 precursor
0
0.1
0.2
0.3
0.4
0.5
0.6
0.7
0.8
0.9
1
0.01
0.99
p value

C

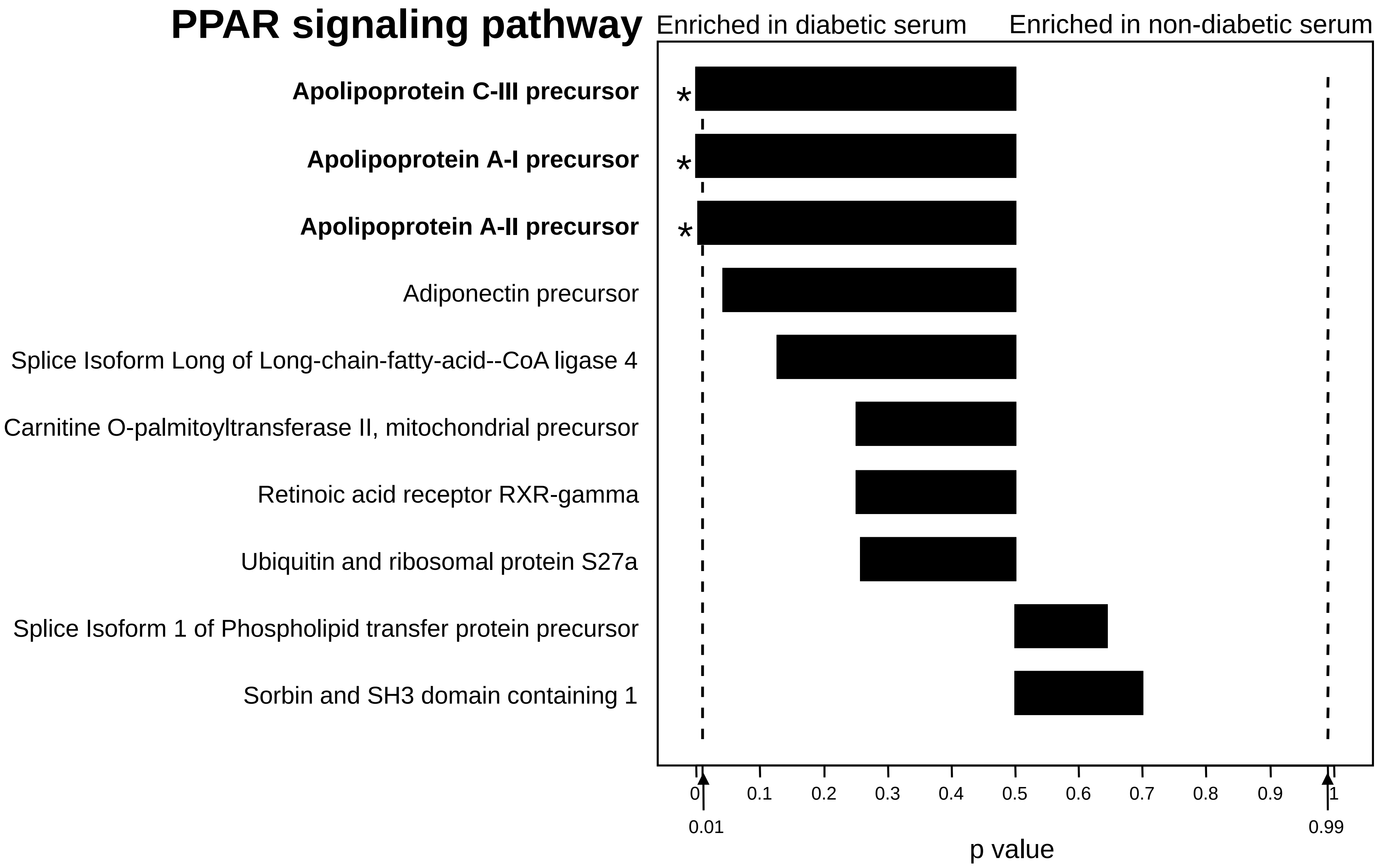
PPAR signaling pathway
Enriched in diabetic serum
Enriched in non-diabetic serum
Apolipoprotein C-III precursor
Apolipoprotein A-I precursor
Apolipoprotein A-II precursor
Adiponectin precursor
Splice Isoform Long of Long-chain-fatty-acid--CoA ligase 4
Carnitine O-palmitoyltransferase II, mitochondrial precursor
Retinoic acid receptor RXR-gamma
Ubiquitin and ribosomal protein S27a
Splice Isoform 1 of Phospholipid transfer protein precursor
Sorbin and SH3 domain containing 1
*
*
*
0
0.1
0.2
0.3
0.4
0.5
0.6
0.7
0.8
0.9
1
0.01
0.99
p value

D

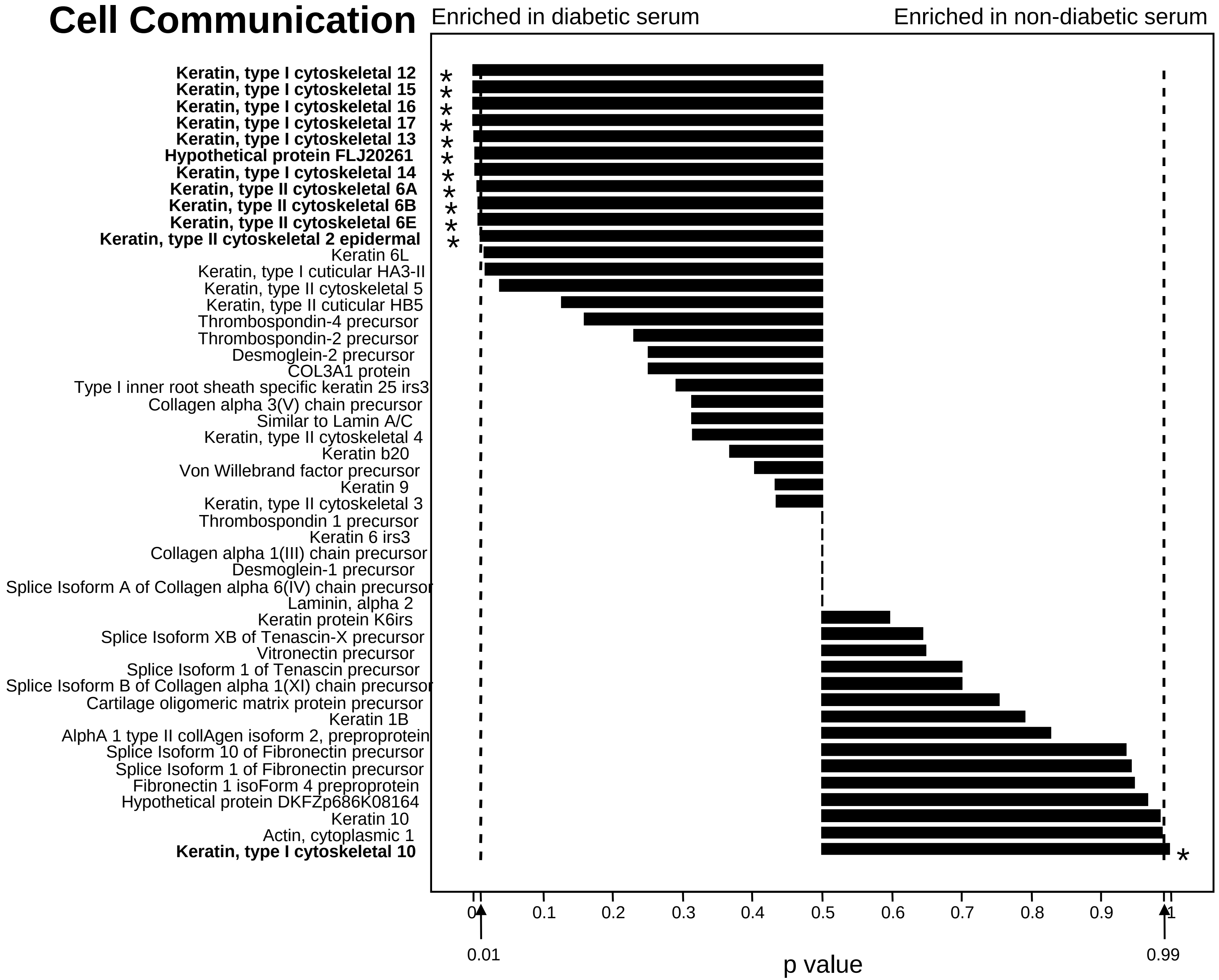

Cell Communication
Enriched in diabetic serum
Enriched in non-diabetic serum
Keratin, type I cytoskeletal 12
Keratin, type I cytoskeletal 15
Keratin, type I cytoskeletal 16
Keratin, type I cytoskeletal 17
Keratin, type I cytoskeletal 13
Hypothetical protein FLJ20261
Keratin, type I cytoskeletal 14
Keratin, type II cytoskeletal 6A
Keratin, type II cytoskeletal 6B
Keratin, type II cytoskeletal 6E
Keratin, type II cytoskeletal 2 epidermal
Keratin 6L
Keratin, type I cuticular HA3-II
Keratin, type II cytoskeletal 5
Keratin, type II cuticular HB5
Thrombospondin-4 precursor
Thrombospondin-2 precursor
Desmoglein-2 precursor
COL3A1 protein
Type I inner root sheath specific keratin 25 irs3
Collagen alpha 3(V) chain precursor
Similar to Lamin A/C
Keratin, type II cytoskeletal 4
Keratin b20
Von Willebrand factor precursor
Keratin 9
Keratin, type II cytoskeletal 3
Thrombospondin 1 precursor
Keratin 6 irs3
Collagen alpha 1(III) chain precursor
Desmoglein-1 precursor
Splice Isoform A of Collagen alpha 6(IV) chain precursor
Laminin, alpha 2
Keratin protein K6irs
Splice Isoform XB of Tenascin-X precursor
Vitronectin precursor
Splice Isoform 1 of Tenascin precursor
Splice Isoform B of Collagen alpha 1(XI) chain precursor
Cartilage oligomeric matrix protein precursor
Keratin 1B
AlphA 1 type II collAgen isoform 2, preproprotein
Splice Isoform 10 of Fibronectin precursor
Splice Isoform 1 of Fibronectin precursor
Fibronectin 1 isoForm 4 preproprotein
Hypothetical protein DKFZp686K08164
Keratin 10
Actin, cytoplasmic 1
Keratin, type I cytoskeletal 10
0
0.1
0.2
0.3
0.4
0.5
0.6
0.7
0.8
0.9
1
0.01
0.99
p value

E

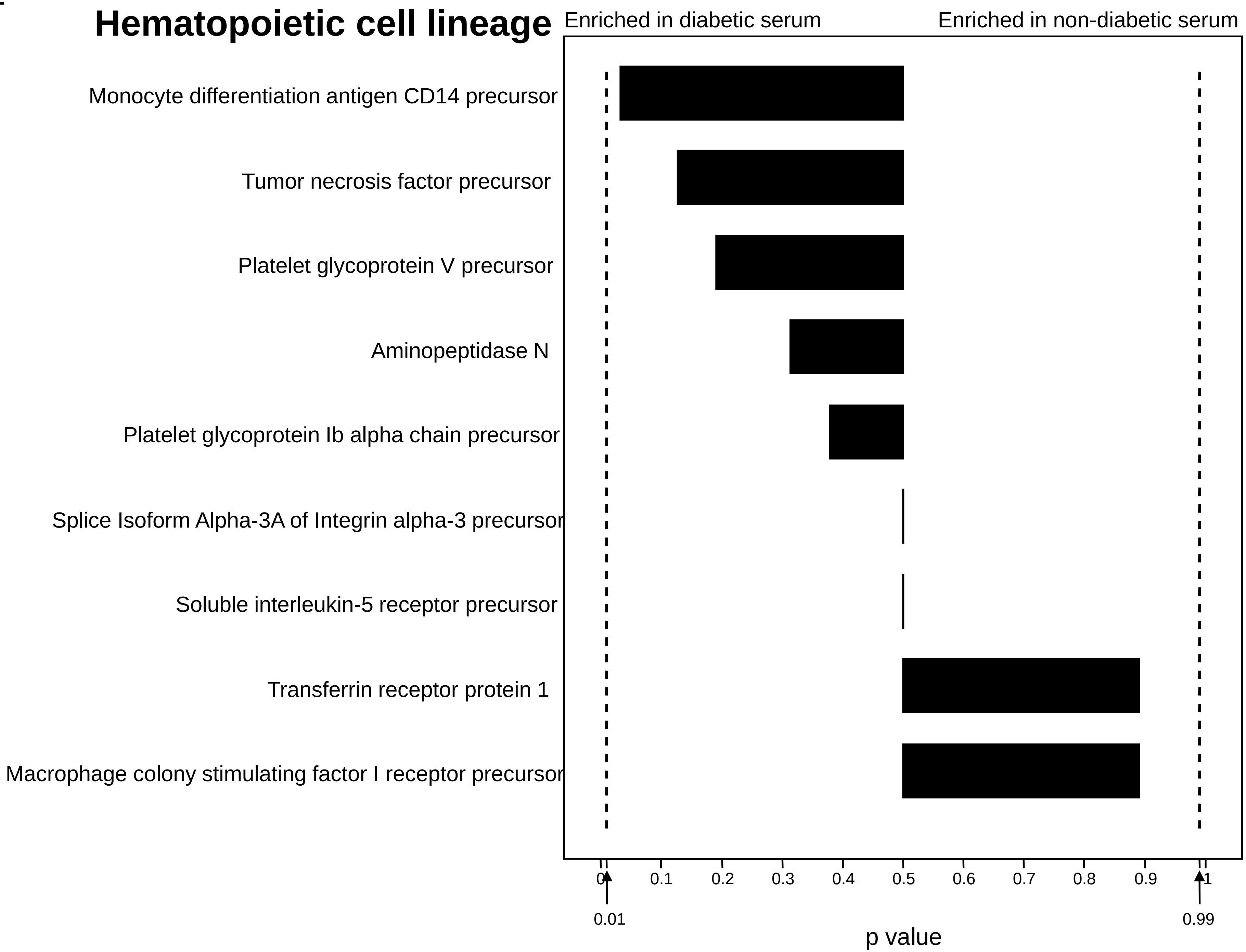

Hematopoietic cell lineage
Enriched in diabetic serum
Enriched in non-diabetic serum
Monocyte differentiation antigen CD14 precursor
Tumor necrosis factor precursor
Platelet glycoprotein V precursor
Aminopeptidase N
Platelet glycoprotein Ib alpha chain precursor
Splice Isoform Alpha-3A of Integrin alpha-3 precursor
Soluble interleukin-5 receptor precursor
Transferrin receptor protein 1
Macrophage colony stimulating factor I receptor precursor
0
0.1
0.2
0.3
0.4
0.5
0.6
0.7
0.8
0.9
1
0.01
0.99
p value

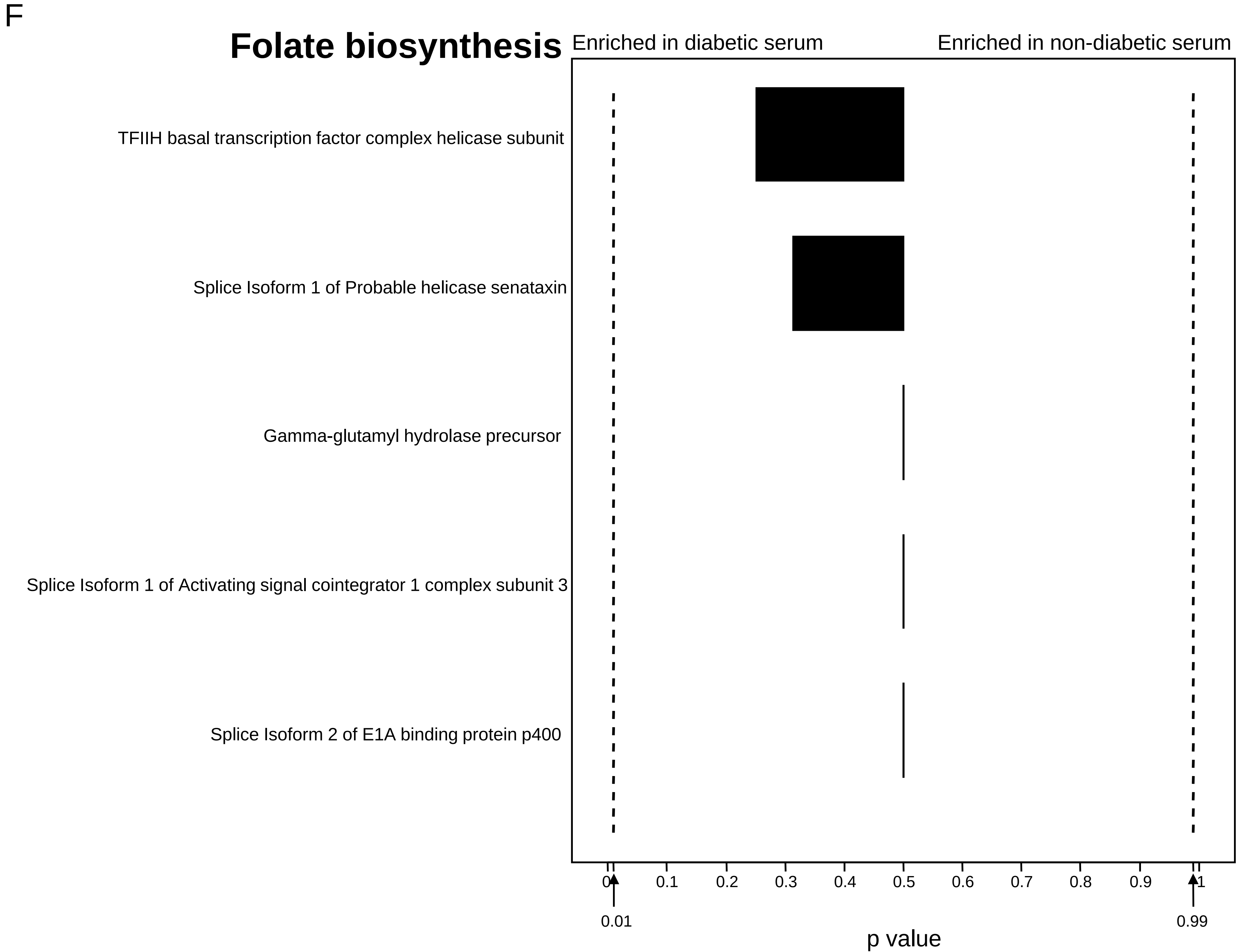
F
Folate biosynthesis
Enriched in diabetic serum
Enriched in non-diabetic serum
TFIIH basal transcription factor complex helicase subunit
Splice Isoform 1 of Probable helicase senataxin
Gamma-glutamyl hydrolase precursor
Splice Isoform 1 of Activating signal cointegrator 1 complex subunit 3
Splice Isoform 2 of E1A binding protein p400
0
0.1
0.2
0.3
0.4
0.5
0.6
0.7
0.8
0.9
1
0.01
0.99
p value

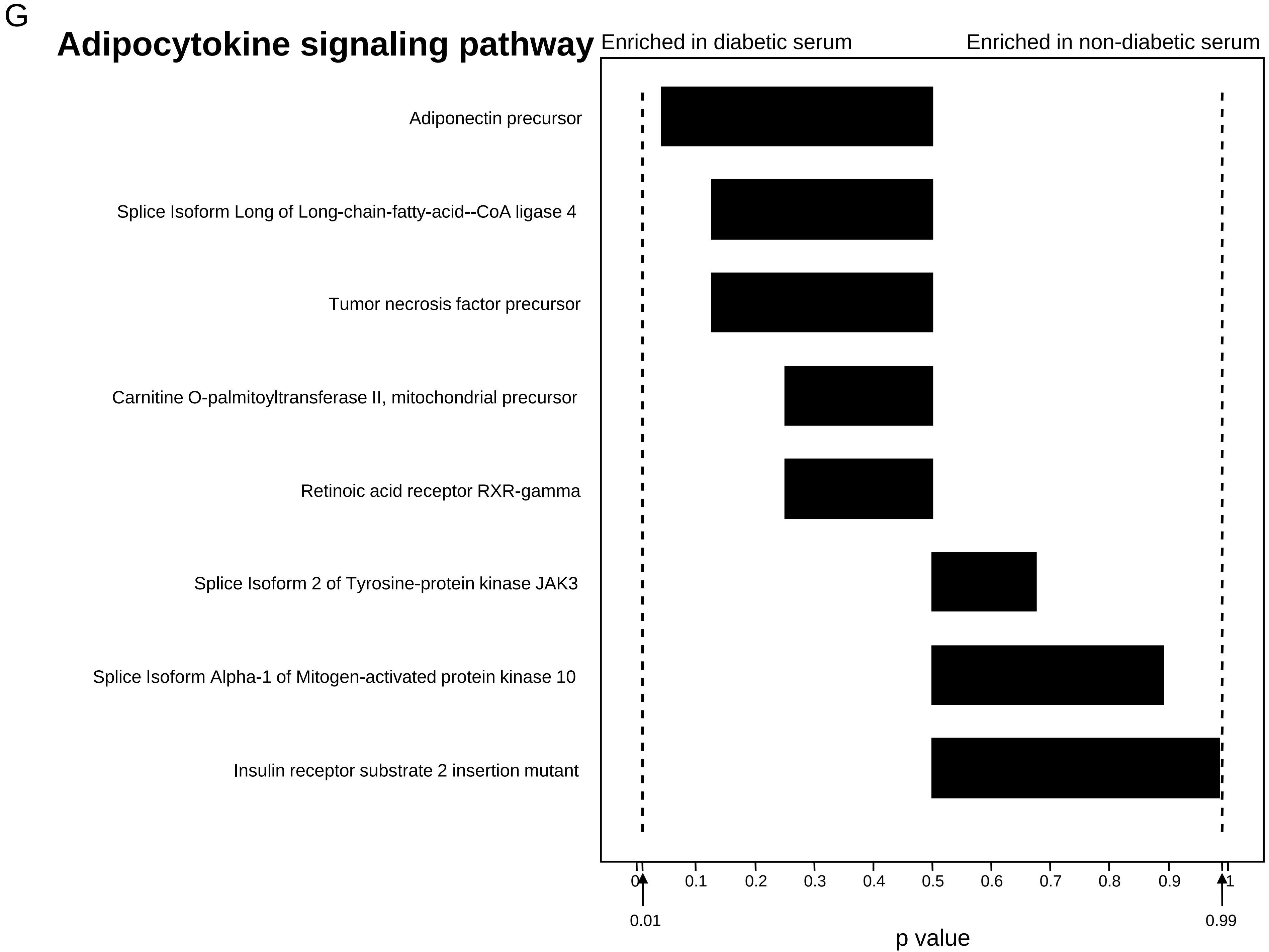
G
Adipocytokine signaling pathway
Enriched in diabetic serum
Enriched in non-diabetic serum
Adiponectin precursor
Splice Isoform Long of Long-chain-fatty-acid--CoA ligase 4
Tumor necrosis factor precursor
Carnitine O-palmitoyltransferase II, mitochondrial precursor
Retinoic acid receptor RXR-gamma
Splice Isoform 2 of Tyrosine-protein kinase JAK3
Splice Isoform Alpha-1 of Mitogen-activated protein kinase 10
Insulin receptor substrate 2 insertion mutant
0
0.1
0.2
0.3
0.4
0.5
0.6
0.7
0.8
0.9
1
0.01
0.99
p value

H

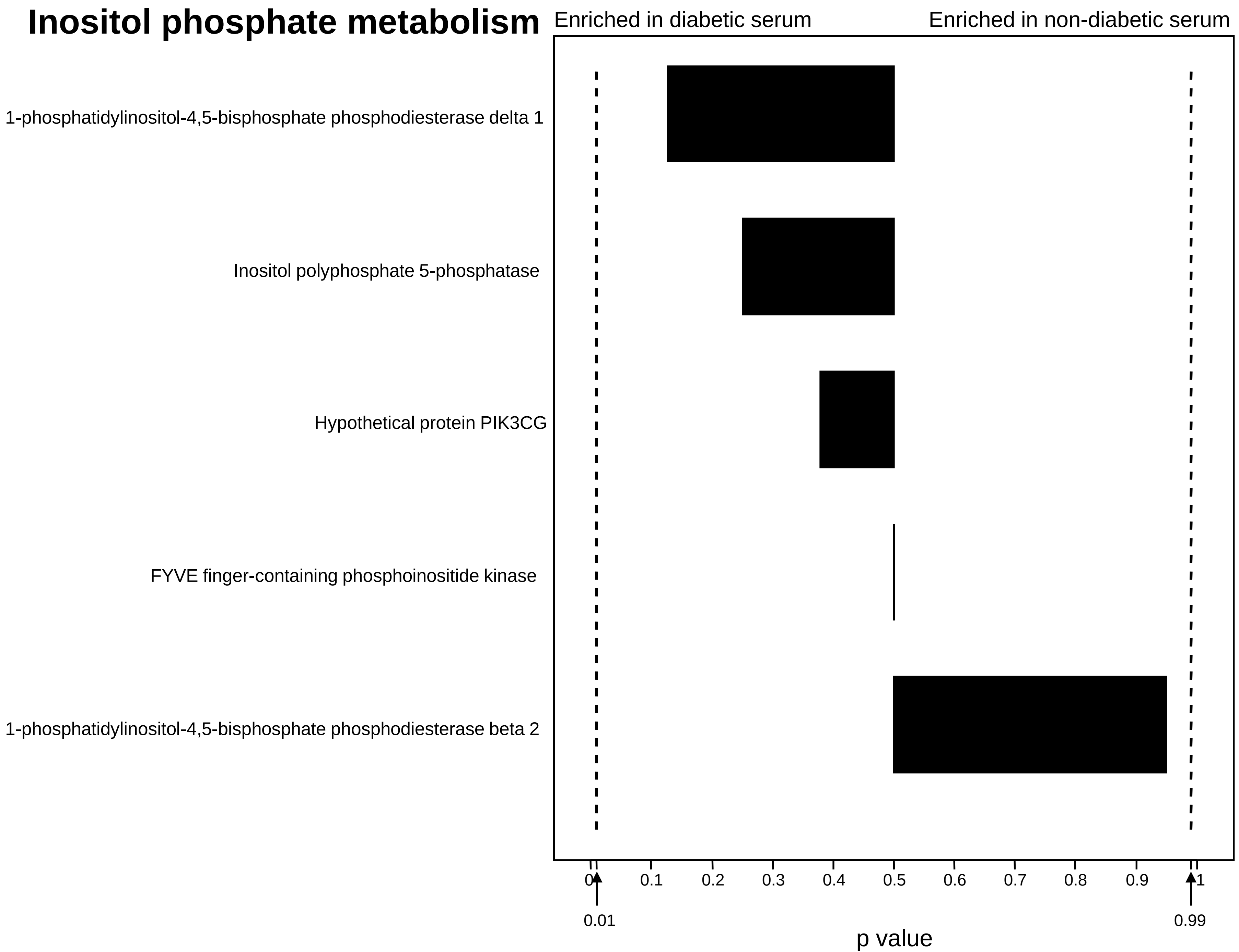
Inosititol phosphate metabolism
Enriched in diabetic serum
Enriched in non-diabetic serum
1-phosphatidylinositol-4,5-bisphosphate phosphodiesterase delta 1
Inositol polyphosphate 5-phosphatase
Hypothetical protein PIK3CG
FYVE finger-containing phosphoinositide kinase
1-phosphatidylinositol-4,5-bisphosphate phosphodiesterase beta 2
0
0.1
0.2
0.3
0.4
0.5
0.6
0.7
0.8
0.9
1
0.01
0.99
p value

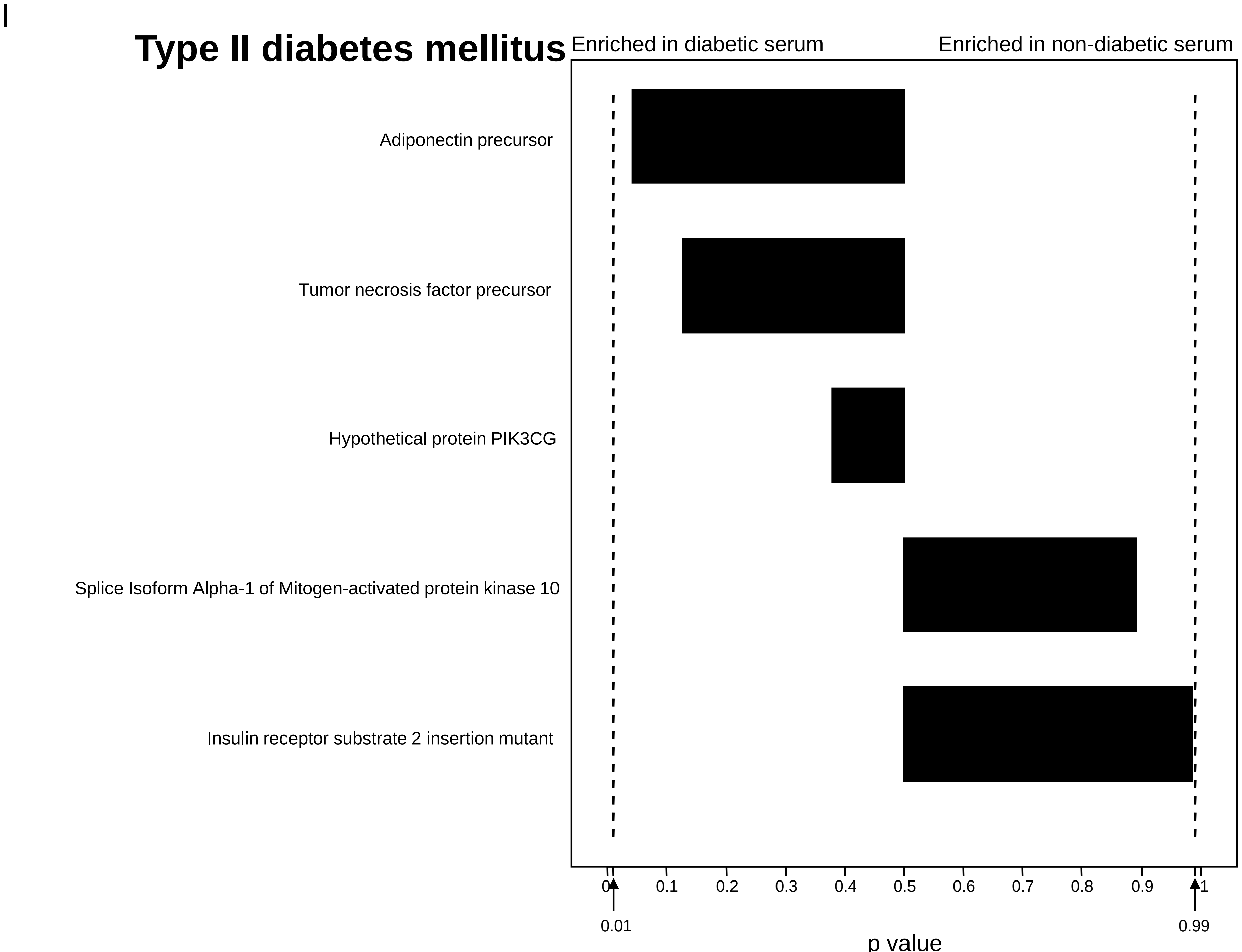
I
Type II diabetes mellitus
Enriched in diabetic serum
Enriched in non-diabetic serum
Adiponectin precursor
Tumor necrosis factor precursor
Hypothetical protein PIK3CG
Splice Isoform Alpha-1 of Mitogen-activated protein kinase 10
Insulin receptor substrate 2 insertion mutant
0
0.1
0.2
0.3
0.4
0.5
0.6
0.7
0.8
0.9
1
0.01
0.99
p value

J

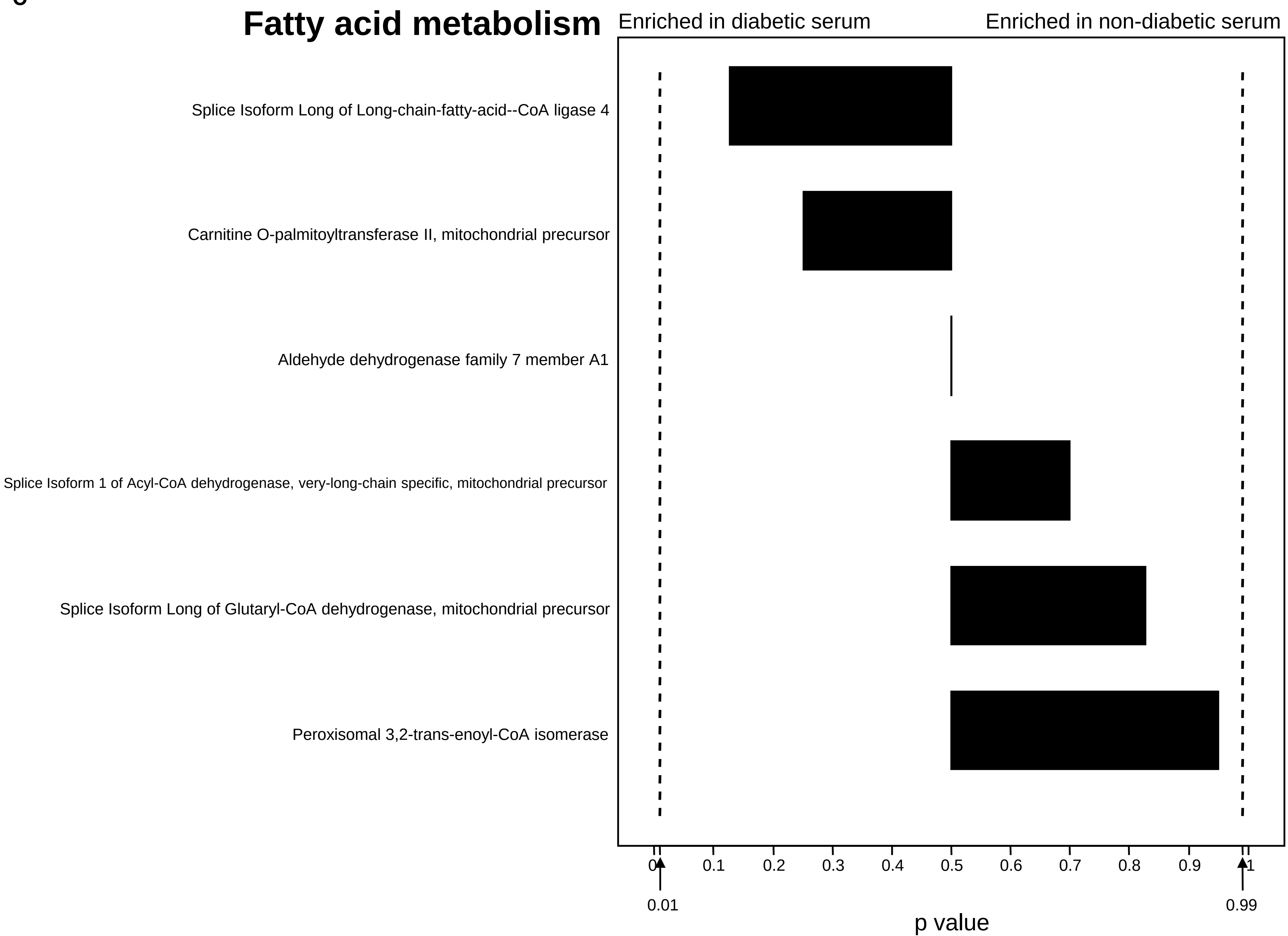
Fatty acid metabolism
Enriched in diabetic serum
Enriched in non-diabetic serum
Splice Isoform Long of Long-chain-fatty-acid--CoA ligase 4
Carnitine O-palmitoyltransferase II, mitochondrial precursor
Aldehyde dehydrogenase family 7 member A1
Splice Isoform 1 of Acyl-CoA dehydrogenase, very-long-chain specific, mitochondrial precursor
Splice Isoform Long of Glutaryl-CoA dehydrogenase, mitochondrial precursor
Peroxisomal 3,2-trans-enoyl-CoA isomerase
0
0.1
0.2
0.3
0.4
0.5
0.6
0.7
0.8
0.9
1
0.01
0.99
p value

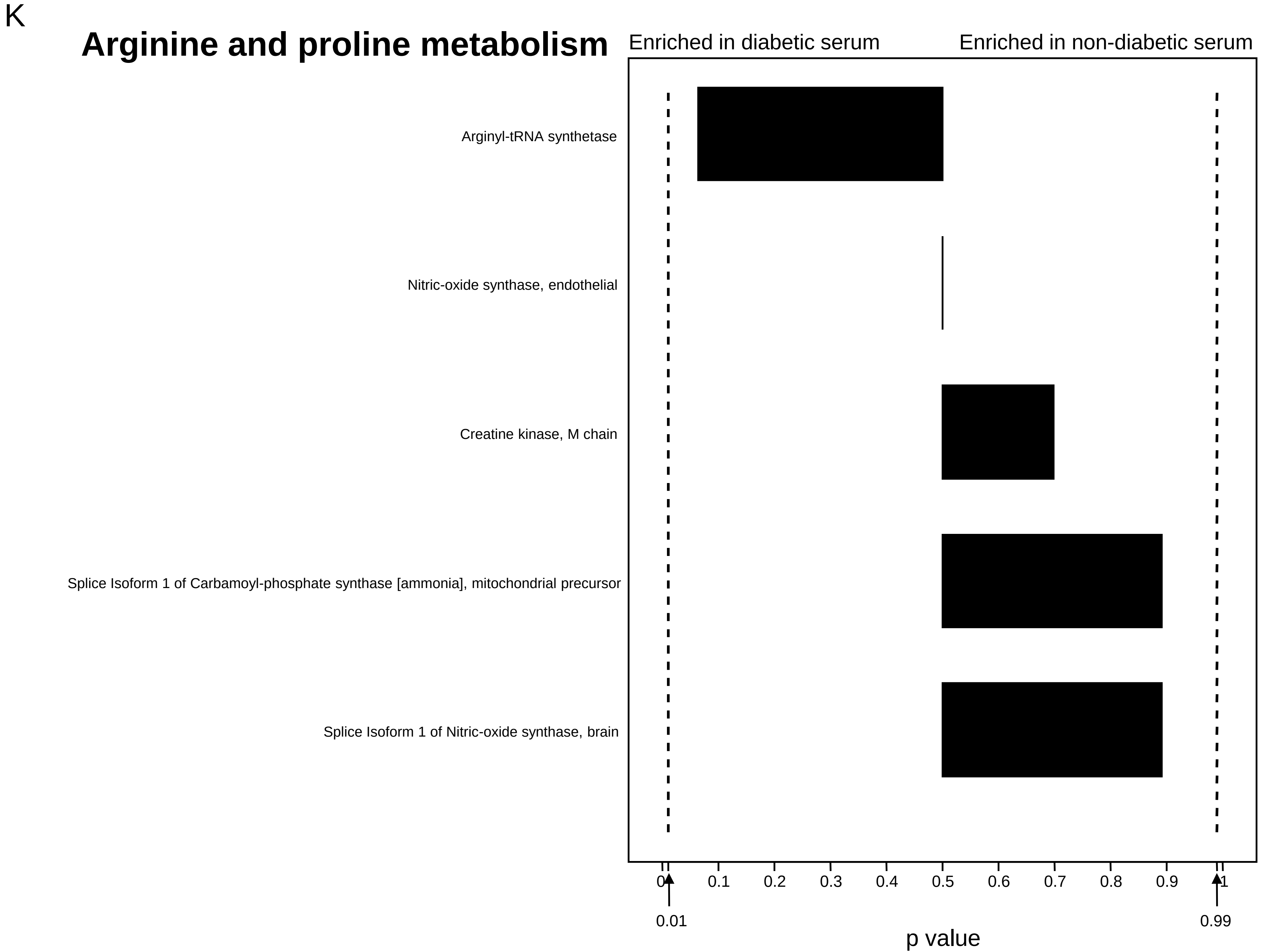
K
Arginine and proline metabolism
Enriched in diabetic serum
Enriched in non-diabetic serum
Arginyl-tRNA synthetase
Nitric-oxide synthase, endothelial
Creatine kinase, M chain
Splice Isoform 1 of Carbamoyl-phosphate synthase [ammonia], mitochondrial precursor
Splice Isoform 1 of Nitric-oxide synthase, brain
0
0.1
0.2
0.3
0.4
0.5
0.6
0.7
0.8
0.9
1
0.01
0.99
p value

L

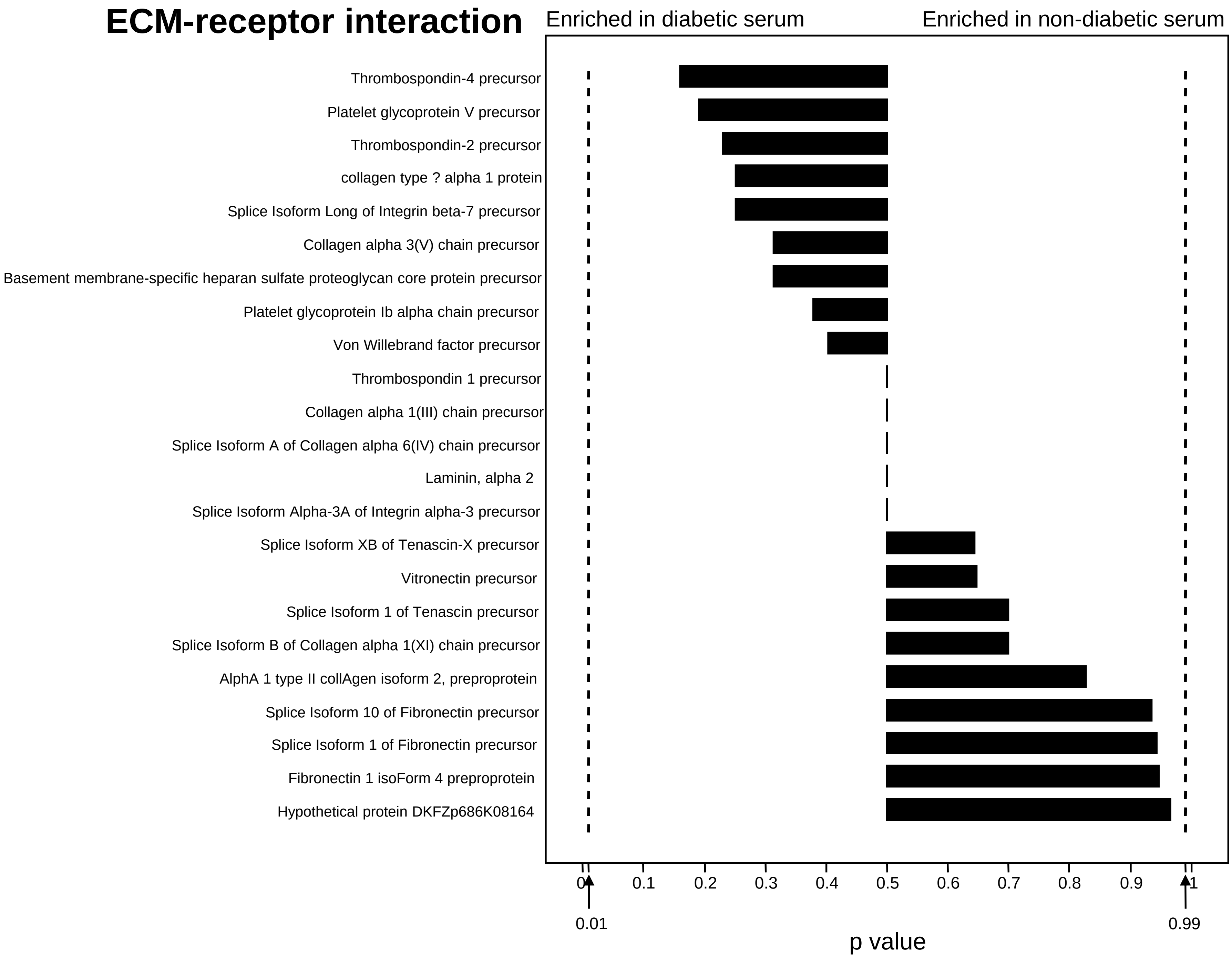
ECM-receptor interaction
Enriched in diabetic serum
Enriched in non-diabetic serum
Thrombospondin-4 precursor
Platelet glycoprotein V precursor
Thrombospondin-2 precursor
collagen type ? alpha 1 protein
Splice Isoform Long of Integrin beta-7 precursor
Collagen alpha 3(V) chain precursor
Basement membrane-specific heparan sulfate proteoglycan core protein precursor
Platelet glycoprotein Ib alpha chain precursor
Von Willebrand factor precursor
Thrombospondin 1 precursor
Collagen alpha 1(III) chain precursor
Splice Isoform A of Collagen alpha 6(IV) chain precursor
Laminin, alpha 2
Splice Isoform Alpha-3A of Integrin alpha-3 precursor
Splice Isoform XB of Tenascin-X precursor
Vitronectin precursor
Splice Isoform 1 of Tenascin precursor
Splice Isoform B of Collagen alpha 1(XI) chain precursor
AlphA 1 type II collAgen isoform 2, preproprotein
Splice Isoform 10 of Fibronectin precursor
Splice Isoform 1 of Fibronectin precursor
Fibronectin 1 isoForm 4 preproprotein
Hypothetical protein DKFZp686K08164
0
0.1
0.2
0.3
0.4
0.5
0.6
0.7
0.8
0.9
1
0.01
0.99
p value

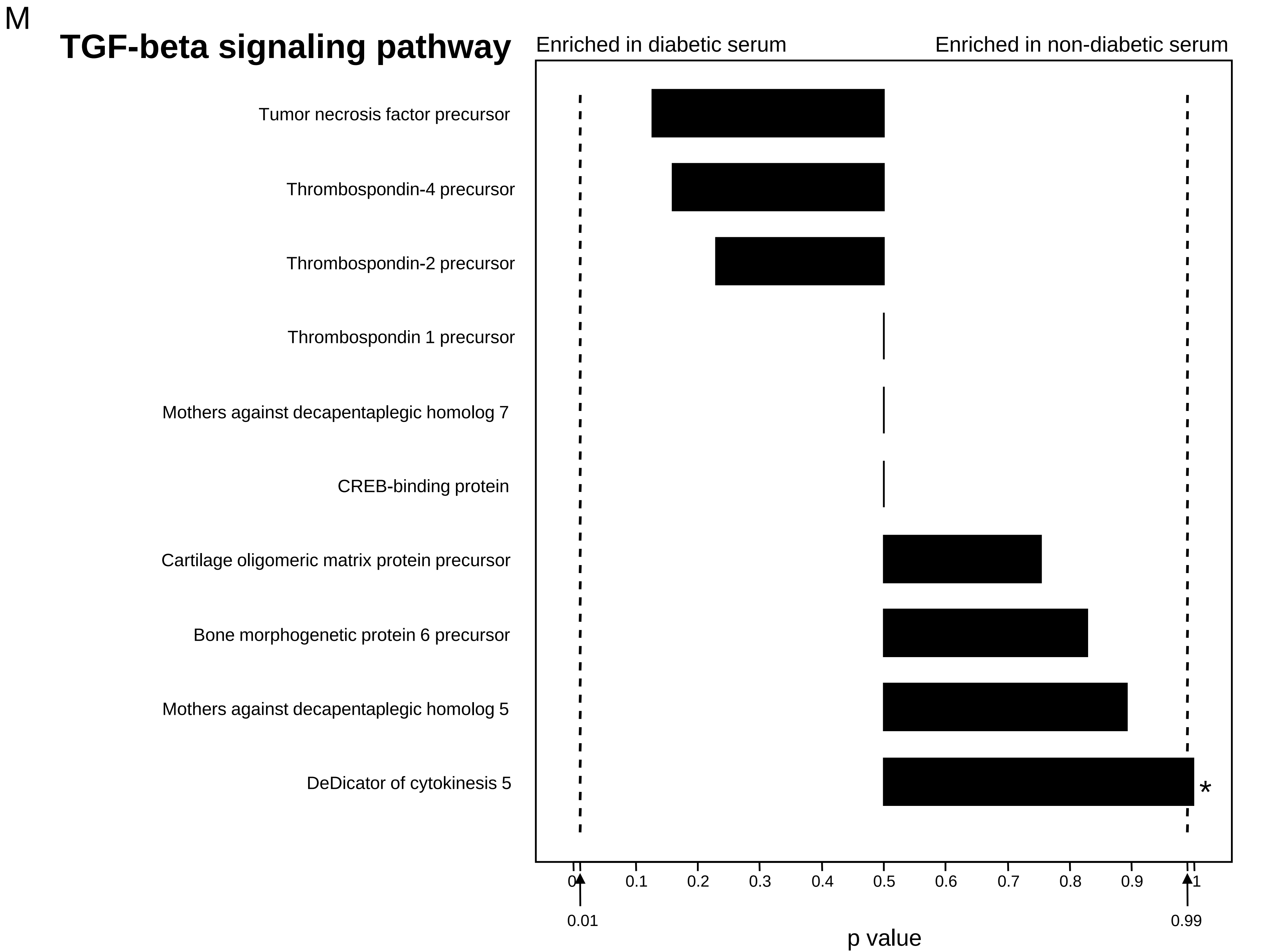
M
TGF-beta signaling pathway
Enriched in diabetic serum
Enriched in non-diabetic serum
Tumor necrosis factor precursor
Thrombospondin-4 precursor
Thrombospondin-2 precursor
Thrombospondin 1 precursor
Mothers against decapentaplegic homolog 7
CREB-binding protein
Cartilage oligomeric matrix protein precursor
Bone morphogenetic protein 6 precursor
Mothers against decapentaplegic homolog 5
DeDicator of cytokinesis 5
*
0
0.1
0.2
0.3
0.4
0.5
0.6
0.7
0.8
0.9
1
0.01
0.99
p value

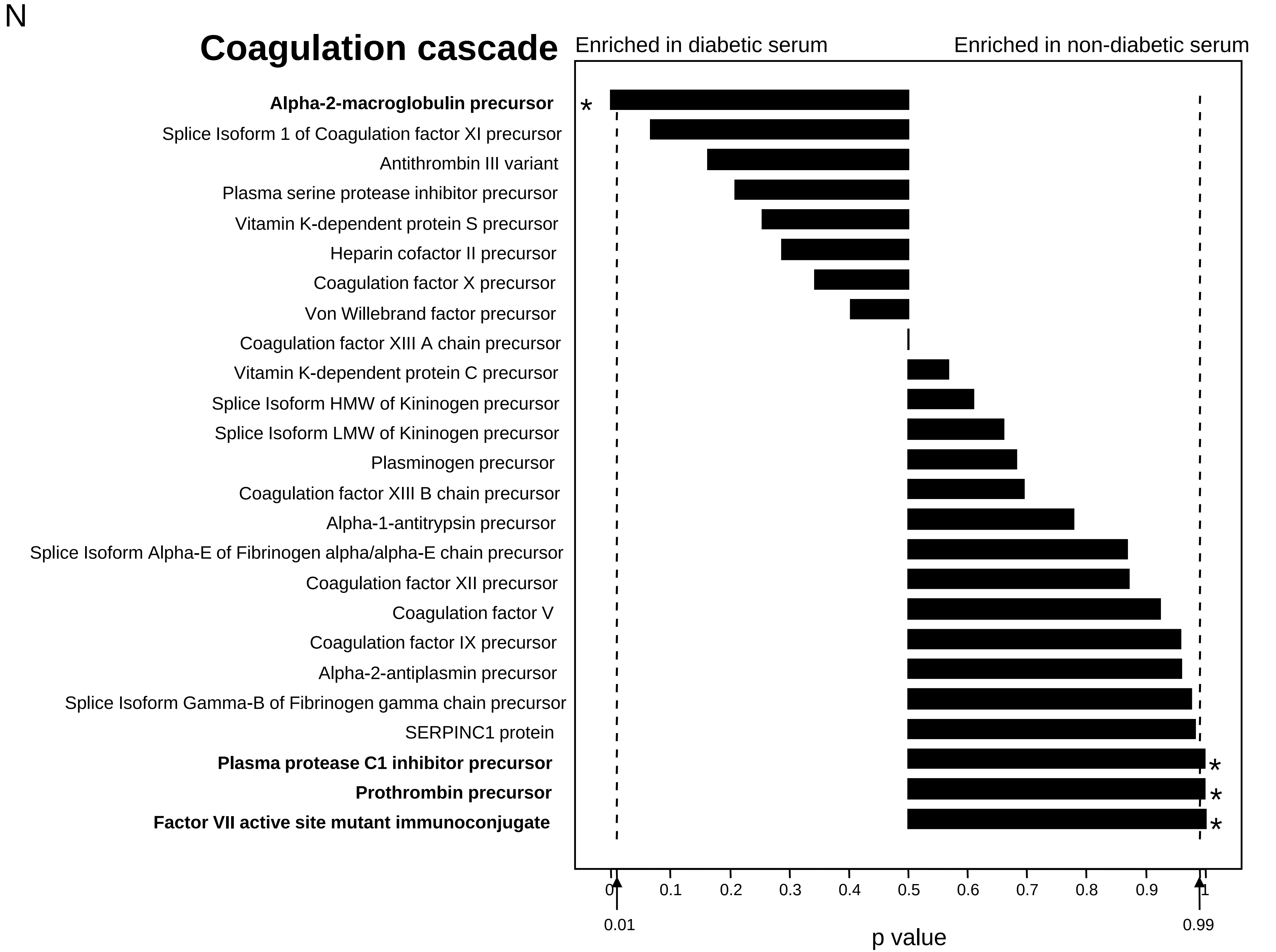
N
Coagulation cascade
Enriched in diabetic serum
Enriched in non-diabetic serum
Alpha-2-macroglobulin precursor *
Splice Isoform 1 of Coagulation factor XI precursor
Antithrombin III variant
Plasma serine protease inhibitor precursor
Vitamin K-dependent protein S precursor
Heparin cofactor II precursor
Coagulation factor X precursor
Von Willebrand factor precursor
Coagulation factor XIII A chain precursor
Vitamin K-dependent protein C precursor
Splice Isoform HMW of Kininogen precursor
Splice Isoform LMW of Kininogen precursor
Plasminogen precursor
Coagulation factor XIII B chain precursor
Alpha-1-antitrypsin precursor
Splice Isoform Alpha-E of Fibrinogen alpha/alpha-E chain precursor
Coagulation factor XII precursor
Coagulation factor V
Coagulation factor IX precursor
Alpha-2-antiplasmin precursor
Splice Isoform Gamma-B of Fibrinogen gamma chain precursor
SERPINC1 protein
Plasma protease C1 inhibitor precursor *
Prothrombin precursor *
Factor VII active site mutant immunoconjugate *
0
0.1
0.2
0.3
0.4
0.5
0.6
0.7
0.8
0.9
1
0.01
0.99
p value

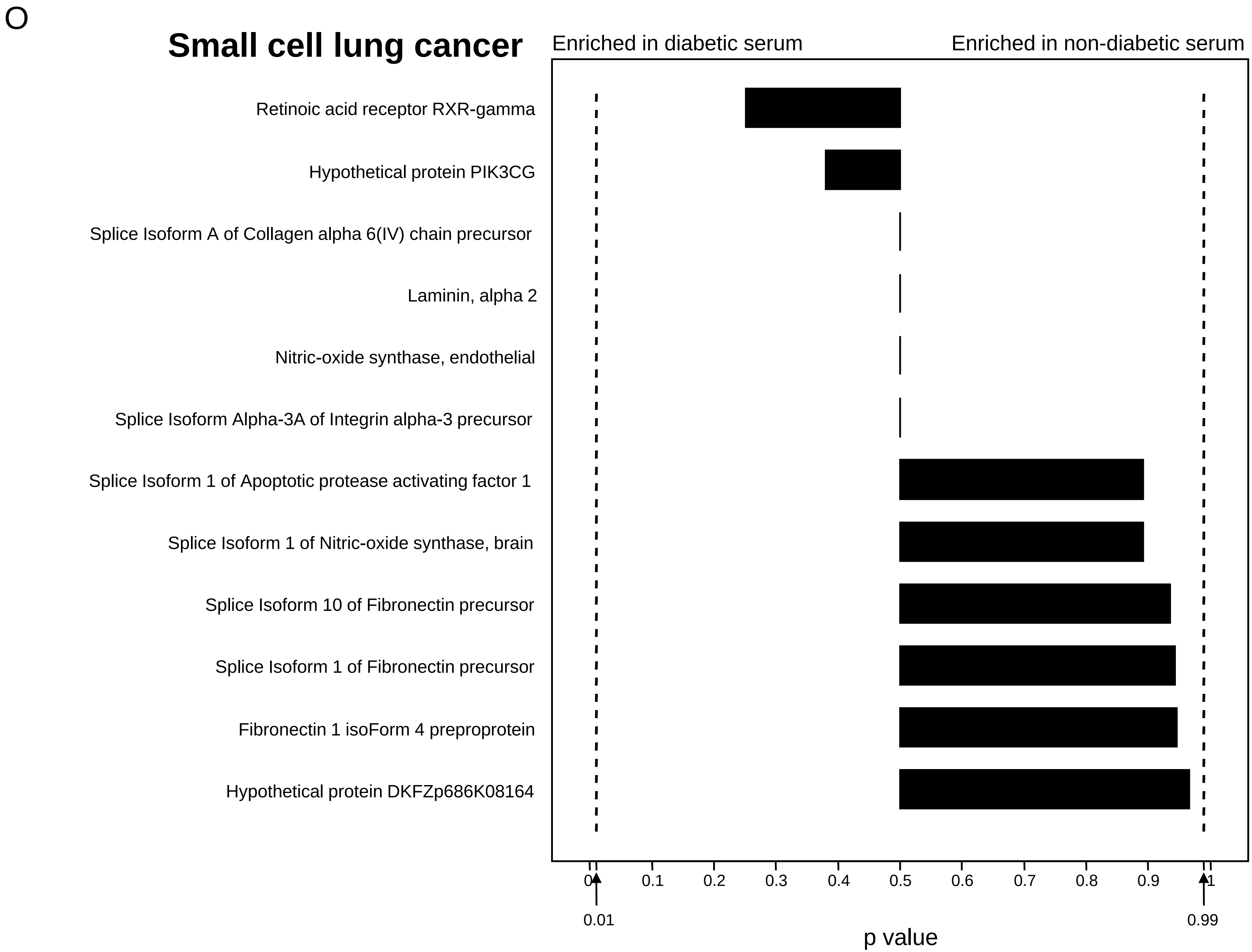
O
Small cell lung cancer
Enriched in diabetic serum
Enriched in non-diabetic serum
Retinoic acid receptor RXR-gamma
Hypothetical protein PIK3CG
Splice Isoform A of Collagen alpha 6(IV) chain precursor
Laminin, alpha 2
Nitric-oxide synthase, endothelial
Splice Isoform Alpha-3A of Integrin alpha-3 precursor
Splice Isoform 1 of Apoptotic protease activating factor 1
Splice Isoform 1 of Nitric-oxide synthase, brain
Splice Isoform 10 of Fibronectin precursor
Splice Isoform 1 of Fibronectin precursor
Fibronectin 1 isoForm 4 preproprotein
Hypothetical protein DKFZp686K08164
0
0.1
0.2
0.3
0.4
0.5
0.6
0.7
0.8
0.9
1
0.01
0.99
p value

P

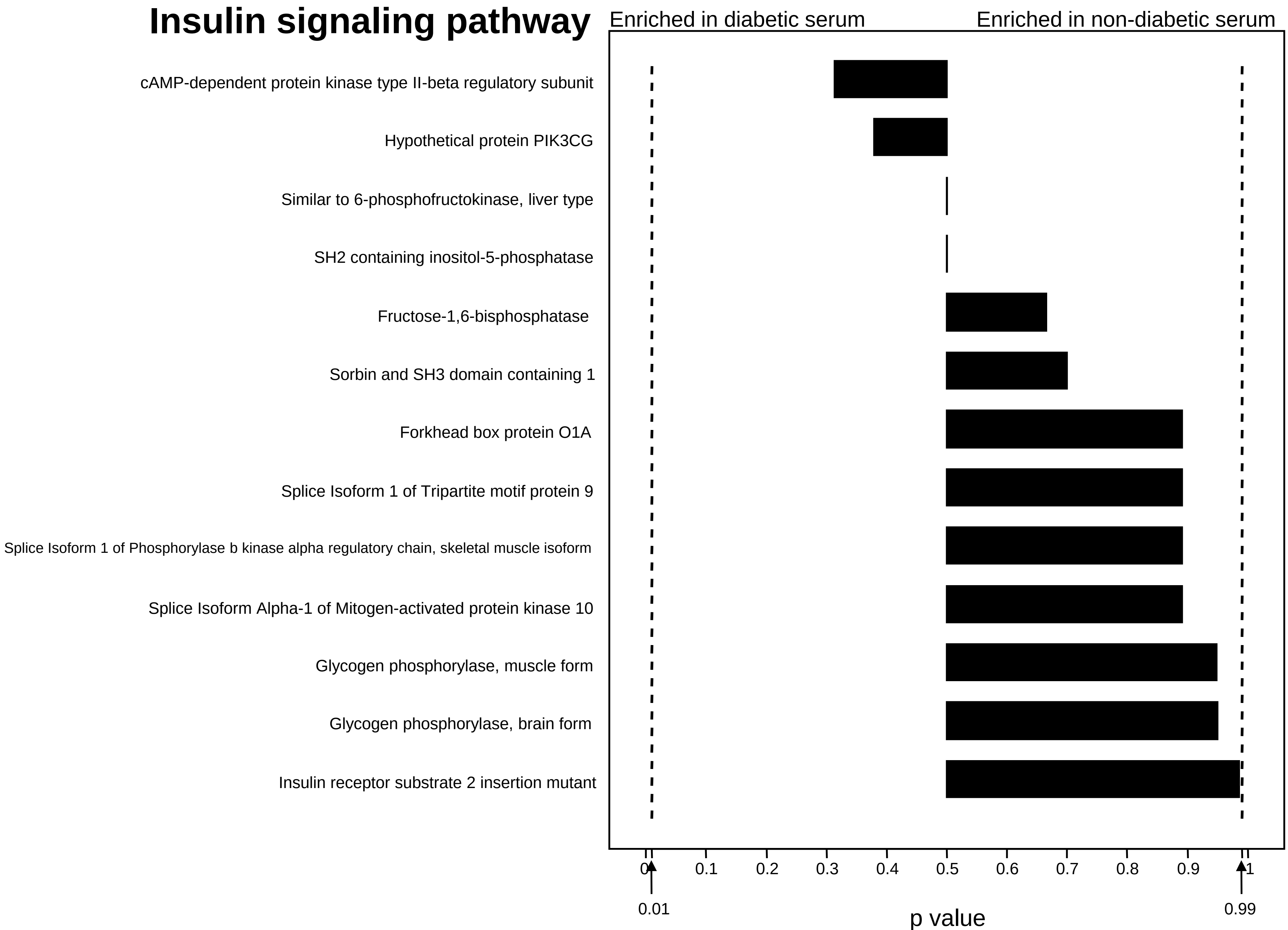
Insulin signaling pathway
Enriched in diabetic serum
Enriched in non-diabetic serum
cAMP-dependent protein kinase type II-beta regulatory subunit
Hypothetical protein PIK3CG
Similar to 6-phosphofructokinase, liver type
SH2 containing inositol-5-phosphatase
Fructose-1,6-bisphosphatase
Sorbin and SH3 domain containing 1
Forkhead box protein O1A
Splice Isoform 1 of Tripartite motif protein 9
Splice Isoform 1 of Phosphorylase b kinase alpha regulatory chain, skeletal muscle isoform
Splice Isoform Alpha-1 of Mitogen-activated protein kinase 10
Glycogen phosphorylase, muscle form
Glycogen phosphorylase, brain form
Insulin receptor substrate 2 insertion mutant
0
0.1
0.2
0.3
0.4
0.5
0.6
0.7
0.8
0.9
1
0.01
0.99
p value

Q

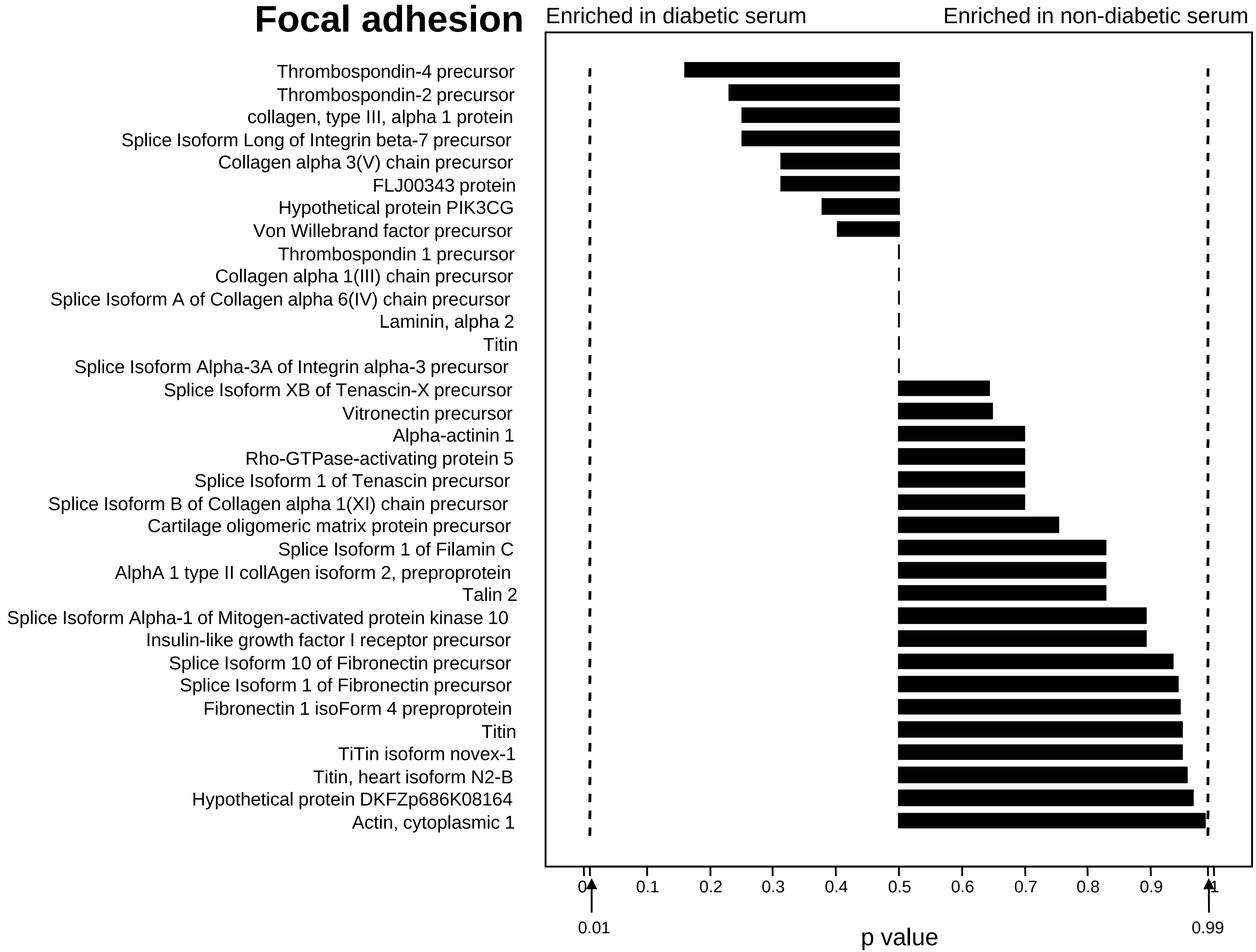

Focal adhesion
Enriched in diabetic serum
Enriched in non-diabetic serum
Thrombospondin-4 precursor
Thrombospondin-2 precursor
collagen, type III, alpha 1 protein
Splice Isoform Long of Integrin beta-7 precursor
Collagen alpha 3(V) chain precursor
FLJ00343 protein
Hypothetical protein PIK3CG
Von Willebrand factor precursor
Thrombospondin 1 precursor
Collagen alpha 1(III) chain precursor
Splice Isoform A of Collagen alpha 6(IV) chain precursor
Laminin, alpha 2
Titin
Splice Isoform Alpha-3A of Integrin alpha-3 precursor
Splice Isoform XB of Tenascin-X precursor
Vitronectin precursor
Alpha-actinin 1
Rho-GTPase-activating protein 5
Splice Isoform 1 of Tenascin precursor
Splice Isoform B of Collagen alpha 1(XI) chain precursor
Cartilage oligomeric matrix protein precursor
Splice Isoform 1 of Filamin C
AlphA 1 type II collAgen isoform 2, preproprotein
Talin 2
Splice Isoform Alpha-1 of Mitogen-activated protein kinase 10
Insulin-like growth factor I receptor precursor
Splice Isoform 10 of Fibronectin precursor
Splice Isoform 1 of Fibronectin precursor
Fibronectin 1 isoForm 4 preproprotein
Titin
TiTin isoform novex-1
Titin, heart isoform N2-B
Hypothetical protein DKFZp686K08164
Actin, cytoplasmic 1
0
0.1
0.2
0.3
0.4
0.5
0.6
0.7
0.8
0.9
1
0.01
0.99
p value

R

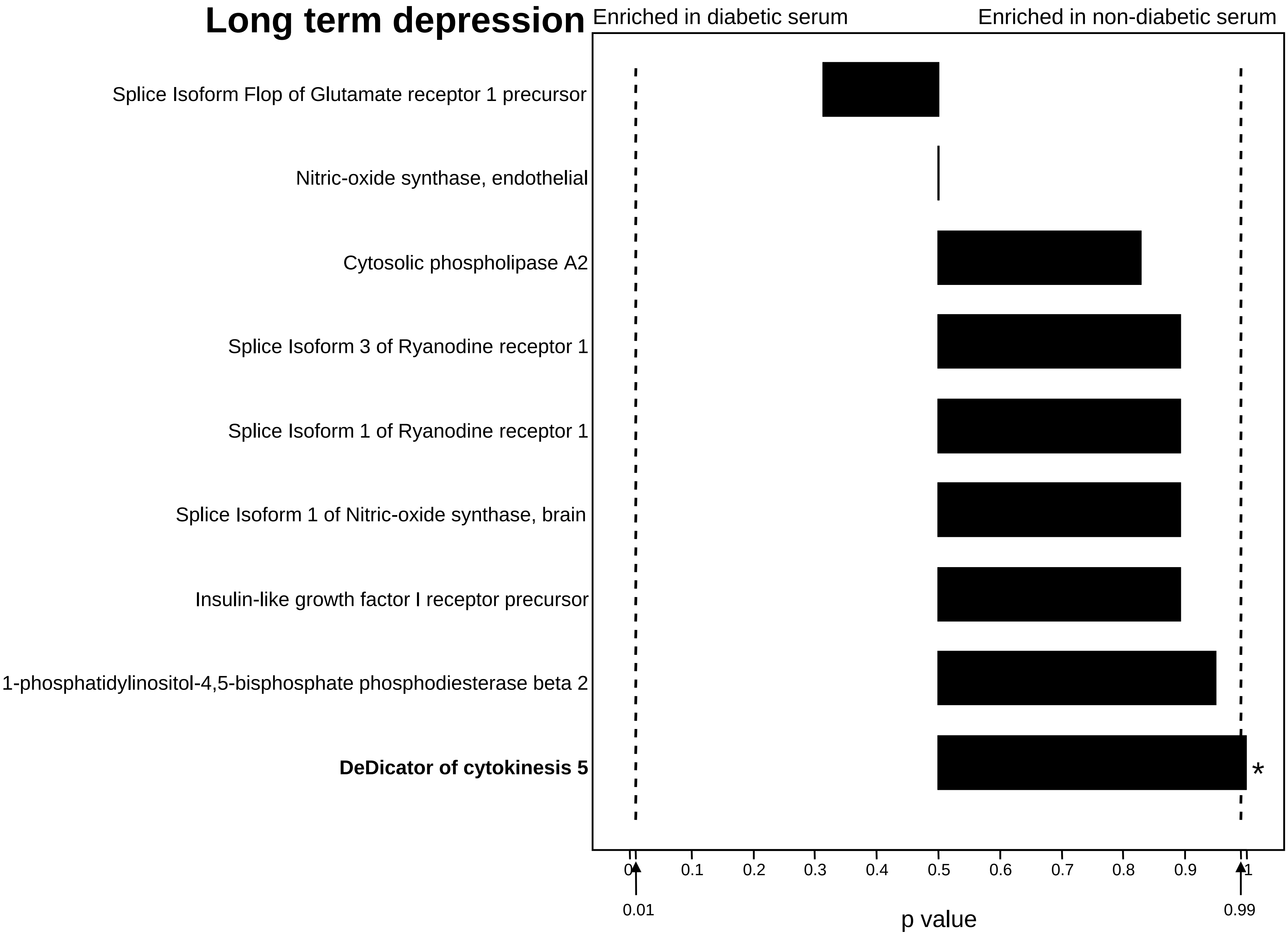

Long term depression
Enriched in diabetic serum
Enriched in non-diabetic serum
Splice Isoform Flop of Glutamate receptor 1 precursor
Nitric-oxide synthase, endothelial
Cytosolic phospholipase A2
Splice Isoform 3 of Ryanodine receptor 1
Splice Isoform 1 of Ryanodine receptor 1
Splice Isoform 1 of Nitric-oxide synthase, brain
Insulin-like growth factor I receptor precursor
1-phosphatidylinositol-4,5-bisphosphate phosphodiesterase beta 2
DeDicator of cytokinesis 5
*
0
0.1
0.2
0.3
0.4
0.5
0.6
0.7
0.8
0.9
1
0.01
0.99
p value
