# Supplementary material for: Localized-Statistical Quantification of Human Serum Proteome Associated with Type 2 Diabetes
Source: PLoS One. 2008 Sep 16;3(9):e3224. doi: 10.1371/journal.pone.0003224 (PMC2529402; doi:10.1371/journal.pone.0003224)

Supplementary Figure S5. Western blot analyses of the serum ficolin3 level in the non-diabetic subjects (n = 24) and diabetic patients (n = 24). “Con” represents the control serum sample from a diabetic patient; “N” represents non-diabetic subjects; “D” represents diabetic patients.

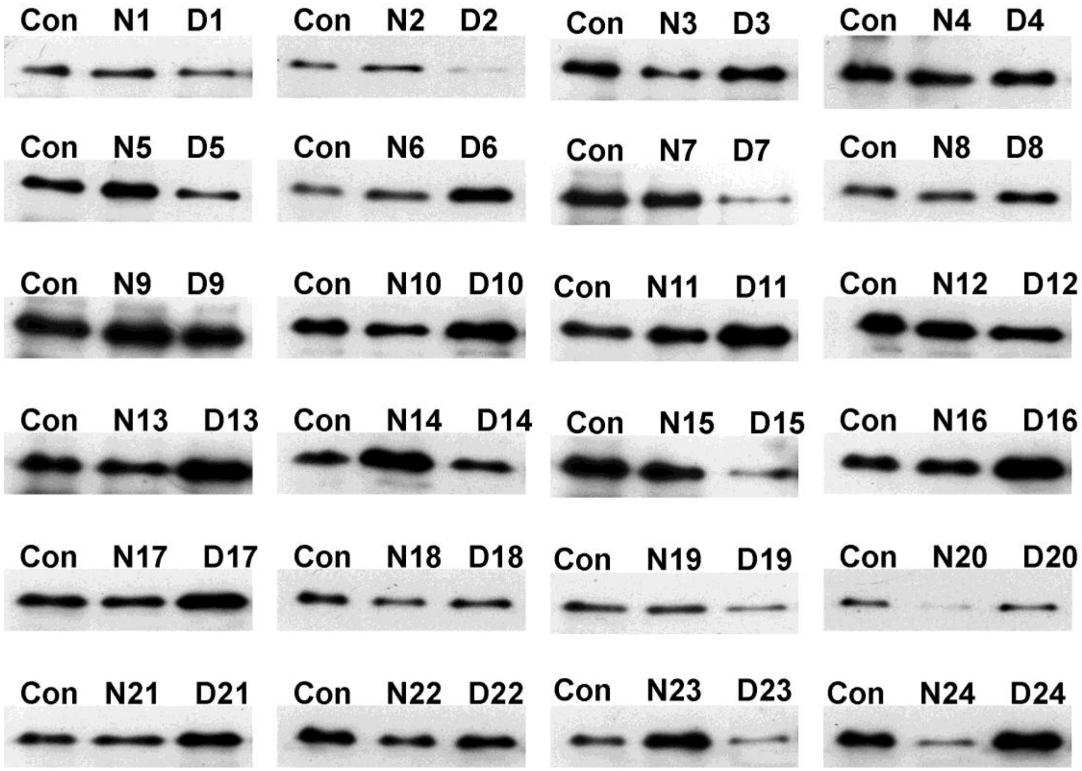

Supplement: Figure S5 — Western blot analyses of the serum ficolin3 level in the non-diabetic subjects(n = 24)and diabetic patients(n = 24) (0.14 MB PDF) [file pone.0003224.s005.pdf]
