# Supplementary material for: Localized-Statistical Quantification of Human Serum Proteome Associated with Type 2 Diabetes
Source: PLoS One. 2008 Sep 16;3(9):e3224. doi: 10.1371/journal.pone.0003224 (PMC2529402; doi:10.1371/journal.pone.0003224)
Supplement: Table S1 — Baseline characteristics of five non-diabetic subjects and five diabetic patients (0.02 MB PDF) [file pone.0003224.s006.pdf]

Supplementary Table S1

Baseline characteristics of five non-diabetic subjects and five diabetic patients. WT: weight (kg), FPG: fasting plasma glucose (mmol/L), PG2H: 2-hour plasma glucose (mmol/L), BMI: Body Mass Index, HbA1c: glycosylated hemoglobin (%); HOMA: The Homeostasis Model Assessment

Diabetic patients	AGE	WT	FPG	PG2H	BMI	HOMA	HbA1c	C-Peptide	With diabetes history
1	66	62.5	12	N.A	22.41	5.49	7.1	1.8	Yes
2	68	93	6.7	13.6	33.95	12.05	7.8	5.45	No
3	68	78.5	6.1	11.6	26.85	5.96	6.9	3.24	No
4	65	74.5	5	11.4	26.08	3.11	7.2	3.12	No
5	68	79.4	6.5	N.A	25.87	1.75	6.7	3.57	Yes
**Non-diabetic subjects**	**AGE**	**WT**	**FPG**	**PG2H**	**BMI**	**HOMA**	**HbA1c**	**C-Peptide**	
1	69	68	5.1	6	22.41	2.12	6.1	1.38	N.A
2	65	59.5	4.9	2.5	20.83	1.24	5.2	1.27	N.A
3	69	62	4.8	6.1	21.58	0.85	5	1.14	N.A
4	68	57.2	4.5	5.4	22.59	0.62	6	0.89	N.A
5	67	57.7	4.8	3.9	20.94	0.4	6.1	0.78	N.A
