# Supplementary material for: Localized-Statistical Quantification of Human Serum Proteome Associated with Type 2 Diabetes
Source: PLoS One. 2008 Sep 16;3(9):e3224. doi: 10.1371/journal.pone.0003224 (PMC2529402; doi:10.1371/journal.pone.0003224)
Supplement: Table S2 — Proteins identified by two or more peptide spectral counts in non-diabetic and diabetic serum (0.43 MB PDF) [file pone.0003224.s007.pdf]

Supplementary Table S2

Proteins identified by two or more peptide spectral counts in non-diabetic and diabetic serum. P value: statistically difference of protein identified in non-diabetic serum and diabetic serum were test by the computing method of Localized Statistics of Protein Abundance Distribution (LSPAD).

IPI ID	Protein name	Peptide spectral count identified in diabetic serum	Peptide spectral count identified in non-diabetic serum	P value
IPI00022434	ALB protein	61457	47082	4.09E-91
IPI00514824	Complement component C4B	875	183	1.44E-80
IPI00555805	Complement component 4A	3896	2109	1.63E-69
IPI00032258	Complement C4 precursor	3846	2077	3.06E-69
IPI00453459	Complement Component 4B preproprotein	3933	2141	9.77E-69
IPI00418163	C4B1	3811	2077	2.48E-66
IPI00384697	ALB protein	47105	37323	6.64E-37
IPI00556148	Complement factor H	2732	1691	1.30E-30
IPI00465313	Alpha 2 macroglobulin variant	17016	13013	7.50E-26
IPI00478003	Alpha-2-macroglobulin precursor	17344	13335	3.06E-24
IPI00385264	Ig mu heavy chain disease protein	1614	880	4.42E-23
IPI00164623	Complement C3 precursor	9754	7267	8.64E-22
IPI00479708	Immunoglobulin heavy constant mu (IGHM)	2007	1204	1.02E-21
IPI00549273	Immunoglobulin heavy constant mu (IGHM)	1995	1190	3.09E-21
IPI00019943	Afamin precursor	553	221	1.57E-20
IPI00479169	65 kDa protein	1932	1181	2.35E-18
IPI00022488	Hemopexin precursor	1952	1268	2.99E-14
IPI00426051	Hypothetical protein DKFZp686C15213	5203	3835	6.18E-14
IPI00021727	C4b-binding protein alpha chain precursor	638	321	1.01E-13
IPI00478493	Haptoglobin precursor	4214	3100	7.28E-12

IPI00550991	Alpha-1-antichymotrypsin precursor	1088	628	2.99E-11
IPI00019591	Splice Isoform 1 of Complement factor B	1183	696	4.42E-11
IPI00021842	Apolipoprotein E precursor	394	181	3.28E-10
IPI00019399	Serum amyloid A-4 protein precursor	143	43	9.21E-10
IPI00021857	Apolipoprotein C-III precursor	144	49	3.87E-08
IPI00022392	Complement C1q subcomponent, A chain precursor	103	30	1.25E-07
IPI00021841	Apolipoprotein A-I precursor	4069	3112	2.14E-07
IPI00010865	Casein kinase II beta subunit	23	0	2.70E-07
IPI00396929	PREDICTED: similar to immunoglobulin M chain	165	68	1.55E-06
IPI00410714	Alpha 2 globin variant	433	244	3.33E-06
IPI00163446	The Human Immunoglobulin Heavy Diversity (IGHD)	134	53	4.03E-06
IPI00171834	Keratin, type I cytoskeletal 19	140	57	1.29E-05
IPI00399007	Hypothetical protein DKFZp686I04196	5114	4039	1.41E-05
IPI00003590	Quiescin Q6	15	0	4.53E-05
IPI00022389	Splice Isoform 1 of C-reactive protein	15	0	4.53E-05
IPI00015309	Keratin, type I cytoskeletal 12	89	33	7.63E-05
IPI00290077	Keratin, type I cytoskeletal 15	142	62	8.21E-05
IPI00217963	Keratin, type I cytoskeletal 16	223	117	0.000146
IPI00418422	The Human Immunoglobulin Heavy Diversity (IGHD)	69	23	0.000152
IPI00423461	Hypothetical protein DKFZp686C02220	828	548	0.000223
IPI00450768	Keratin, type I cytoskeletal 17	147	69	0.000275
IPI00011261	Complement component C8 gamma chain precursor	266	152	0.000392

IPI00556567	Ficolin-3 protein	80	33	0.00082
IPI00441196	Hypothetical protein	3090	2450	0.00095
IPI00386839	Amyloid lambda 6 light chain variable region SAR	180	98	0.00123
IPI00017601	Ceruloplasmin precursor	2260	1816	0.001477
IPI00383953	VH4 heavy chain variable region precursor	132	64	0.001483
IPI00009866	Keratin, type I cytoskeletal 13	107	52	0.001919
IPI00470798	Hypothetical protein DKFZp686E23209	4508	3647	0.002099
IPI00017530	Ficolin-2 precursor	9	0	0.002266
IPI00021854	Apolipoprotein A-II precursor	853	582	0.002359
IPI00004550	Hypothetical protein FLJ20261	96	45	0.002388
IPI00011252	Complement component C8 alpha chain precursor	81	36	0.002415
IPI00293898	Hepatocellular carcinoma associated protein TB6	19	4	0.002728
IPI00384444	Keratin, type I cytoskeletal 14	207	120	0.003123
IPI00021856	Apolipoprotein C-II precursor	32	11	0.004184
IPI00219806	S100 calcium-binding protein A7	8	0	0.004391
IPI00446354	Hypothetical protein FLJ41805	8	0	0.004391
IPI00479762	115 kDa protein	8	0	0.004391
IPI00022446	Platelet factor 4 precursor	82	39	0.00501
IPI00300725	Keratin, type II cytoskeletal 6A	158	90	0.005161
IPI00242956	Fc fragment of IgG binding protein	24	8	0.006549
IPI00384401	Myosin-reactive immunoglobulin kappa chain variable region	25	8	0.006595
IPI00293665	Keratin, type II cytoskeletal 6B	141	79	0.007064
IPI00299145	Keratin, type II cytoskeletal 6E	144	83	0.007904
IPI00383603	Anti-thyroglobulin light chain variable region	7	0	0.008538

IPI00452748	Serum amyloid A protein precursor	7	0	0.008538
IPI00021304	Keratin, type II cytoskeletal 2 epidermal	810	575	0.009876
IPI00384410	Myosin-reactive immunoglobulin heavy chain variable region	45	18	0.010539
IPI00478600	Ig kappa chain V-I region HK102 precursor	21	7	0.011151
IPI00161229	12 kDa protein	11	2	0.013088
IPI00174757	F-box/LRR-repeat protein 20	11	2	0.013088
IPI00382488	Ig heavy chain V-III region HIL	54	24	0.014584
IPI00241841	Keratin 6L	91	49	0.01646
IPI00292684	Nuclear factor of activated T-cells, cytoplasmic 4	20	7	0.016874
IPI00031423	Keratin, type I cuticular HA3-II	32	13	0.017334
IPI00216137	Synaptonemal complex protein 1	13	3	0.020644
IPI00009794	Calcium binding protein Cab45	8	1	0.021392
IPI00022445	Platelet basic protein precursor	39	17	0.021453
IPI00022295	Platelet factor 4 variant precursor	56	26	0.021747
IPI00006662	Apolipoprotein D precursor	112	66	0.029104
IPI00384952	Hypothetical protein DKFZp686K04218	1022	744	0.030479
IPI00029260	Monocyte differentiation antigen CD14 precursor	11	3	0.032456
IPI00016702	Rab6 GTPase activating protein, GAPCenA	5	0	0.032625
IPI00216298	Thioredoxin	5	0	0.032625
IPI00217996	Putative 4 repeat voltage-gated ion channel	5	0	0.032625
IPI00438286	Splice Isoform 1 of LAP2 protein	5	0	0.032625
IPI00550363	Transgelin 2	5	0	0.032625
IPI00552578	Amyloid protein A	5	0	0.032625
IPI00025426	Pregnancy zone protein precursor	2482	2049	0.034367

IPI00000425	Vacuolar proton translocating ATPase 116 kDa subunit a isoform 2	9	2	0.035907
IPI00385253	Ig kappa chain V-III region CLL precursor	7	1	0.037562
IPI00385683	Ig heavy chain V-III region GAR	7	1	0.037562
IPI00413524	22 kDa protein	7	1	0.037562
IPI00009867	Keratin, type II cytoskeletal 5	137	85	0.038995
IPI00020019	Adiponectin precursor	13	4	0.042833
IPI00382421	Ig lambda chain V-I region NEW	10	3	0.050744
IPI00426069	Hypothetical protein DKFZp686M24218	3137	2592	0.05408
IPI00419442	IGLV6-57 protein	26	13	0.054548
IPI00552678	MGC27165 protein	1742	1394	0.054945
IPI00022229	Apolipoprotein B-100 precursor	4876	4066	0.057645
IPI00022420	Plasma retinol-binding protein precursor	449	325	0.064063
IPI00001617	Transcription factor SOX-4	4	0	0.064148
IPI00004860	Arginyl-tRNA synthetase	4	0	0.064148
IPI00007244	Splice Isoform H17 of Myeloperoxidase precursor	4	0	0.064148
IPI00008052	KIAA1287 protein	4	0	0.064148
IPI00009355	Zinc finger CCCH type domain containing protein 1	4	0	0.064148
IPI00018149	Centrosome protein Cep290	4	0	0.064148
IPI00026889	Disabled homolog 1	4	0	0.064148
IPI00064429	Regeneration associated muscle protease, isoform a	4	0	0.064148
IPI00165049	PREDICTED: similar to Hnrpa1 protein	4	0	0.064148
IPI00170594	TranscripTion facTor ELYS	4	0	0.064148
IPI00294469	Ubiquinone biosynthesis protein COQ4 homolog	4	0	0.064148

IPI00302453	Ciliary dynein heavy chain 9	4	0	0.064148
IPI00555665	RNA helicase	4	0	0.064148
IPI00216691	Profilin-1	12	4	0.06496
IPI00247295	Splice Isoform 4 of Nesprin 1	6	1	0.065381
IPI00333068	Actin-related protein 2/3 complex subunit 1A	6	1	0.065381
IPI00383871	Mitochondrial uncoupling protein 4	6	1	0.065381
IPI00387100	Ig kappa chain V-I region Roy	6	1	0.065381
IPI00296803	Hypothetical protein MGC40368	19	9	0.065769
IPI00008556	Splice Isoform 1 of Coagulation factor XI precursor	24	12	0.066818
IPI00100787	Hypothetical protein DKFZp434P144	9	3	0.078294
IPI00384391	Myosin-reactive immunoglobulin heavy chain variable region	98	62	0.087287
IPI00296365	Centromeric protein E	14	6	0.089144
IPI00430844	Hypothetical protein	1109	846	0.089274
IPI00000874	Peroxiredoxin 1	7	2	0.094157
IPI00001611	Splice Isoform 1 of Insulin-like growth factor II precursor	7	2	0.094157
IPI00032311	Lipopolysaccharide-binding protein precursor	7	2	0.094157
IPI00215836	Splice Isoform 1 of Protocadherin gamma A2 precursor	7	2	0.094157
IPI00217972	Collagen XXVII proalpha 1 chain precursor	7	2	0.094157
IPI00387119	Ig kappa chain V-III region POM	7	2	0.094157
IPI00454724	PREDICTED: similar to Ig kappa chain V region (A2) - human	7	2	0.094157
IPI00446534	Hypothetical protein FLJ41981	1106	841	0.096291
IPI00297116	Splice Isoform 1 of Pleckstrin homology domain-containing protein family A member	11	4	0.096865

IPI00000265	Chromosome 10 open reading frame 38	15	7	0.100411
IPI00217872	Splice Isoform 2 of Phosphoglucomutase	26	14	0.102182
IPI00426056	Hypothetical protein DKFZp686L19235	1107	846	0.104755
IPI00007461	PREDICTED: KIAA0877 protein	12	5	0.111583
IPI00333234	12 kDa protein	12	5	0.111583
IPI00426070	Hypothetical protein DKFZp686M08189	1098	832	0.111703
IPI00387110	Ig kappa chain V-II region MIL	16	8	0.112242
IPI00004557	BNIP2 motif containing molecule at the carboxyl tarminal region 1	5	1	0.112458
IPI00012391	Splice Isoform Long of Adenomatous polyposis coli protein	5	1	0.112458
IPI00024880	CEGP1 protein	5	1	0.112458
IPI00025092	Myosin-binding protein C, slow-type	5	1	0.112458
IPI00028540	OTTHUMP00000061872	5	1	0.112458
IPI00218414	Carbonic anhydrase II	5	1	0.112458
IPI00299635	Baculoviral IAP repeat-containing protein 6	5	1	0.112458
IPI00398012	PREDICTED: hypothetical protein	5	1	0.112458
IPI00400935	Collagen alpha 1(XVI) chain precursor	5	1	0.112458
IPI00382500	Ig heavy chain V-III region GAL	66	40	0.119752
IPI00470652	Single-chain Fv	40	22	0.124177
IPI00001671	Tumor necrosis factor precursor	3	0	0.126646
IPI00001743	Hypothetical protein DKFZp686G2174	3	0	0.126646
IPI00003384	Splice Isoform 1 of Cadherin EGF LAG seven-pass G-type receptor 1 precursor	3	0	0.126646
IPI00003947	Ig lambda chain V-II region BUR	3	0	0.126646
IPI00005024	MYB binding protein 1a	3	0	0.126646
IPI00007227	HypotHetical protein LOC22864	3	0	0.126646
IPI00007834	Splice Isoform 1 of Ankyrin-2	3	0	0.126646

IPI00008091	KIAA1259 protein	3	0	0.126646
IPI00008531	CoREST protein	3	0	0.126646
IPI00009286	Splice Isoform 1 of Zinc finger protein HRX	3	0	0.126646
IPI00009524	Splice Isoform Alpha of Tripartite motif protein 10	3	0	0.126646
IPI00012011	Cofilin-1	3	0	0.126646
IPI00015346	Cadherin EGF LAG seven-pass G-type receptor 2 precursor	3	0	0.126646
IPI00016461	1-phosphatidylinositol-4,5-bisphosphate phosphodiesterase delta 1	3	0	0.126646
IPI00022204	Squamous cell carcinoma antigen 1	3	0	0.126646
IPI00022731	Apolipoprotein C-IV precursor	3	0	0.126646
IPI00028551	PREDICTED: similar to LPIN3	3	0	0.126646
IPI00029737	Splice Isoform Long of Long-chain-fatty-acid--CoA ligase 4	3	0	0.126646
IPI00031836	Developmentally regulated GTP-binding protein 1	3	0	0.126646
IPI00032541	Keratin, type II cuticular HB5	3	0	0.126646
IPI00036742	Hypothetical protein DKFZp686C1522	3	0	0.126646
IPI00043215	ImmunoglobulIn superfamIly, member 1 Isoform 1	3	0	0.126646
IPI00045423	Splice Isoform 7 of Partitioning-defective 3 homolog	3	0	0.126646
IPI00103597	Splice Isoform 1 of VPS10 domain-containing receptor SorCS1 precursor	3	0	0.126646
IPI00170934	LKB1 interacting protein	3	0	0.126646
IPI00217002	Hypothetical protein DKFZp781O0144	3	0	0.126646
IPI00290368	Histidine decarboxylase	3	0	0.126646

IPI00301263	CAD protein	3	0	0.126646
IPI00304934	Hypothetical protein FLJ23467	3	0	0.126646
IPI00334715	Splice Isoform 2 of Glucocorticoid receptor DNA binding factor 1	3	0	0.126646
IPI00376221	Erythrocyte membrane protein band 4.1 like	3	0	0.126646
IPI00376394	Putative sulfhydryl oxidase precursor	3	0	0.126646
IPI00382422	Ig lambda chain V-I region NEWM	3	0	0.126646
IPI00383530	Splice Isoform 2 of Rho-GTPase-activating protein 25	3	0	0.126646
IPI00384805	Peroxisomal targeting signal import receptor	3	0	0.126646
IPI00401122	PREDICTED: similar to bA110H4.2 (similar to membrane protein)	3	0	0.126646
IPI00401831	Pleckstrin homology domain containing, family H (with MyTH4 domain) member 1	3	0	0.126646
IPI00410334	Sulfate/anion exchanger	3	0	0.126646
IPI00433284	D-glucuronyl C5-epimerase	3	0	0.126646
IPI00435946	PP10752	3	0	0.126646
IPI00446280	Hypothetical protein FLJ42424	3	0	0.126646
IPI00550834	Hypothetical protein FLJ36288	3	0	0.126646
IPI00556645	Similar to Forkhead box protein K1	3	0	0.126646
IPI00028413	Inter-alpha	106	70	0.126981
IPI00059369	Splice Isoform 1 of Hypothetical protein KIAA0555	9	4	0.14028
IPI00296156	Chromosome 10 open reading frame 33	9	4	0.14028
IPI00154742	IGLC1 protein	2169	1860	0.144673
IPI00019713	KIAA0754 protein	6	2	0.148839
IPI00375836	Cancer associated nucleoprotein	6	2	0.148839

IPI00413194	Myosin-reactive immunoglobulin heavy chain variable region	6	2	0.148839
IPI00328609	Kallistatin precursor	15	8	0.149457
IPI00061977	MGC27165 protein	1449	1138	0.151311
IPI00104074	M130 antigen cytoplasmic variant 2	11	5	0.158055
IPI00328550	Thrombospondin-4 precursor	16	9	0.160239
IPI00032179	Antithrombin III variant	471	357	0.163437
IPI00456619	Ig lambda chain C regions	2128	1822	0.16889
IPI00026195	Ig kappa chain V-II region RPMI 6410 precursor	35	20	0.170094
IPI00021855	Apolipoprotein C-I precursor	102	69	0.176985
IPI00412608	25 kDa protein	2126	1825	0.177117
IPI00299307	Complement-activating component of Ra-reactive factor precursor	7	3	0.177249
IPI00299547	Lipocalin 2	7	3	0.177249
IPI00375793	PREDICTED: similar to RAN-binding protein 2-like 1 isoform 1	7	3	0.177249
IPI00020918	LAF-4 protein	13	7	0.18588
IPI00017303	DNA mismatch repair protein Msh2	4	1	0.190239
IPI00018320	Protocadherin 11	4	1	0.190239
IPI00027410	Platelet glycoprotein V precursor	4	1	0.190239
IPI00032064	Splice Isoform A of A-kinase anchor protein	4	1	0.190239
IPI00054598	PREDICTED: similar to family with sequence similarity 24, member B	4	1	0.190239
IPI00176740	Hypothetical protein FLJ40265	4	1	0.190239
IPI00261031	PREDICTED: similar to hephaestin	4	1	0.190239
IPI00296776	Hypothetical protein FLJ23518	4	1	0.190239
IPI00305545	Debranching enzyme homolog 1	4	1	0.190239

IPI00328995	PREDICTED: similar to Retinitis pigmentosa 9 protein (Pim-1 associated protein) (PAP-1)	4	1	0.190239
IPI00376181	PREDICTED: similar to RIKEN cDNA B230396O12	4	1	0.190239
IPI00377116	Zinc finger protein 445	4	1	0.190239
IPI00401649	PREDICTED: hypothetical protein	4	1	0.190239
IPI00411389	Zinc finger protein 79	4	1	0.190239
IPI00419908	GPR158-like 1 receptor	4	1	0.190239
IPI00456919	OTTHUMP00000061860	4	1	0.190239
IPI00479915	Obscurin	4	1	0.190239
IPI00295252	Splice Isoform 1 of Phosphatidylinositol 3,4,5-trisphosphate-dependent Rac exchanger 1 protein	21	12	0.19066
IPI00385252	Ig kappa chain V-III region GOL	200	144	0.192203
IPI00007199	Protein Z-dependent protease inhibitor precursor	14	8	0.197808
IPI00384407	Myosin-reactive immunoglobulin heavy chain variable region	69	46	0.199198
IPI00005439	Fetuin-B precursor	8	4	0.200169
IPI00018219	Transforming growth factor-beta induced protein IG-H3 precursor	8	4	0.200169
IPI00027350	Peroxiredoxin 2	22	14	0.203456
IPI00218816	Hemoglobin beta chain	573	444	0.205717
IPI00003939	Ig lambda chain V-I region NIG-64	15	9	0.20906
IPI00007221	Plasma serine protease inhibitor precursor	15	9	0.20906
IPI00026175	Ig kappa chain V-II region GM607 precursor	24	15	0.210649
IPI00479461	23 kDa protein	2121	1820	0.216559

IPI00387024	Ig kappa chain V-I region CAR	25	16	0.217832
IPI00032899	DEAD (Asp-Glu-Ala-Asp) box polypeptide	9	5	0.219152
IPI00294713	Splice Isoform 1 of Mannan-binding lectin serine protease 2 precursor	9	5	0.219152
IPI00387105	Ig kappa chain V-I region Mev	9	5	0.219152
IPI00022391	Serum amyloid P-component precursor	103	72	0.222899
IPI00022429	Alpha-1-acid glycoprotein 1 precursor	297	218	0.225015
IPI00018769	Thrombospondin-2 precursor	5	2	0.23015
IPI00019502	Myosin heavy chain, nonmuscle type A	5	2	0.23015
IPI00552874	V1-3 protein	5	2	0.23015
IPI00550162	Hypothetical protein	2120	1825	0.231042
IPI00024825	Megakaryocyte stimulating factor	21	14	0.250802
IPI00000192	Splice Isoform F of SON protein	2	0	0.251098
IPI00000733	WD-repeat protein 50	2	0	0.251098
IPI00001690	Cullin-7	2	0	0.251098
IPI00001780	Ubiquitin carboxyl-terminal hydrolase 16	2	0	0.251098
IPI00001919	Similar to Nucleotide binding protein 2	2	0	0.251098
IPI00003441	Hypothetical protein ORF9 precursor	2	0	0.251098
IPI00004364	Orphan nuclear receptor PAR2	2	0	0.251098
IPI00004450	Testes specific heterogenous nuclear ribonucleoprotein G-T	2	0	0.251098
IPI00004970	Down-regulated in metastasis protein	2	0	0.251098
IPI00005020	Splice Isoform 4 of Zinc finger protein 64, isoforms 3 and 4	2	0	0.251098
IPI00005589	16 kDa protein	2	0	0.251098
IPI00006035	KIAA0590 protein	2	0	0.251098
IPI00006854	Ras GTPase-activating protein nGAP	2	0	0.251098
IPI00007344	Embryonic lung protein	2	0	0.251098

IPI00007843	Myelin transcription factor 1-like	2	0	0.251098
IPI00009464	Splice Isoform 1 of Exosome component 10	2	0	0.251098
IPI00009736	Retinoic acid receptor RXR-gamma	2	0	0.251098
IPI00009753	Pellino protein homolog 2	2	0	0.251098
IPI00010807	Frizzled 8 precursor	2	0	0.251098
IPI00010948	Tripartite motif protein 26	2	0	0.251098
IPI00012462	CDA02	2	0	0.251098
IPI00012858	Splice Isoform 1 of Potassium voltage-gated channel subfamily KQT member 2	2	0	0.251098
IPI00012887	Cathepsin L precursor	2	0	0.251098
IPI00012912	Carnitine O-palmitoyltransferase II, mitochondrial precursor	2	0	0.251098
IPI00013218	Patatin-like phospholipase domain containing protein 4	2	0	0.251098
IPI00013773	Receptor-interacting serine/threonine-protein kinase 2	2	0	0.251098
IPI00013838	51C protein	2	0	0.251098
IPI00013860	3-hydroxyisobutyrate dehydrogenase, mitochondrial precursor	2	0	0.251098
IPI00014238	Lysyl-tRNA synthetase	2	0	0.251098
IPI00015135	Exostosin-like 3	2	0	0.251098
IPI00015695	Hypothetical protein FLJ23554	2	0	0.251098
IPI00016553	Splice Isoform 1 of Solute carrier organic anion transporter family, member 2B1	2	0	0.251098
IPI00017502	De-etiolateD 1	2	0	0.251098
IPI00018236	Ganglioside GM2 activator precursor	2	0	0.251098
IPI00018321	Splice Isoform 1 of Histone-lysine N-methyltransferase, H3 lysine-9 specific 4	2	0	0.251098

IPI00018953	Dipeptidyl peptidase 4	2	0	0.251098
IPI00019172	Splice Isoform 1 of SH3-containing GRB2-like protein 3	2	0	0.251098
IPI00019282	Melanoma antigen preferentially expressed in tumors	2	0	0.251098
IPI00020128	Hepatocellular carcinoma-associated antigen	2	0	0.251098
IPI00020294	MYH7B protein	2	0	0.251098
IPI00020884	Plexin A3 precursor	2	0	0.251098
IPI00021772	S-adenosylmethionine synthetase alpha and beta forms	2	0	0.251098
IPI00022470	Zinc finger protein 516	2	0	0.251098
IPI00023617	Zinc finger protein 197	2	0	0.251098
IPI00023807	Semaphorin 4D precursor	2	0	0.251098
IPI00024754	Splice Isoform 1 of G-protein coupled receptor 64 precursor	2	0	0.251098
IPI00025418	Collagen alpha 1(VII) chain precursor	2	0	0.251098
IPI00026216	Puromycin-sensitive aminopeptidase	2	0	0.251098
IPI00027983	Cytidine deaminase	2	0	0.251098
IPI00028130	GC-rich sequence DNA-binding factor	2	0	0.251098
IPI00028931	Desmoglein-2 precursor	2	0	0.251098
IPI00029372	Hypothetical protein DKFZp686I1868	2	0	0.251098
IPI00029403	Sorting nexin 4	2	0	0.251098
IPI00029446	Splice Isoform 1 of SET binding factor 1	2	0	0.251098
IPI00029498	Ropporin	2	0	0.251098
IPI00029728	TFIIH basal transcription factor complex helicase subunit	2	0	0.251098
IPI00029751	Splice Isoform 1 of Contactin 1 precursor	2	0	0.251098

IPI00032121	Splice Isoform 5 of Natural cytotoxicity triggering receptor 3 precursor	2	0	0.251098
IPI00032929	Transmembrane molecule with thrombospondin module precursor	2	0	0.251098
IPI00045864	Hypothetical protein FLJ14710	2	0	0.251098
IPI00060200	Aldose 1-epimerase	2	0	0.251098
IPI00064917	MGC27169 protein	2	0	0.251098
IPI00065256	Leucine-rich repeat-containing protein 2	2	0	0.251098
IPI00066817	WD-repeat protein 10	2	0	0.251098
IPI00069270	Splice Isoform 1 of Nuclear receptor coactivator 3	2	0	0.251098
IPI00073148	Splice Isoform 1 of Cell death activator	2	0	0.251098
IPI00088953	P87 phosphoinositide 3-kinase gamma adapter protein	2	0	0.251098
IPI00101923	KIAA1840 protein	2	0	0.251098
IPI00102407	Novel protein	2	0	0.251098
IPI00147878	20 kDa protein	2	0	0.251098
IPI00150282	28 kDa protein	2	0	0.251098
IPI00151888	Splice Isoform 1 of Dedicator of cytokinesis protein 9	2	0	0.251098
IPI00152143	Zinc finger protein 519	2	0	0.251098
IPI00152255	ADAM 32 precursor	2	0	0.251098
IPI00152295	LOC132671	2	0	0.251098
IPI00152542	Rabconnectin	2	0	0.251098
IPI00152899	Splice Isoform 1 of Voltage-dependent T-type calcium channel alpha-1I subunit	2	0	0.251098
IPI00154829	EEG1L	2	0	0.251098
IPI00155437	FLJ14154 protein	2	0	0.251098

IPI00160622	Splice Isoform 1 of Centrosomal protein 2	2	0	0.251098
IPI00165970	Splice Isoform 1 of DGCR8 protein	2	0	0.251098
IPI00165984	Splice Isoform 1 of WD-repeat protein 35	2	0	0.251098
IPI00166323	Hypothetical protein FLJ33811	2	0	0.251098
IPI00166863	HypotHetical protein LOC84902	2	0	0.251098
IPI00167087	COL3A1 protein	2	0	0.251098
IPI00167292	Hypothetical protein FLJ40424	2	0	0.251098
IPI00167672	Hypothetical protein FLJ37906	2	0	0.251098
IPI00168743	NFAT activation molecule 1 precursor	2	0	0.251098
IPI00168920	OTTHUMP00000058978	2	0	0.251098
IPI00170751	Tigger transposable element derived 5 variant	2	0	0.251098
IPI00173461	Junctophilin 1	2	0	0.251098
IPI00175092	Ring finger protein 149	2	0	0.251098
IPI00176377	PREDICTED: similar to Sorbitol dehydrogenase (L-iditol 2-dehydrogenase)	2	0	0.251098
IPI00179197	Similar to Protein kinase	2	0	0.251098
IPI00183535	Hypothetical protein FLJ10876	2	0	0.251098
IPI00184874	Adenosine deaminase ADAR2	2	0	0.251098
IPI00187011	PREDICTED: chromosome 19 open reading frame 7	2	0	0.251098
IPI00216429	Splice Isoform 5-HT4(F) of 5-hydroxytryptamine 4 receptor	2	0	0.251098
IPI00216800	Similar to phospholipase A2, group IVB	2	0	0.251098
IPI00217001	PREDICTED: chromosome 14 open reading frame 78	2	0	0.251098
IPI00217259	TEX14 protein	2	0	0.251098
IPI00217437	TTBK2 protein	2	0	0.251098

IPI00217732	Aryl hydrocarbon receptor nuclear translocator-like	2	0	0.251098
IPI00218524	Splice Isoform 2 of Piccolo protein	2	0	0.251098
IPI00219505	Splice Isoform 3 of Ubiquitin carboxyl-terminal hydrolase 15	2	0	0.251098
IPI00219529	Splice Isoform 2 of Dystrobrevin beta	2	0	0.251098
IPI00219685	Cell death-regulatory protein GRIM19	2	0	0.251098
IPI00219913	Ubiquitin carboxyl-terminal hydrolase 14	2	0	0.251098
IPI00233074	PREDICTED: melanoma antigen, family B, 5	2	0	0.251098
IPI00235051	PREDICTED: hypothetical protein	2	0	0.251098
IPI00235708	PREDICTED: similar to RIKEN cDNA 0610012A05	2	0	0.251098
IPI00241409	PREDICTED: hypothetical protein	2	0	0.251098
IPI00241802	PREDICTED: similar to hypothetical protein DKFZp434J0113	2	0	0.251098
IPI00248930	51 kDa protein	2	0	0.251098
IPI00289851	OTTHUMP00000022249	2	0	0.251098
IPI00290854	A-kinase anchor protein 3	2	0	0.251098
IPI00290856	Lymphatic endothelium-specific hyaluronan receptor LYVE-1	2	0	0.251098
IPI00292130	Dermatopontin precursor	2	0	0.251098
IPI00292326	Sperm-associated antigen 1	2	0	0.251098
IPI00293655	ATP-dependent helicase DDX1	2	0	0.251098
IPI00293659	Polycystic kidney disease 1-like isoform b	2	0	0.251098
IPI00295601	BM88 antigen	2	0	0.251098
IPI00296036	Eomesodermin homolog	2	0	0.251098
IPI00296114	Large neutral amino acids transporter small subunit 2	2	0	0.251098

IPI00296353	Hypothetical protein DKFZp762I167	2	0	0.251098
IPI00297860	G2/mitotic-specific cyclin F	2	0	0.251098
IPI00297880	Splice Isoform Long of Integrin beta-7 precursor	2	0	0.251098
IPI00300126	56 kDa protein	2	0	0.251098
IPI00300393	Hypothetical protein FLJ35936	2	0	0.251098
IPI00302320	Hypothetical protein	2	0	0.251098
IPI00303401	FLJ10874 protein	2	0	0.251098
IPI00304721	Splice Isoform 1 of Potential carboxypeptidase X precursor	2	0	0.251098
IPI00304903	Cornifin B	2	0	0.251098
IPI00305296	Hypothetical protein FLJ11773	2	0	0.251098
IPI00305360	Agmatinase, mitochondrial precursor	2	0	0.251098
IPI00305827	Fibronectin type III and SPRY domain containing 1	2	0	0.251098
IPI00328156	Amine oxidase [flavin-containing] B	2	0	0.251098
IPI00328350	Niban protein	2	0	0.251098
IPI00328905	185 kDa protein	2	0	0.251098
IPI00335044	Mitochondrial solute carrier protein	2	0	0.251098
IPI00335617	OTTHUMP00000042125	2	0	0.251098
IPI00374294	PREDICTED: hypothetical protein	2	0	0.251098
IPI00374907	PREDICTED: hypothetical protein	2	0	0.251098
IPI00375737	Hypothetical protein DKFZp686K091	2	0	0.251098
IPI00375907	Transducer of regulated cAMP response element-binding protein	2	0	0.251098
IPI00377122	KIAA1875 protein	2	0	0.251098
IPI00382439	Ig lambda chain V-IV region X	2	0	0.251098
IPI00382511	Hypothetical protein FLJ30520	2	0	0.251098

IPI00382989	DERP13	2	0	0.251098
IPI00383356	Alpha-1 type XIII collagen	2	0	0.251098
IPI00383526	Ligase-like protein	2	0	0.251098
IPI00384396	V4-2 protein	2	0	0.251098
IPI00385800	Sarcolemmal associated protein 1	2	0	0.251098
IPI00386940	Hypothetical protein FLJ11811	2	0	0.251098
IPI00387168	Splice Isoform 1 of Proprotein convertase subtilisin/kexin type 9 precursor	2	0	0.251098
IPI00394693	Shb-like adapter protein, Shf	2	0	0.251098
IPI00396658	Hypothetical protein FLJ32603	2	0	0.251098
IPI00397015	Novel protein	2	0	0.251098
IPI00397125	PREDICTED: hypothetical protein	2	0	0.251098
IPI00397491	PREDICTED: hypothetical protein	2	0	0.251098
IPI00398716	BA207C16.2	2	0	0.251098
IPI00399062	PREDICTED: similar to hypothetical protein	2	0	0.251098
IPI00399254	HIV-1 induced protein HIN-1 isoform 1	2	0	0.251098
IPI00401113	PREDICTED: similar to acetoacetyl-CoA synthetase	2	0	0.251098
IPI00401472	PREDICTED: hypothetical protein	2	0	0.251098
IPI00410355	Testis-specific BRDT protein	2	0	0.251098
IPI00411844	MSTP086	2	0	0.251098
IPI00414165	53 kDa protein	2	0	0.251098
IPI00414388	351 kDa protein	2	0	0.251098
IPI00418760	Hypothetical protein FLJ46362	2	0	0.251098
IPI00419527	FLJ43980 protein	2	0	0.251098
IPI00428401	Splice Isoform 1 of Methyl-CpG-binding domain protein 5	2	0	0.251098
IPI00445439	Hypothetical protein FLJ44095	2	0	0.251098

IPI00455388	PREDICTED: similar to zinc finger protein 91 (HPF7, HTF10)	2	0	0.251098
IPI00455902	PREDICTED: similar to extensin-like protein	2	0	0.251098
IPI00456560	PREDICTED: hypothetical protein	2	0	0.251098
IPI00456733	Novel protein	2	0	0.251098
IPI00457155	PREDICTED: similar to hypothetical protein DKFZp434I1020	2	0	0.251098
IPI00464973	PREDICTED: odz, odd Oz/ten-m homolog 4	2	0	0.251098
IPI00465061	FLJ10081 protein	2	0	0.251098
IPI00465123	PREDICTED: similar to myocyte nuclear	2	0	0.251098
IPI00465140	Hypothetical protein DKFZp686K0367	2	0	0.251098
IPI00470802	Hypothetical protein DKFZp781B0622	2	0	0.251098
IPI00470867	Hypothetical protein DKFZp686C0668	2	0	0.251098
IPI00470954	Hypothetical protein DKFZp686N1231	2	0	0.251098
IPI00477349	Hypothetical protein FLJ25919	2	0	0.251098
IPI00477585	39 kDa protein	2	0	0.251098
IPI00478375	12 kDa protein	2	0	0.251098
IPI00479575	107 kDa protein	2	0	0.251098
IPI00479691	33 kDa protein	2	0	0.251098
IPI00480010	PR-domain zinc finger protein 13	2	0	0.251098
IPI00550716	Hypothetical protein	2	0	0.251098
IPI00552561	Novel protein	2	0	0.251098
IPI00552566	HSPC004	2	0	0.251098
IPI00555942	Dopamine beta-hydroxylase	2	0	0.251098
IPI00294004	Vitamin K-dependent protein S precursor	164	119	0.254734
IPI00022918	Chromosome 9 open reading frame 28, isoform 2	22	15	0.256208
IPI00297333	PREDICTED: KIAA1171 protein	13	8	0.257204

IPI00179330	Ubiquitin and ribosomal protein S27a	6	3	0.258203
IPI00298738	DNA-directed RNA polymerase, mitochondrial precursor	6	3	0.258203
IPI00382442	Ig lambda chain V-V region DEL	6	3	0.258203
IPI00215983	Carbonic anhydrase I	14	9	0.267141
IPI00387117	Ig kappa chain V-III region Ti	191	141	0.271347
IPI00385985	Ig lambda chain V-III region LOI	105	74	0.272949
IPI00395595	DEAH (Asp-Glu-Ala-Asp/His) box polypeptide 57 isoform 1	7	4	0.279328
IPI00292950	Heparin cofactor II precursor	222	168	0.287472
IPI00328103	Type I inner root sheath specific keratin 25	96	69	0.291299
IPI00477644	25 kDa protein	1764	1478	0.292044
IPI00007852	Myosin-reactive immunoglobulin light chain variable region	20	14	0.304552
IPI00010195	Hypothetical protein FLJ12911	3	1	0.314143
IPI00018279	Collagen alpha 3(V) chain precursor	3	1	0.314143
IPI00018879	Alpha-L-iduronidase precursor	3	1	0.314143
IPI00020197	Alpha-N-acetylneuraminide alpha-2,8-sialyltransferase	3	1	0.314143
IPI00022919	Protein A-2	3	1	0.314143
IPI00024284	Basement membrane-specific heparan sulfate proteoglycan core protein precursor	3	1	0.314143
IPI00026698	KIAA0822 protein	3	1	0.314143
IPI00030880	Splice Isoform Flop of Glutamate receptor 1 precursor	3	1	0.314143
IPI00044369	ARFP2514	3	1	0.314143
IPI00045914	Msx2-interacting protein	3	1	0.314143
IPI00061009	Hypothetical protein FLJ31872	3	1	0.314143

IPI00062213	Hypothetical protein FLJ33334	3	1	0.314143
IPI00063523	PREDICTED: similar to Centromeric protein E (CENP-E protein)	3	1	0.314143
IPI00142538	Splice Isoform 1 of Probable helicase	3	1	0.314143
IPI00152865	PREDICTED: RUN and TBC1 domain containing 2	3	1	0.314143
IPI00166291	Sprouty-related, EVH1 domain containing protein 1	3	1	0.314143
IPI00216197	Splice Isoform C of Caspase-10 precursor	3	1	0.314143
IPI00221224	Aminopeptidase N	3	1	0.314143
IPI00292171	Chromobox homolog 2 isoform 1	3	1	0.314143
IPI00298057	Periplakin	3	1	0.314143
IPI00302592	FLJ00343 protein	3	1	0.314143
IPI00333143	Diacylglycerol kinase, alpha	3	1	0.314143
IPI00334254	Hypothetical protein FLJ40230	3	1	0.314143
IPI00382476	Ig heavy chain V-III region WEA	3	1	0.314143
IPI00382539	Ig heavy chain V-II region WAH	3	1	0.314143
IPI00385035	Neural cell adhesion molecule	3	1	0.314143
IPI00397204	PREDICTED: similar to Ig kappa chain precursor V region (orphon V108) - human (fragment)	3	1	0.314143
IPI00402655	PREDICTED: hypothetical protein	3	1	0.314143
IPI00409675	Splice Isoform 1 of Mortality factor 4-like protein 1	3	1	0.314143
IPI00418325	MGC17624 protein	3	1	0.314143
IPI00472263	Putative transcription factor-like nuclear regulator	3	1	0.314143

IPI00477911	55 kDa protein	3	1	0.314143
IPI00478344	19 kDa protein	3	1	0.314143
IPI00514320	Similar to Lamin A/C	3	1	0.314143
IPI00554752	cAMP-dependent protein kinase type II-beta regulatory subunit	3	1	0.314143
IPI00604736	Hematopoietic protein 1	3	1	0.314143
IPI00290078	Keratin, type II cytoskeletal 4	22	16	0.314735
IPI00387107	Ig kappa chain V-II region Cum	22	16	0.314735
IPI00047471	Hypothetical protein FLJ23984	11	7	0.319102
IPI00307729	ADAMTS-3 precursor	12	8	0.328025
IPI00387115	Ig kappa chain V-III region SIE	204	155	0.335266
IPI00430823	Hypothetical protein	1758	1476	0.342103
IPI00019576	Coagulation factor X precursor	14	10	0.343016
IPI00002232	Serine/threonine kinase TAO1	4	2	0.3458
IPI00022316	28S ribosomal protein S18b, mitochondrial precursor	4	2	0.3458
IPI00022890	Ig lambda chain V region 4A precursor	4	2	0.3458
IPI00175101	PREDICTED: similar to KIAA0445 protein	4	2	0.3458
IPI00339217	Ovochymase precursor	4	2	0.3458
IPI00395333	PREDICTED: similar to BMP-2 inducible protein kinase (BIKe) (HRIHFB2017)	4	2	0.3458
IPI00445800	Hypothetical protein FLJ43087	4	2	0.3458
IPI00455917	PREDICTED: similar to Collagen-binding protein 2 precursor (Colligin 2) (Rheumatoid arthritis related antigen RA-A47)	4	2	0.3458
IPI00552943	V1-11 protein	4	2	0.3458
IPI00604529	Hypothetical protein	4	2	0.3458

IPI00242446	PREDICTED: hypothetical protein	150	114	0.354876
IPI00553132	SNC73 protein	1663	1362	0.363926
IPI00007906	Myosin-reactive immunoglobulin heavy chain variable region	5	3	0.365668
IPI00009826	Carboxypeptidase B precursor	5	3	0.365668
IPI00030917	Growth factor receptor-bound protein 14	5	3	0.365668
IPI00060523	Hypothetical protein LOC116238	5	3	0.365668
IPI00183368	PI-3-kinase-related kinase SMG-1	5	3	0.365668
IPI00293451	TighT juncTion proTein 3 (zona occludens 3)	5	3	0.365668
IPI00387097	Ig kappa chain V-I region Lay	5	3	0.365668
IPI00431749	Keratin b20	20	15	0.368405
IPI00011255	Platelet glycoprotein Ib alpha chain precursor	6	4	0.379633
IPI00021364	Properdin precursor	6	4	0.379633
IPI00292690	Hypothetical protein PIK3CG	6	4	0.379633
IPI00552267	Homo sapiens This CDS feature is included to show the translation of the corresponding V_region. Presently translation qualifiers on V_region features are illegal.	6	4	0.379633
IPI00011268	RNA binding protein	25	18	0.380526
IPI00382499	Ig heavy chain V-III region JON	27	19	0.383464
IPI00386879	Hypothetical protein FLJ14473	1660	1370	0.384882
IPI00298888	Protocadherin 17	7	5	0.39015
IPI00328762	ABC A13	7	5	0.39015
IPI00328842	ARHGAP4 protein	7	5	0.39015
IPI00296608	Complement component C7 precursor	127	99	0.394384
IPI00007047	Calgranulin A	9	6	0.398386
IPI00387106	Ig kappa chain V-I region Ni	9	6	0.398386

IPI00023014	Von Willebrand factor precursor	153	117	0.403071
IPI00430806	Hypothetical protein	2071	1799	0.404321
IPI00454790	PREDICTED: hypothetical protein	10	7	0.404453
IPI00556624	Keratin 1	1493	1213	0.40805
IPI00004373	Mannose-binding protein C precursor	12	9	0.414116
IPI00030809	Gamma-G globin	61	45	0.422231
IPI00384404	Myosin-reactive immunoglobulin heavy chain variable region	15	12	0.42435
IPI00423460	Hypothetical protein DKFZp686G21220	1518	1238	0.431388
IPI00019359	Keratin 9	720	571	0.433109
IPI00290857	Keratin, type II cytoskeletal 3	20	16	0.434279
IPI00386158	Hypothetical protein	2066	1805	0.448628
IPI00450309	IGLC2 protein	2035	1766	0.454592
IPI00555945	IGLC2 protein	2045	1781	0.474101
IPI00292530	Inter-alpha-trypsin inhibitor heavy chain H1 precursor	779	623	0.489254
IPI00002466	Hypothetical protein LQFBS-1	2	2	0.5
IPI00003351	Extracellular matrix protein 1 precursor	23	19	0.5
IPI00003971	Splice Isoform RTN1-A of Reticulon 1	6	5	0.5
IPI00004566	Tyrosine-protein kinase ITK/TSK	6	6	0.5
IPI00005079	Mothers against decapentaplegic homolog 7	2	1	0.5
IPI00005661	Splice Isoform Gamma of Transcription factor-like protein 4	49	37	0.5
IPI00006091	Splice Isoform 4 of Dystrophin	2	1	0.5
IPI00006543	Complement factor H-related 5	14	12	0.5
IPI00007067	Golgi-associated plant pathogenesis-related protein 1	3	2	0.5
IPI00007219	Cytochrome P450 2C9	3	2	0.5

IPI00007411	A-kinase anchor protein 11	6	6	0.5
IPI00007884	Myosin-reactive immunoglobulin light chain variable region	15	13	0.5
IPI00008603	Actin, aortic smooth muscle	3	2	0.5
IPI00008787	Alpha-N-acetylglucosaminidase precursor	2	1	0.5
IPI00008868	Microtubule-associated protein 1B	2	1	0.5
IPI00009243	Splice Isoform B of Leptin receptor	2	1	0.5
IPI00010200	YTH domain containing 2	2	1	0.5
IPI00011180	Zinc finger and BTB domain containing protein 24	19	16	0.5
IPI00013397	KRAB box family protein	2	1	0.5
IPI00013439	Transcription factor jun-B	2	2	0.5
IPI00013444	Zinc finger protein 40	2	1	0.5
IPI00013881	Heterogeneous nuclear ribonucleoprotein H1	3	3	0.5
IPI00014829	Cadherin protein	2	1	0.5
IPI00015580	Transducer of Cdc42-dependent actin assembly-1	2	1	0.5
IPI00015593	Zinc finger protein 294	2	2	0.5
IPI00015785	Crumbs protein homolog 1 precursor	5	5	0.5
IPI00015793	Splice Isoform 1 of Telomerase-binding protein EST1A	2	1	0.5
IPI00018305	Insulin-like growth factor binding protein 3 precursor	2	2	0.5
IPI00019038	Lysozyme C precursor	10	9	0.5
IPI00019350	Dudulin 2, isoform b	6	6	0.5
IPI00019447	Conserved hypothetical protein	4	4	0.5
IPI00020356	Microtubule-associated protein 1A	2	1	0.5
IPI00020926	Homeobox protein Hox-A4	2	2	0.5

IPI00021033	Collagen alpha 1(III) chain precursor	2	1	0.5
IPI00021106	Endonuclease G like 1	2	1	0.5
IPI00022086	KIAA1325 protein	2	2	0.5
IPI00022333	Brain-specific angiogenesis inhibitor 1	2	1	0.5
IPI00022643	Tripartite motif protein 36	4	4	0.5
IPI00023339	CREB-binding protein	2	1	0.5
IPI00023728	Gamma-glutamyl hydrolase precursor	2	1	0.5
IPI00023756	Jumonji protein	2	1	0.5
IPI00024297	Zinc finger X-linked protein ZXDB	2	2	0.5
IPI00024382	CLIP-associating protein 2	2	1	0.5
IPI00024692	Transforming growth factor-beta-inducible early growth response protein 2	2	1	0.5
IPI00025753	Desmoglein-1 precursor	2	1	0.5
IPI00026087	Barrier-to-autointegration factor	2	1	0.5
IPI00026230	Heterogeneous nuclear ribonucleoprotein H'	13	12	0.5
IPI00027078	Carboxypeptidase D precursor	2	1	0.5
IPI00028348	Splice Isoform A of Collagen alpha 6(IV) chain precursor	2	1	0.5
IPI00029061	Selenoprotein P precursor	3	2	0.5
IPI00029768	Glutamate [NMDA] receptor subunit epsilon 1 precursor	2	2	0.5
IPI00029966	Hypothetical protein A-589H1.1	5	5	0.5
IPI00031721	KLHDC4 protein	2	1	0.5
IPI00032955	Zinc finger protein 313	2	2	0.5
IPI00043430	Hypothetical protein FLJ31400	3	2	0.5
IPI00045512	Hemicentin	2	2	0.5
IPI00061354	Splice Isoform 2 of Bromodomain adjacent to zinc finger domain 2B	2	2	0.5

IPI00063123	17 kDa protein	2	2	0.5
IPI00063780	Hypothetical protein	2	1	0.5
IPI00064667	Glutamate carboxypeptidase-like protein 2 precursor	2	1	0.5
IPI00064931	Splice Isoform 2 of E1A binding protein	2	2	0.5
IPI00065388	Novel protein	3	2	0.5
IPI00066333	Anion transporter/exchanger-8	5	4	0.5
IPI00074719	KIAA1792 protein	2	1	0.5
IPI00102193	Hypothetical protein FLJ25037	3	3	0.5
IPI00102820	AT2 receptor-interacting protein 1	2	2	0.5
IPI00103480	Membrane-bound phosphatidic acid-selective phospholipase A1	3	2	0.5
IPI00103536	Insulinoma-glucagonoma protein 20	10	8	0.5
IPI00103552	Ovarian cancer related tumor marker CA125	5	4	0.5
IPI00103655	Splice Isoform Long of Autism susceptibility gene 2 protein	2	1	0.5
IPI00107357	CLPTM1 protein	2	1	0.5
IPI00152189	Hypothetical protein	5	5	0.5
IPI00152344	Phosphatase, orphan 2	2	1	0.5
IPI00155729	Plexin B3 precursor	4	4	0.5
IPI00160348	Claspin	2	1	0.5
IPI00163956	PREDICTED: similar to family with sequence similarity 35, member A	2	1	0.5
IPI00166584	Soluble aminopeptidase P	3	3	0.5
IPI00166873	Chromosome 9 open reading frame 23	2	2	0.5
IPI00168608	Hypothetical protein LOC222967	3	2	0.5
IPI00168884	Renin receptor precursor	2	2	0.5
IPI00169288	Aldehyde dehydrogenase 1A2, isoform 2	2	1	0.5

IPI00170593	Cyclin-dependent kinase (CDC2-like) 11	2	1	0.5
IPI00174756	Ankyrin repeat domain protein 21	2	2	0.5
IPI00174775	Keratin 6 irs3	10	8	0.5
IPI00179016	HypotHetical protein LOC9739	4	3	0.5
IPI00179687	Soluble interleukin-5 receptor precursor	2	1	0.5
IPI00180305	Retinoblastoma-associated factor 600	2	2	0.5
IPI00181697	ARAP1	2	1	0.5
IPI00215899	Splice Isoform 2 of Sushi repeat-containing protein SRPX precursor	2	2	0.5
IPI00215995	Splice Isoform Alpha-3A of Integrin alpha-3 precursor	2	2	0.5
IPI00216070	Myosin light chain 1, skeletal muscle isoform	2	2	0.5
IPI00216560	Cadherin related 23	3	2	0.5
IPI00217017	Hypothetical protein FLJ39885	6	5	0.5
IPI00217051	Steerin3 protein	2	1	0.5
IPI00217052	Steerin2 protein	2	2	0.5
IPI00217537	Splice Isoform 1 of Putative Polycomb group protein ASXL1	3	2	0.5
IPI00218725	Laminin, alpha 2	4	3	0.5
IPI00218823	Splice Isoform 1 of Myeloid/lymphoid or mixed-lineage leukemia protein 4	2	2	0.5
IPI00218845	Nitric-oxide synthase, endothelial	4	4	0.5
IPI00219217	L-lactate dehydrogenase B chain	5	4	0.5
IPI00219430	Thyroid hormone receptor-associated protein complex 100 kDa component	2	1	0.5
IPI00219613	Metalloprotease 1	4	3	0.5
IPI00220617	Similar to 6-phosphofructokinase, liver type	4	4	0.5
IPI00220736	SialidaSe 3	2	1	0.5

IPI00220753	Splice Isoform Short of Early growth response protein 2	2	1	0.5
IPI00221090	Zinc finger homeobox 2	5	5	0.5
IPI00221234	Aldehyde dehydrogenase family 7 member	2	1	0.5
IPI00232047	PREDICTED: similar to protein tyrosine phosphatase, receptor type, Q isoform 1 precursor	4	3	0.5
IPI00240432	Splice Isoform A of Potential phospholipid-transporting ATPase VB	4	4	0.5
IPI00241676	FAT3	2	1	0.5
IPI00243338	Keratin 23 isoform b	2	1	0.5
IPI00243457	148 kDa protein	2	1	0.5
IPI00255301	OTTHUMP00000063615	2	2	0.5
IPI00259687	Adenylate cyclase, type I	8	7	0.5
IPI00291815	FBF1 protein	2	1	0.5
IPI00291867	Complement factor I precursor	126	101	0.5
IPI00292181	Mitogen-activated protein kinase kinase kinase 12	3	2	0.5
IPI00292393	Sodium channel protein type IV alpha subunit	2	1	0.5
IPI00292532	Antibacterial protein FALL-39 precursor	2	2	0.5
IPI00292836	OTTHUMP00000021741	3	2	0.5
IPI00293080	Novel protein	2	1	0.5
IPI00294008	ZW10 interactor	2	1	0.5
IPI00294891	Proliferating-cell nucleolar antigen p120	2	1	0.5
IPI00295376	Solute carrier organic anion transporter family, member 1B1	3	2	0.5
IPI00295400	Tryptophanyl-tRNA synthetase	3	2	0.5

IPI00296099	Thrombospondin 1 precursor	114	92	0.5
IPI00296561	Fibroblast growth factor receptor-like 1	2	2	0.5
IPI00296840	DNA polymerase iota	2	1	0.5
IPI00297550	Coagulation factor XIII A chain precursor	2	1	0.5
IPI00298964	Hypothetical protein FLJ20166	3	2	0.5
IPI00299059	Neural cell adhesion molecule	10	8	0.5
IPI00302835	AT rich interactive domain 1B (SWI1-like) isoform 3	2	1	0.5
IPI00303335	Nebulin	2	2	0.5
IPI00306929	Splice Isoform 1 of Myosin XVIIIB	3	3	0.5
IPI00328125	Superkiller viralicidic activity 2-like homolog	2	1	0.5
IPI00329213	SH2 containing inositol-5-phosphatase	2	1	0.5
IPI00329482	OTTHUMP00000040423	3	2	0.5
IPI00337602	Zinc finger protein 326	2	1	0.5
IPI00373872	Polycystic kidney disease 1-like 2	3	2	0.5
IPI00374082	C21orf258 protein	2	1	0.5
IPI00375559	Splice Isoform 1 of Otoferlin	3	2	0.5
IPI00376976	Splice Isoform 1 of 130-kDa phosphatidylinositol 4,5-biphosphate-dependent ARF1 GTPase- activating protein	2	1	0.5
IPI00377041	CUB and Sushi multiple domains 3 isoform 1	2	2	0.5
IPI00377097	PREDICTED: similar to bA110H4.2 (similar to membrane protein)	2	1	0.5
IPI00382481	Ig heavy chain V-III region BUT	7	6	0.5
IPI00382682	Putative matrix cell adhesion molecule-3	3	3	0.5
IPI00384746	Copine-8	3	3	0.5

IPI00386131	Ig kappa chain V-III region IARC/BL41 precursor	13	11	0.5
IPI00387022	Ig kappa chain V-I region AG	3	2	0.5
IPI00387101	Ig kappa chain V-I region Scw	4	3	0.5
IPI00387111	PREDICTED: similar to Ig kappa chain	22	18	0.5
IPI00394855	Hypothetical protein FLJ44112	2	2	0.5
IPI00394958	Splice Isoform 1 of Zona pellucida binding protein 2 precursor	4	4	0.5
IPI00395488	Vasorin	12	10	0.5
IPI00396145	FYVE finger-containing phosphoinositide	2	2	0.5
IPI00396421	Hypothetical protein KIAA0776	4	4	0.5
IPI00396470	46 kDa protein	3	2	0.5
IPI00397147	PREDICTED: hypothetical protein	2	1	0.5
IPI00397163	PREDICTED: hypothetical protein	2	1	0.5
IPI00397299	PREDICTED: hypothetical protein	2	1	0.5
IPI00398007	UbiqUitin specific protease 40	2	1	0.5
IPI00398020	PREDICTED: odz, odd Oz/ten-m homolog 3	2	2	0.5
IPI00399252	JADE1L protein	6	6	0.5
IPI00400817	PREDICTED: similar to dystrophin-like	3	2	0.5
IPI00401773	Vacuolar protein sorting 37C	2	1	0.5
IPI00410516	Hypothetical protein FLJ45244	3	3	0.5
IPI00412307	Titin	3	3	0.5
IPI00412647	Death receptor interacting protein	2	1	0.5
IPI00414101	Splice Isoform 2 of DNA topoisomerase II, alpha isozyme	2	1	0.5
IPI00418411	Keratin 8 variant	20	17	0.5
IPI00418630	DNA polymerase sigma	2	1	0.5
IPI00419509	Novel protein	2	1	0.5

IPI00430472	Splice Isoform 1 of Activating signal cointegrator 1 complex subunit 3	2	1	0.5
IPI00440727	Splice Isoform 1 of Bromodomain-containing protein 4	2	1	0.5
IPI00443665	Hypothetical protein FLJ46792	2	1	0.5
IPI00455117	PREDICTED: hypothetical protein	2	1	0.5
IPI00455280	PREDICTED: similar to carbonic anhydrase VA, mitochondrial precursor	2	2	0.5
IPI00457067	PREDICTED: similar to scaffold attachment factor B	2	1	0.5
IPI00465065	Cytochrome P450 19A1	2	1	0.5
IPI00465082	Interleukin 31RA splice variant x3	2	1	0.5
IPI00470653	Single-chain Fv	6	6	0.5
IPI00472901	Chromodomain heliCase DNA binding	2	1	0.5
IPI00477354	73 kDa protein	9	7	0.5
IPI00477405	Lung adenoma susceptibility 1-like protein	8	6	0.5
IPI00479192	58 kDa protein	2	1	0.5
IPI00514358	PREDICTED: similar to TPTE and PTEN homologous inositol lipid phosphatase isoform alpha	2	1	0.5
IPI00514516	OTTHUMP00000015995	3	3	0.5
IPI00514616	OTTHUMP00000031364	3	2	0.5
IPI00552195	IGLV2-14 protein	1652	1366	0.5
IPI00553215	V1-5 protein	5	5	0.5
IPI00556128	Hypothetical protein PREDICTED: hypothetical protein FLJ34222 variant	3	2	0.5
IPI00556386	Insulin receptor substrate like protein	2	1	0.5

IPI00027507	Complement factor H-related protein 3 precursor	81	65	0.533312
IPI00430842	IGHA1 protein	1628	1349	0.544133
IPI00385555	Ig kappa chain V-I region BAN	30	24	0.554113
IPI00472226	Ig alpha-1 chain C region	1627	1350	0.558743
IPI00030205	Ig kappa chain V-III region HAH precursor	191	155	0.56455
IPI00550584	Ig alpha-1 chain C region	1629	1350	0.566008
IPI00021817	Vitamin K-dependent protein C precursor	18	17	0.567247
IPI00387026	Ig kappa chain V-I region EU	18	17	0.567247
IPI00053535	PREDICTED: similar to ENSANGP00000013733	16	15	0.571422
IPI00022895	Alpha-1B-glycoprotein precursor	762	623	0.575199
IPI00003111	Ig kappa chain V-I region AU	9	9	0.59177
IPI00007899	Single chain Fv	9	9	0.59177
IPI00032293	Cystatin C precursor	8	8	0.59639
IPI00103481	Keratin protein K6irs	8	8	0.59639
IPI00398220	Myosin-reactive immunoglobulin light chain variable region	8	8	0.59639
IPI00387113	Ig kappa chain V-III region B6	7	7	0.601655
IPI00470882	Hypothetical protein DKFZp781D2453	7	7	0.601655
IPI00216773	ALB protein	37818	32674	0.607138
IPI00419453	Ig kappa chain V-III region VG precursor	67	55	0.608016
IPI00012551	Splice Isoform 3 of Protocadherin gamma A11 precursor	6	7	0.608866
IPI00013944	Splice Isoform C2 of Heterogeneous nuclear ribonucleoproteins C1/C2	6	7	0.608866
IPI00293251	Splice Isoform 6 of Bullous pemphigoid antigen 1, isoforms 6/9/10	6	7	0.608866

IPI00386576	Ig lambda chain V-IV region MOL	6	7	0.608866
IPI00032328	Splice Isoform HMW of Kininogen precursor	263	215	0.60901
IPI00011832	Secreted phosphoprotein 24 precursor	5	6	0.617756
IPI00024661	Protein transport protein Sec24C	5	6	0.617756
IPI00026337	Splice Isoform 1 of Ran-binding protein 3	5	6	0.617756
IPI00329775	Splice Isoform 1 of Carboxypeptidase B2 precursor	5	6	0.617756
IPI00333126	Hypothetical protein DKFZp761L1518	5	6	0.617756
IPI00399193	PREDICTED: similar to KIAA1501 protein	5	6	0.617756
IPI00477452	10 kDa protein	5	6	0.617756
IPI00382497	Ig heavy chain V-III region TUR	190	157	0.62723
IPI00013933	Splice Isoform DPI of Desmoplakin	4	5	0.629027
IPI00029658	Splice Isoform 1 of EGF-containing fibulin-like extracellular matrix protein 1 precursor	4	5	0.629027
IPI00398916	PREDICTED: similar to RIKEN cDNA 3830422K02	4	5	0.629027
IPI00555818	184 kDa protein	4	5	0.629027
IPI00449920	Hypothetical protein FLJ90170	1634	1361	0.629886
IPI00218413	Biotinidase precursor	16	16	0.638498
IPI00003369	LIM domains containing protein 1	3	4	0.643995
IPI00009910	Toll-like receptor 8 precursor	3	4	0.643995
IPI00014843	OTTHUMP00000039401	3	4	0.643995
IPI00022250	Lymphocyte antigen 96 precursor	3	4	0.643995
IPI00022733	Splice Isoform 1 of Phospholipid transfer protein precursor	3	4	0.643995
IPI00025276	Splice Isoform XB of Tenascin-X precursor	3	4	0.643995
IPI00027744	Splice Isoform 1 of Mineralocorticoid	3	4	0.643995
IPI00152011	PTPL1-associated RhoGAP	3	4	0.643995

IPI00154969	LOC201175 protein	3	4	0.643995
IPI00185659	Aaa-protein	3	4	0.643995
IPI00298971	Vitronectin precursor	247	205	0.647745
IPI00382478	Ig heavy chain V-III region TIL	232	194	0.651945
IPI00008558	Plasma kallikrein precursor	122	104	0.656116
IPI00375910	Type I inner root sheath specific keratin 25	52	44	0.659925
IPI00030739	Apolipoprotein M	28	25	0.660809
IPI00215894	Splice Isoform LMW of Kininogen precursor	263	216	0.66083
IPI00473011	Hemoglobin delta chain	373	311	0.664097
IPI00004859	Bloom's syndrome protein	2	3	0.665289
IPI00009815	Hypothetical protein	2	3	0.665289
IPI00011694	Trypsin I precursor	2	3	0.665289
IPI00021812	Neuroblast differentiation associated protein AHNAK	2	3	0.665289
IPI00029819	Neurogenic locus notch homolog protein 3 precursor	2	3	0.665289
IPI00060474	Hypothetical protein MGC20806	2	3	0.665289
IPI00073772	Fructose-1,6-bisphosphatase	2	3	0.665289
IPI00160479	Hypothetical protein DKFZp313B2333	2	3	0.665289
IPI00179694	Klotho beta like protein	2	3	0.665289
IPI00185362	Neural cell adhesion molecule 1	2	3	0.665289
IPI00218795	L-selectin precursor	2	3	0.665289
IPI00297084	Dolichyl-diphosphooligosaccharide--protein glycosyltransferase 48 kDa subunit precursor	2	3	0.665289
IPI00303343	Serine arginine-rich pre-mRNA splicing factor SR-A1	2	3	0.665289
IPI00328793	Sterol regulatory element binding protein-2	2	3	0.665289
IPI00375676	Ferritin light chain	2	3	0.665289

IPI00382426	Ig lambda chain V-II region TRO	2	3	0.665289
IPI00418262	Fructose-bisphosphate aldolase C	2	3	0.665289
IPI00470896	Hypothetical protein DKFZp781H1112	2	3	0.665289
IPI00479981	12 kDa protein	2	3	0.665289
IPI00552854	Hypothetical protein	1604	1344	0.672068
IPI00002773	Splice Isoform 2 of Tyrosine-protein kinase JAK3	9	10	0.674943
IPI00022394	Complement C1q subcomponent, C chain precursor	125	107	0.678366
IPI00456772	Hypothetical protein	1667	1426	0.680994
IPI00019580	Plasminogen precursor	1105	919	0.681003
IPI00005721	Neutrophil defensin 1 precursor	8	9	0.682973
IPI00550232	CMYA3	8	9	0.682973
IPI00385507	V1-16 protein	19	19	0.685262
IPI00186903	Splice Isoform 2 of Apolipoprotein-L1 precursor	56	48	0.689047
IPI00027547	Dermcidin precursor	7	8	0.692005
IPI00007240	Coagulation factor XIII B chain precursor	76	65	0.695261
IPI00166866	MGC27165 protein	1638	1377	0.696345
IPI00473015	Similar to SNC66 protein	1501	1260	0.697767
IPI00000897	Potential helicase with zinc-finger domain	1	2	0.699219
IPI00002260	Hypothetical protein FLJ11715	1	2	0.699219
IPI00005635	Tubulin tyrosine ligase-like protein 4	1	2	0.699219
IPI00005918	Alpha-methylacyl-CoA racemase	1	2	0.699219
IPI00008225	Gamma-aminobutyric-acid receptor alpha-4 subunit precursor	1	2	0.699219
IPI00008456	Splice Isoform HSF4B of Heat shock factor protein 4	1	2	0.699219

IPI00012269	Multimerin 1 precursor	1	2	0.699219
IPI00013508	Alpha-actinin 1	1	2	0.699219
IPI00013988	Rho-GTPase-activating protein 5	1	2	0.699219
IPI00018370	Splice Isoform 2 of Supervillin	1	2	0.699219
IPI00020416	Tripeptidyl peptidase II	1	2	0.699219
IPI00022264	Zinc finger protein 297B	1	2	0.699219
IPI00027174	Splice Isoform 1 of Fibroblast growth factor receptor 3 precursor	1	2	0.699219
IPI00027487	Creatine kinase, M chain	1	2	0.699219
IPI00028031	Splice Isoform 1 of Acyl-CoA dehydrogenase, very-long-chain specific, mitochondrial precursor	1	2	0.699219
IPI00028957	Splice Isoform 2 of Ubiquitin conjugation factor E4 A	1	2	0.699219
IPI00029700	Splice Isoform Long of Down syndrome cell adhesion molecule precursor	1	2	0.699219
IPI00031008	Splice Isoform 1 of Tenascin precursor	1	2	0.699219
IPI00037283	Splice Isoform 5 of Dynamin 1-like protein	1	2	0.699219
IPI00064741	Leishmanolysin-like peptidase, variant 1	1	2	0.699219
IPI00064745	Splice Isoform 1 of Phosphatase and actin regulator 3	1	2	0.699219
IPI00074715	PREDICTED: similar to homeobox protein NKX2-6	1	2	0.699219
IPI00100867	Transcription initiation factor TFIID 210 kDa subunit	1	2	0.699219
IPI00102677	Splice Isoform 1 of Dual specificity testis-specific protein kinase 2	1	2	0.699219

IPI00103182	FKBP6-like	1	2	0.699219
IPI00105532	LLGL protein	1	2	0.699219
IPI00141118	EPC2 protein	1	2	0.699219
IPI00165086	Zinc finger protein 37a	1	2	0.699219
IPI00166184	Hypothetical protein FLJ40584	1	2	0.699219
IPI00168554	Sulfiredoxin	1	2	0.699219
IPI00171636	Neuron navigator 1	1	2	0.699219
IPI00175202	Zinc finger protein 433	1	2	0.699219
IPI00175295	PREDICTED: DKFZP586J0619 protein	1	2	0.699219
IPI00184546	Hypothetical protein MGC17943	1	2	0.699219
IPI00216815	Splice Isoform 2 of DNA topoisomerase III beta-1	1	2	0.699219
IPI00217690	Hypothetical protein MGC42105	1	2	0.699219
IPI00218539	Splice Isoform B of Collagen alpha 1(XI) chain precursor	1	2	0.699219
IPI00248359	PREDICTED: similar to POTE2A	1	2	0.699219
IPI00299111	Testicular soluble adenylyl cyclase	1	2	0.699219
IPI00299524	Condensin subunit 1	1	2	0.699219
IPI00306532	PREDICTED: similar to KIAA1783 protein	1	2	0.699219
IPI00335509	Dihydropyrimidinase related protein-5	1	2	0.699219
IPI00337315	Retinoblastoma binding protein 6 isoform 1	1	2	0.699219
IPI00337657	Nephrocystin 3	1	2	0.699219
IPI00375638	KIAA0685 gene product	1	2	0.699219
IPI00382490	Ig heavy chain V-III region BUR	1	2	0.699219
IPI00383775	199G4 protein	1	2	0.699219
IPI00387096	Ig kappa chain V-I region Kue	1	2	0.699219
IPI00396627	Splice Isoform 1 of Zinc phosphodiesterase ELAC protein 2	1	2	0.699219

IPI00397904	Nuclear pore complex protein Nup93	1	2	0.699219
IPI00410060	Hepatocellular carcinoma-associated antigen	1	2	0.699219
IPI00410150	Usher syndrome 2A isoform B	1	2	0.699219
IPI00414095	Eukaryotic translation initiation factor 4E nuclear import factor 1	1	2	0.699219
IPI00432655	PYST9371	1	2	0.699219
IPI00444845	Hypothetical protein FLJ45122	1	2	0.699219
IPI00449923	Splice Isoform 1 of Retinoic acid induced protein 1	1	2	0.699219
IPI00465430	G22P1 protein	1	2	0.699219
IPI00472882	HLA class I histocompatibility antigen, A-68 alpha chain precursor	1	2	0.699219
IPI00477279	21 kDa protein	1	2	0.699219
IPI00477880	Nebulin-related anchoring protein	1	2	0.699219
IPI00479883	12 kDa protein	1	2	0.699219
IPI00513917	Sorbin and SH3 domain containing 1	1	2	0.699219
IPI00514729	Dehydrodolichyl diphosphate synthase	1	2	0.699219
IPI00552701	Discs, large homolog 3	1	2	0.699219
IPI00384931	Hypothetical protein	1971	1740	0.706917
IPI00514982	13 kDa protein	26	24	0.714682
IPI00296165	Complement C1r subcomponent precursor	289	239	0.715797
IPI00027462	Calgranulin B	5	7	0.717561
IPI00168218	Hypothetical protein FLJ33718	21	21	0.732695
IPI00220594	Splice Isoform 4 of Intersectin 1	21	21	0.732695
IPI00029699	Ribonuclease 4 precursor	4	6	0.735107
IPI00165319	PREDICTED: FLJ46675 protein	19	20	0.739679
IPI00028030	Cartilage oligomeric matrix protein precursor	16	18	0.753625

IPI00007893	Myosin-reactive immunoglobulin heavy chain variable region	8	10	0.757217
IPI00003469	Ig kappa chain V-I region WEA	3	5	0.757258
IPI00374371	Hypothetical protein FLJ40392	3	5	0.757258
IPI00454729	PREDICTED: similar to putative scaffolding protein POSH	3	5	0.757258
IPI00456373	Splice Isoform 2 of Homeobox protein Meis3	3	5	0.757258
IPI00465264	NOD9	3	5	0.757258
IPI00218746	Complement Component 1, q subComponent, beta polypeptide preCursor	109	95	0.759917
IPI00553170	IGLV3-21 protein	1655	1427	0.760221
IPI00398992	Hypothetical protein DKFZp686N17164	15	17	0.761325
IPI00387095	Ig kappa chain V-I region Ka	7	9	0.768331
IPI00029193	Hepatocyte growth factor activator precursor	22	22	0.774474
IPI00553177	Alpha-1-antitrypsin precursor	5587	4890	0.778519
IPI00024138	Ig kappa chain V-III region VH precursor	12	15	0.779538
IPI00017746	BRCA1-associated RING domain protein 1	2	4	0.786484
IPI00030466	PREDICTED: putative ankyrin-repeat containing protein	2	4	0.786484
IPI00040638	Leukocyte immunogLobuLin-Like receptor, subfamiLy A, member 5	2	4	0.786484
IPI00299608	Splice Isoform 1 of 26S proteasome non-ATPase regulatory subunit 1	2	4	0.786484
IPI00455528	PREDICTED: similar to hypothetical protein	2	4	0.786484
IPI00294395	Complement component C8 beta chain precursor	123	109	0.786619
IPI00376379	Keratin 1B	82	75	0.789785
IPI00385143	Microfibrillar protein 2	28	27	0.791562

IPI00003470	Ig kappa chain V-I region Wes	5	8	0.797389
IPI00008554	Angiogenin precursor	5	8	0.797389
IPI00384398	Myosin-reactive immunoglobulin light chain variable region	5	8	0.797389
IPI00013438	Immunoglobulin lambda-like polypeptide 1 precursor	10	13	0.797663
IPI00299778	Serum paraoxonase/lactonase 3	10	13	0.797663
IPI00006154	Splice Isoform Long of Complement factor H-related protein 2 precursor	44	42	0.808404
IPI00020091	Alpha-1-acid glycoprotein 2 precursor	156	138	0.81122
IPI00023217	Splice Isoform 1 of Ryanodine receptor 2	4	7	0.816866
IPI00448985	Hypothetical protein	4622	4067	0.826145
IPI00550640	IGHG4 protein	4893	4303	0.827524
IPI00001790	Hypothetical protein FLJ12906	1	3	0.827578
IPI00001952	Probable endonuclease KIAA0830 precursor	1	3	0.827578
IPI00002335	Huntingtin	1	3	0.827578
IPI00006931	CGI-72 protein	1	3	0.827578
IPI00006987	ATP-dependent RNA helicase DDX24	1	3	0.827578
IPI00007868	Myosin-reactive immunoglobulin light chain variable region	1	3	0.827578
IPI00017538	Splice Isoform 2 of Separin	1	3	0.827578
IPI00018177	Hypothetical protein FLJ10408	1	3	0.827578
IPI00020153	Bassoon protein	1	3	0.827578
IPI00024317	Splice Isoform Long of Glutaryl-CoA dehydrogenase, mitochondrial precursor	1	3	0.827578
IPI00024887	Bone morphogenetic protein 6 precursor	1	3	0.827578
IPI00026108	Cytosolic phospholipase A2	1	3	0.827578
IPI00028307	Hypothetical protein FLJ22346	1	3	0.827578

IPI00094740	Splice Isoform 1 of RING finger protein 31	1	3	0.827578
IPI00171525	Sentrin-specific protease 3	1	3	0.827578
IPI00178352	Splice Isoform 1 of Filamin C	1	3	0.827578
IPI00186460	AlphA 1 type II collAgen isoform 2, preproprotein	1	3	0.827578
IPI00217804	Hypothetical protein FLJ10260	1	3	0.827578
IPI00219299	Talin 2	1	3	0.827578
IPI00298301	Myosin heavy chain, fast skeletal muscle, embryonic	1	3	0.827578
IPI00299512	Splice Isoform 2 of Neurofibromin	1	3	0.827578
IPI00375220	Splice Isoform 1 of Nance-Horan syndrome protein	1	3	0.827578
IPI00376251	PREDICTED: similar to Solute carrier family 2, facilitated glucose transporter, member 3 (Glucose transporter type 3, brain)	1	3	0.827578
IPI00377006	Sodium channel protein type V alpha subunit	1	3	0.827578
IPI00385631	Zinc finger, ZZ-type with EF hand domain 1	1	3	0.827578
IPI00402580	PREDICTED: hypothetical protein	1	3	0.827578
IPI00454961	PREDICTED: KIAA0415 gene product	1	3	0.827578
IPI00457080	PREDICTED: similar to submaxillary	1	3	0.827578
IPI00555887	Ring fingeR pRotein 31	1	3	0.827578
IPI00063408	Dehydrogenase E1 and transketolase domain containing protein 1	7	10	0.830072
IPI00023673	Galectin-3 binding protein precursor	35	34	0.835081
IPI00549747	Hypothetical protein	4623	4071	0.836924
IPI00384400	Myosin-reactive immunoglobulin heavy chain variable region	66	61	0.837601

IPI00166612	Cardiomyopathy assoCiated 5	3	6	0.840168
IPI00328894	Hypothetical protein	3	6	0.840168
IPI00292946	Thyroxine-binding globulin precursor	16	20	0.842511
IPI00043069	Breast cancer antigen NY-BR-1	6	9	0.842805
IPI00299435	Apolipoprotein F precursor	6	9	0.842805
IPI00384406	Myosin-reactive immunoglobulin heavy chain variable region	87	81	0.844427
IPI00291262	Clusterin precursor	250	218	0.847309
IPI00430808	Hypothetical protein	4635	4087	0.851983
IPI00555872	Myosin-reactive immunoglobulin heavy chain variable region	303	266	0.863951
IPI00479553	11 kDa protein	302	264	0.864583
IPI00384392	Myosin-reactive immunoglobulin heavy chain variable region	300	262	0.865603
IPI00440577	Hypothetical protein	4470	3950	0.868276
IPI00006515	Splice Isoform 1 of Potassium voltage-gated channel subfamily H member 6	2	5	0.86865
IPI00012792	Vascular endothelial-cadherin precursor	2	5	0.86865
IPI00021885	Splice Isoform Alpha-E of Fibrinogen alpha/alpha-E chain precursor	2	5	0.86865
IPI00160265	Splice Isoform 2 of Trinucleotide repeat-containing gene 6A protein	2	5	0.86865
IPI00550048	Similar to Ig kappa chain V-IV region B17 precursor	3638	3224	0.870499
IPI00550731	Hypothetical protein	4472	3952	0.870537
IPI00019581	Coagulation factor XII precursor	93	86	0.871382
IPI00554675	Anti-RhD monoclonal T125 kappa light chain precursor	4387	3879	0.87278

IPI00477069	10 kDa protein	299	261	0.875015
IPI00513925	Complement factor H-related 1	66	63	0.875888
IPI00004962	130 kD Golgi-localized phosphoprotein	11	16	0.876775
IPI00414037	Myosin tail domain-containing protein	7	11	0.878179
IPI00419424	IGKV1-5 protein	4464	3952	0.886142
IPI00385058	Hypothetical protein	4447	3937	0.886576
IPI00022417	Leucine-rich alpha-2-glycoprotein precursor	113	109	0.888383
IPI00455228	PREDICTED: similar to hect domain and RLD 2	6	10	0.890042
IPI00000775	Splice Isoform 1 of Leucine-rich repeats and immunoglobulin-like domains protein 1 precursor	0	2	0.891244
IPI00000846	Splice Isoform 1 of Chromodomain helicase-DNA-binding protein 4	0	2	0.891244
IPI00000977	Serine/threonine protein kinase	0	2	0.891244
IPI00001342	NY-REN-24 antigen	0	2	0.891244
IPI00001434	Protocadherin beta 14 precursor	0	2	0.891244
IPI00001737	Homeobox protein GSH-2	0	2	0.891244
IPI00001758	Outer dense fiber of sperm tails 2	0	2	0.891244
IPI00002180	PREDICTED: KIAA1383 protein	0	2	0.891244
IPI00002283	PREDICTED: KIAA1337 protein	0	2	0.891244
IPI00002707	Splice Isoform 1 of Spastin	0	2	0.891244
IPI00003148	Splice Isoform Alpha-1 of Mitogen-activated protein kinase 10	0	2	0.891244
IPI00003515	Thyroid receptor interacting protein 11	0	2	0.891244
IPI00004247	Splice Isoform 1 of Phosphorylase b kinase alpha regulatory chain, skeletal muscle	0	2	0.891244

IPI00005030	BOG25	0	2	0.891244
IPI00005036	RNA-binding protein 5	0	2	0.891244
IPI00005126	Ephrin-B2 precursor	0	2	0.891244
IPI00005541	PMS1 protein homolog 1	0	2	0.891244
IPI00005781	Splice Isoform A of Arachidonate 15-lipoxygenase, type II	0	2	0.891244
IPI00006130	Protein KIAA0494	0	2	0.891244
IPI00006994	Kelch-like protein 21	0	2	0.891244
IPI00007960	Splice Isoform 1 of Periostin precursor	0	2	0.891244
IPI00008115	Splice Isoform 1 of Mitogen-activated protein kinase kinase kinase 9	0	2	0.891244
IPI00008269	Hypothetical protein FLJ16174	0	2	0.891244
IPI00008542	F-box only protein 28	0	2	0.891244
IPI00010163	Zinc finger protein 286	0	2	0.891244
IPI00011062	Splice Isoform 1 of Carbamoyl-phosphate synthase [ammonia], mitochondrial precursor	0	2	0.891244
IPI00011063	Neurotrypsin precursor	0	2	0.891244
IPI00011218	Macrophage colony stimulating factor I receptor precursor	0	2	0.891244
IPI00011232	KIAA1536 protein	0	2	0.891244
IPI00011689	Elongation factor 2 kinase	0	2	0.891244
IPI00011757	RNA polymerase II elongation factor ELL2	0	2	0.891244
IPI00011805	Neuralized-like protein 2	0	2	0.891244
IPI00012094	Splice Isoform Long of Probable ubiquitin carboxyl-terminal hydrolase FAF-Y	0	2	0.891244
IPI00012995	Pre-mRNA cleavage factor I 25 kDa subunit	0	2	0.891244
IPI00014572	SPARC precursor	0	2	0.891244
IPI00014878	Protein kinase C, mu type	0	2	0.891244

IPI00016347	Hypothetical protein	0	2	0.891244
IPI00016431	Hypothetical protein FLJ23556	0	2	0.891244
IPI00017382	ETS translocation variant 4	0	2	0.891244
IPI00017730	Mothers against decapentaplegic homolog 5	0	2	0.891244
IPI00018415	Transmembrane 9 superfamily protein member 2 precursor	0	2	0.891244
IPI00020546	Ash1	0	2	0.891244
IPI00020557	Low-density lipoprotein receptor-related protein 1 precursor	0	2	0.891244
IPI00020900	Splice Isoform 1 of Zinc finger protein 41	0	2	0.891244
IPI00020920	Adenylate cyclase, type VII	0	2	0.891244
IPI00021075	Splice Isoform 1 of Large neutral amino acids transporter small subunit 3	0	2	0.891244
IPI00022143	Family with sequence similarity 62 (C2 domain containing), member A	0	2	0.891244
IPI00022462	Transferrin receptor protein 1	0	2	0.891244
IPI00022479	P532	0	2	0.891244
IPI00023549	HSPC159 protein	0	2	0.891244
IPI00023630	Splice Isoform 1 of Apoptotic protease activating factor 1	0	2	0.891244
IPI00024214	Splice Isoform 1 of Telomeric repeat binding factor 2	0	2	0.891244
IPI00024272	Integral membrane protein DGCR2/IDD precursor	0	2	0.891244
IPI00024417	Huntingtin interacting protein 1 related	0	2	0.891244
IPI00024524	Putatative 28 kDa protein	0	2	0.891244
IPI00024700	HERV-H LTR-associating 1	0	2	0.891244

IPI00024714	Splice Isoform 1 of Regulator of G-protein signaling 12	0	2	0.891244
IPI00024934	Methylmalonyl-CoA mutase, mitochondrial precursor	0	2	0.891244
IPI00026183	Small inducible cytokine A18 precursor	0	2	0.891244
IPI00026828	Splice Isoform 1 of Tripartite motif protein 9	0	2	0.891244
IPI00027232	Insulin-like growth factor I receptor	0	2	0.891244
IPI00027251	Serine/threonine-protein kinase 38	0	2	0.891244
IPI00027801	Hypothetical protein DKFZp434O1826	0	2	0.891244
IPI00027875	Synaptotagmin-11	0	2	0.891244
IPI00028484	Splice Isoform 1 of Neuropsin precursor	0	2	0.891244
IPI00031506	Potassium/sodium hyperpolarization-activated cyclic nucleotide-gated channel 1	0	2	0.891244
IPI00031519	Splice Isoform 1 of DNA	0	2	0.891244
IPI00031557	Splice Isoform 1 of Cystathionine gamma-	0	2	0.891244
IPI00032144	LIM/homeobox protein Lhx2	0	2	0.891244
IPI00032333	Splice Isoform 1 of Receptor-type tyrosine-protein phosphatase alpha precursor	0	2	0.891244
IPI00034308	Sarcosine dehydrogenase, mitochondrial precursor	0	2	0.891244
IPI00044749	Splice Isoform 1 of Serine/threonine-protein kinase Nek1	0	2	0.891244
IPI00044751	M-phase phosphoprotein 1	0	2	0.891244
IPI00044891	Adaptor molecule-1	0	2	0.891244
IPI00045856	Hypothetical protein FLJ14717	0	2	0.891244
IPI00058961	49 kDa protein	0	2	0.891244
IPI00064200	MasterMind-like 2	0	2	0.891244
IPI00065057	Hypothetical protein FLJ25444	0	2	0.891244

IPI00072534	SMAP-1b	0	2	0.891244
IPI00098988	Hypothetical protein DKFZp434C196	0	2	0.891244
IPI00102107	Splice Isoform 1 of Nuclear receptor binding SET domain containing protein 1	0	2	0.891244
IPI00102377	Ankyrin repeat domain 27	0	2	0.891244
IPI00152615	Alpha3-fucosyltransferase	0	2	0.891244
IPI00152639	ADAMTS-19 precursor	0	2	0.891244
IPI00154910	Cbl-interacting protein Sts-1 variant	0	2	0.891244
IPI00164610	TBC1 domain family member 1	0	2	0.891244
IPI00165979	OTTHUMP00000040002	0	2	0.891244
IPI00167399	Hypothetical protein FLJ40201	0	2	0.891244
IPI00167645	Hypothetical protein DKFZp761O1618	0	2	0.891244
IPI00167941	Midasin	0	2	0.891244
IPI00168352	Polyserase-2 precursor	0	2	0.891244
IPI00168603	Choline dehydrogenase, mitochondrial	0	2	0.891244
IPI00168708	Hypothetical protein	0	2	0.891244
IPI00169065	Olfactory receptor 4D5	0	2	0.891244
IPI00170770	PHD finger protein 3	0	2	0.891244
IPI00170999	NGEP long variant	0	2	0.891244
IPI00171230	Splice Isoform 2 of RAB6 interacting protein	0	2	0.891244
IPI00171491	Splice Isoform 1 of Potassium voltage-gated channel subfamily H member 5	0	2	0.891244
IPI00173359	Uveal autoantigen	0	2	0.891244
IPI00175182	PREDICTED: similar to hypothetical protein DKFZp727G131	0	2	0.891244
IPI00176782	PREDICTED: KIAA1030 protein	0	2	0.891244
IPI00177938	Splice Isoform 2 of Transducin-like enhancer protein 3	0	2	0.891244

IPI00184061	Hypothetical protein FLJ34266	0	2	0.891244
IPI00185919	KIAA0731 protein	0	2	0.891244
IPI00187146	PREDICTED: hypothetical protein	0	2	0.891244
IPI00216438	Solute carrier family 12 (Sodium/chloride tranSporterS), member 3	0	2	0.891244
IPI00216992	PREDICTED: KIAA2025 protein	0	2	0.891244
IPI00217225	Splice Isoform 1 of Nitric-oxide synthase,	0	2	0.891244
IPI00217504	107 kDa protein	0	2	0.891244
IPI00218784	Splice Isoform 2 of Sphingomyelin phosphodiesterase precursor	0	2	0.891244
IPI00220391	Splice Isoform 1 of Ryanodine receptor 1	0	2	0.891244
IPI00254606	Zinc finger protein 628	0	2	0.891244
IPI00256434	Hypothetical protein FLJ16008	0	2	0.891244
IPI00289107	Hypothetical protein FLJ31675	0	2	0.891244
IPI00289866	Forkhead box protein O1A	0	2	0.891244
IPI00293430	Putative ATP-dependent mitochondrial RNA helicase	0	2	0.891244
IPI00294705	Hypothetical protein	0	2	0.891244
IPI00294959	ZASP protein	0	2	0.891244
IPI00295816	OTTHUMP00000044920	0	2	0.891244
IPI00295900	Hypothetical protein FLJ44660	0	2	0.891244
IPI00297089	A-kinase anchor protein 6	0	2	0.891244
IPI00297940	Hypothetical protein FLJ40073	0	2	0.891244
IPI00298902	PREDICTED: KIAA1107 protein	0	2	0.891244
IPI00300332	Splice Isoform 1 of B-cell lymphoma/leukemia 11A	0	2	0.891244
IPI00300420	14 kDa protein	0	2	0.891244
IPI00301180	Solute carrier family 12, member 5	0	2	0.891244

IPI00301360	Splice Isoform 1 of Tumor protein p73-like	0	2	0.891244
IPI00302742	FLJ11588 protein	0	2	0.891244
IPI00304379	Ubiquitin carboxyl-terminal hydrolase 1	0	2	0.891244
IPI00304999	Tyrosine-protein kinase transmembrane receptor ROR2 precursor	0	2	0.891244
IPI00307230	PREDICTED: similar to bA304I5.1 (novel lipase)	0	2	0.891244
IPI00307535	Hypothetical protein FLJ32736	0	2	0.891244
IPI00328089	Immunodeficiency virus type I enhancer binding protein 3	0	2	0.891244
IPI00328342	Sodium bicarbonate cotransporter 3	0	2	0.891244
IPI00328712	Hypothetical protein MGC44505	0	2	0.891244
IPI00329749	RIN3	0	2	0.891244
IPI00332628	Otopetrin 3	0	2	0.891244
IPI00333434	Osteoclast stimulating factor 1	0	2	0.891244
IPI00334320	48 kDa protein	0	2	0.891244
IPI00334799	Splice Isoform 3 of Ryanodine receptor 1	0	2	0.891244
IPI00335085	SPla/RYanodine receptor SPRY domain containing protein	0	2	0.891244
IPI00337314	Hypothetical protein ALF	0	2	0.891244
IPI00374930	PREDICTED: hypothetical protein	0	2	0.891244
IPI00375294	Laminin alpha-1 chain precursor	0	2	0.891244
IPI00375330	FOE	0	2	0.891244
IPI00377257	Splice Isoform 3 of Phosphatase and actin regulator 3	0	2	0.891244
IPI00382531	HERV-K_5q33.3 provirus ancestral Pol	0	2	0.891244
IPI00384678	Splice Isoform 2 of FYVE, RhoGEF and PH domain containing protein 2	0	2	0.891244

IPI00385480	150 kDa protein	0	2	0.891244
IPI00396063	C-MYC promoter-binding protein IRLB	0	2	0.891244
IPI00396314	Hypothetical protein FLJ13165	0	2	0.891244
IPI00396394	PREDICTED: similar to Munc13-3 protein -	0	2	0.891244
IPI00396522	ZNF614 protein	0	2	0.891244
IPI00397645	Hypothetical protein FLJ46603	0	2	0.891244
IPI00397675	Formin homology 2 domain containing 3	0	2	0.891244
IPI00398554	PREDICTED: hypothetical protein	0	2	0.891244
IPI00398806	H2A histone family, member V isoform 5	0	2	0.891244
IPI00398898	PREDICTED: similar to RIKEN cDNA 6430537H07 gene	0	2	0.891244
IPI00402594	PREDICTED: hypothetical protein	0	2	0.891244
IPI00410571	Protein exPressed in Prostate, ovary, testis, and Placenta 14 isoform POTE-14B	0	2	0.891244
IPI00411343	Hypothetical protein FLJ45651	0	2	0.891244
IPI00413518	Splice Isoform 1 of Mirror-image polydactyly gene 1 protein	0	2	0.891244
IPI00414612	Hexokinase domain containing 1	0	2	0.891244
IPI00418957	Hypothetical protein FLJ44653	0	2	0.891244
IPI00444602	Hypothetical protein FLJ45082	0	2	0.891244
IPI00445315	Family with sequence similarity 47, member	0	2	0.891244
IPI00455219	PREDICTED: AT rich interactive domain 5B (MRF1-like)	0	2	0.891244
IPI00455508	PREDICTED: similar to RIKEN cDNA 2610318N02	0	2	0.891244
IPI00456069	PREDICTED: hypothetical protein	0	2	0.891244
IPI00456336	PREDICTED: hypothetical protein	0	2	0.891244
IPI00456341	PREDICTED: hypothetical protein	0	2	0.891244

IPI00456382	PREDICTED: hypothetical protein	0	2	0.891244
IPI00464950	Novel protein	0	2	0.891244
IPI00465050	LLGL2 protein	0	2	0.891244
IPI00465164	KIAA0194 protein	0	2	0.891244
IPI00465325	QVSK201	0	2	0.891244
IPI00470764	Splice Isoform 6 of BAI1-associated protein	0	2	0.891244
IPI00470771	Hypothetical protein FLJ10211	0	2	0.891244
IPI00477179	Hypothetical protein DKFZp686F21172	0	2	0.891244
IPI00478380	91 kDa protein	0	2	0.891244
IPI00478870	Hypothetical protein	0	2	0.891244
IPI00514002	Hypothetical protein DKFZp781J069	0	2	0.891244
IPI00549745	Hypothetical protein DKFZp781M0415	0	2	0.891244
IPI00556076	Mastermind-like 1 variant	0	2	0.891244
IPI00556430	Syntaxin 16 isoform a variant	0	2	0.891244
IPI00473097	IGKC protein	4447	3940	0.892749
IPI00430848	IGKC protein	4443	3937	0.892848
IPI00448845	Hypothetical protein	4443	3937	0.892848
IPI00549330	Ig kappa chain C region	4443	3937	0.892848
IPI00472961	Hypothetical protein	4451	3944	0.894646
IPI00152653	Ciliary dynein heavy chain 5	13	18	0.896825
IPI00294810	Hypothetical protein FLJ10824	3	7	0.897715
IPI00384399	Myosin-reactive immunoglobulin light chain variable region	3	7	0.897715
IPI00430839	Hypothetical protein	4442	3939	0.898795
IPI00430847	IGKC protein	4462	3958	0.902116
IPI00007119	Tubulin epsilon chain	1	4	0.904268
IPI00023109	Chromodomain-helicase-DNA-binding protein 2	1	4	0.904268

IPI00025178	Breast carcinoma amplified sequence 2	1	4	0.904268
IPI00152269	WD repeat domain 51B	1	4	0.904268
IPI00382483	Ig heavy chain V-III region GA	1	4	0.904268
IPI00022431	Alpha-2-HS-glycoprotein precursor	186	172	0.908531
IPI00026199	Plasma glutathione peroxidase precursor	40	42	0.909852
IPI00025864	Cholinesterase precursor	39	41	0.912875
IPI00441043	Hypothetical protein	4449	3952	0.913242
IPI00025862	C4b-binding protein beta chain precursor	7	12	0.914465
IPI00022395	Complement component C9 precursor	115	112	0.920008
IPI00011107	Isocitrate dehydrogenase [NADP], mitochondrial precursor	2	6	0.921432
IPI00457026	PREDICTED: similar to GP80 precursor	2	6	0.921432
IPI00022937	Coagulation factor V	13	19	0.923211
IPI00550315	Ig kappa chain C region	4211	3750	0.923886
IPI00166137	RALY-like protein isoform 1	9	15	0.924684
IPI00479723	Splice Isoform 10 of Fibronectin precursor	1911	1733	0.935153
IPI00256861	Splice Isoform 2 of Microtubule-actin crosslinking factor 1, isoforms 1/2/3/5	3	8	0.93606
IPI00292218	Hepatocyte growth factor-like protein	3	8	0.93606
IPI00432363	Microtubule-actin crosslinking factor 1, isoform 4	3	8	0.93606
IPI00479928	11 kDa protein	24	29	0.936767
IPI00004656	Beta-2-microglobulin precursor	7	13	0.941067
IPI00383105	Chromodomain heliCase DNA binding	7	13	0.941067
IPI00022418	Splice Isoform 1 of Fibronectin precursor	1923	1752	0.942388
IPI00010295	Carboxypeptidase N catalytic chain precursor	21	27	0.945871
IPI00414283	Fibronectin 1 isoForm 4 preproprotein	1950	1786	0.946411
IPI00009101	Transcriptional activator SRCAP	4	10	0.947817

IPI00296437	Type III iodothyronine deiodinase	4	10	0.947817
IPI00167908	Hypothetical protein FLJ35834	1	5	0.948036
IPI00218130	Glycogen phosphorylase, muscle form	1	5	0.948036
IPI00329251	Hypothetical protein MGC39581	1	5	0.948036
IPI00480107	565 kDa protein	1	5	0.948036
IPI00004358	Glycogen phosphorylase, brain form	0	3	0.949289
IPI00005689	KIAA1124 protein	0	3	0.949289
IPI00007362	Ubiquitously transcribed X chromosome tetratricopeptide repeat protein	0	3	0.949289
IPI00007929	FLJ20297 protein	0	3	0.949289
IPI00017386	Hypothetical protein FLJ12704	0	3	0.949289
IPI00018411	PREDICTED: zinc finger protein 629	0	3	0.949289
IPI00027310	Multiple EGF-like-domain protein 4	0	3	0.949289
IPI00027843	Splice Isoform 1 of Vitamin K-dependent protein Z precursor	0	3	0.949289
IPI00027848	Macrophage mannose receptor precursor	0	3	0.949289
IPI00168319	Hypothetical protein FLJ90511	0	3	0.949289
IPI00180408	PREDICTED: KIAA1000 protein	0	3	0.949289
IPI00183938	Hypothetical protein FLJ20272	0	3	0.949289
IPI00251559	Ring finger protein 20	0	3	0.949289
IPI00294728	X-like 1 protein	0	3	0.949289
IPI00298235	Splice Isoform 1 of Protein O-mannosyl-transferase 1	0	3	0.949289
IPI00301143	HGSC289	0	3	0.949289
IPI00301480	1-phosphatidylinositol-4,5-bisphosphate phosphodiesterase beta 2	0	3	0.949289
IPI00304866	Tumor necrosis factor, alpha-induced protein	0	3	0.949289
IPI00328211	Novel protein	0	3	0.949289

IPI00329038	Splice Isoform 1 of CDK5 regulatory subunit associated protein 2	0	3	0.949289
IPI00412869	Similar to Kendrin	0	3	0.949289
IPI00419263	Peroxisomal 3,2-trans-enoyl-CoA isomerase	0	3	0.949289
IPI00465231	Tolloid-like 2 protein	0	3	0.949289
IPI00477896	Anchor protein	0	3	0.949289
IPI00179357	Titin	6	12	0.949513
IPI00375498	TiTin isoform novex-1	6	12	0.949513
IPI00017696	Complement C1s subcomponent precursor	73	76	0.951274
IPI00041065	HGF activator like protein	38	43	0.954146
IPI00009028	Tetranectin precursor	70	73	0.954708
IPI00029168	Apolipoprotein(a) precursor	68	71	0.957019
IPI00455173	Titin, heart isoform N2-B	5	11	0.957933
IPI00011264	Complement factor H-related protein 1 precursor	114	116	0.958167
IPI00296176	Coagulation factor IX precursor	13	21	0.958288
IPI00029863	Alpha-2-antiplasmin precursor	174	168	0.95958
IPI00022432	Transthyretin precursor	1724	1572	0.960046
IPI00019439	Fibrillin 2 precursor	7	14	0.960086
IPI00032220	Angiotensinogen precursor	148	146	0.961239
IPI00382577	Kappa 1 light chain variable region	20	27	0.961842
IPI00303963	Complement C2 precursor	44	50	0.962162
IPI00470919	Hypothetical protein DKFZp686K08164	1862	1703	0.966769
IPI00384402	Myosin-reactive immunoglobulin kappa chain variable region	8	16	0.968507
IPI00387116	Ig kappa chain V-III region NG9 precursor	8	16	0.968507
IPI00298828	Beta-2-glycoprotein I precursor	297	279	0.970555
IPI00217444	Splice Isoform 1 of Tripartite motif protein	1	6	0.972262

IPI00396231	Glycosyltransferase	1	6	0.972262
IPI00429536	HERV-K_22q11.23 provirus ancestral Gag polyprotein	1	6	0.972262
IPI00014845	OTTHUMP00000039711	7	15	0.973385
IPI00377208	Splice Isoform 1 Synomyms=a of Cadherin-like protein 26 precursor	2	8	0.973579
IPI00398231	PREDICTED: similar to Ig lambda light chain leader and V-region	2	8	0.973579
IPI00166930	62 kDa protein	92	93	0.973596
IPI00045510	IAP-like protein 3	12	21	0.97387
IPI00163207	Splice Isoform 1 of N-acetylmuramoyl-L-alanine amidase precursor	157	155	0.973997
IPI00020996	Insulin-like growth factor binding protein complex acid labile chain precursor	73	79	0.97521
IPI00382436	Ig lambda chain V-III region SH	35	42	0.975214
IPI00016334	Cell surface glycoprotein MUC18 precursor	0	4	0.976357
IPI00018136	Splice Isoform 1 of Vascular cell adhesion protein 1 precursor	0	4	0.976357
IPI00021891	Splice Isoform Gamma-B of Fibrinogen gamma chain precursor	0	4	0.976357
IPI00166161	Hypothetical protein C14orf39	0	4	0.976357
IPI00242630	Hypothetical protein FLJ20397	0	4	0.976357
IPI00382395	Hypothetical protein FLJ31569	0	4	0.976357
IPI00470657	Anti-colorectal carcinoma heavy chain	0	4	0.976357
IPI00025204	CD5 antigen-like precursor	65	72	0.976714
IPI00178926	ImmunoglobulIn J chaIn	13	23	0.978516
IPI00165421	SERPINC1 protein	224	219	0.982537
IPI00552385	V2-17 protein	28	37	0.984023

IPI00383111	Keratin 10	879	781	0.984423
IPI00027482	Corticosteroid-binding globulin precursor	27	36	0.985362
IPI00410606	Splice Isoform 1 of Transient receptor potential cation channel subfamily V member	1	7	0.985381
IPI00021439	Actin, cytoplasmic 1	6	15	0.986199
IPI00464978	Insulin receptor substrate 2 insertion mutant	6	15	0.986199
IPI00032291	Complement C5 precursor	442	421	0.987074
IPI00387120	Ig kappa chain V-IV region Len	88	93	0.988208
IPI00386133	Ig kappa chain V-IV region B17 precursor	47	57	0.988702
IPI00024853	Splice Isoform 1 of Periaxin	0	5	0.988979
IPI00166933	Chromosome 10 open reading frame 12	0	5	0.988979
IPI00398625	Hornerin	0	5	0.988979
IPI00184598	37 kDa protein	59	70	0.989875
IPI00398874	Similar to RIKEN cDNA 9230116B18 gene	12	24	0.99106
IPI00159652	PREDICTED: KIAA0826 protein	6	16	0.991291
IPI00299503	Phosphatidylinositol-glycan-specific phospholipase D 1 precursor	68	80	0.992149
IPI00005826	HERC2 protein	1	8	0.992372
IPI00552852	V2-19 protein	4	13	0.992476
IPI00555746	Paraoxonase 1	174	181	0.99314
IPI00556287	Hypothetical protein	1575	1421	0.993546
IPI00376383	CENTRIOLIN	0	6	0.994863
IPI00387109	Ig kappa chain V-II region FR	0	6	0.994863
IPI00022426	AMBP protein precursor	210	217	0.995158
IPI00218732	Serum paraoxonase/arylesterase 1	138	149	0.99561
IPI00020986	Lumican precursor	110	124	0.995877
IPI00166729	Alpha-2-glycoprotein 1, zinc	109	123	0.996028
IPI00009865	Keratin, type I cytoskeletal 10	1042	958	0.997029

IPI00375600	Beta 3-glycosyltransferase-like	0	7	0.997606
IPI00009920	Complement component C6 precursor	120	137	0.99773
IPI00291866	Plasma protease C1 inhibitor precursor	411	410	0.998044
IPI00300341	Transcription elongation factor B polypeptide	127	143	0.998078
IPI00298853	Vitamin D-binding protein precursor	903	830	0.998331
IPI00470901	DeDicator of cytokinesis 5	4	16	0.99851
IPI00006114	Pigment epithelium-derived factor precursor	133	150	0.998659
IPI00013495	Splice Isoform 2 of ATP-binding cassette, sub-family F, member 1	24	40	0.998924
IPI00296170	Haptoglobin-related protein	2007	1960	0.998954
IPI00334362	C114 SLIT-like testicular protein	1	11	0.99896
IPI00555812	Vitamin D-binding protein variant	914	846	0.998967
IPI00019568	Prothrombin precursor	537	535	0.999319
IPI00470478	Chromosome 17 open reading frame 27	14	31	0.999325
IPI00477597	Haptoglobin-related protein precursor	2145	2084	0.99947
IPI00151141	Splice Isoform 1 of Serine/threonine-protein kinase WNK4	0	9	0.99948
IPI00551005	Ig lambda chain C regions	7054	6437	0.999513
IPI00387025	Ig kappa chain V-I region DEE	3	16	0.999516
IPI00550061	Full-length cDNA clone CS0DI019YF20 of Placenta of Homo sapiens	3099	2921	0.999729
IPI00549576	Ig gamma-1 chain C region	8699	7917	0.99976
IPI00305461	Inter-alpha-trypsin inhibitor heavy chain H2 precursor	1113	1060	0.999849
IPI00027235	Splice Isoform 1 of Attractin precursor	84	112	0.999939
IPI00472345	IGHG3 protein	2919	2801	0.999973
IPI00165972	Adipsin/complement factor D precursor	9	32	0.999976
IPI00554766	Hypothetical protein	2912	2799	0.99998

IPI00023019	Splice Isoform 1 of Sex hormone-binding globulin precursor	5	27	0.999991
IPI00026314	Gelsolin precursor	399	439	0.999992
IPI00022463	Serotransferrin precursor	6993	6502	0.999994
IPI00296537	Splice Isoform C of Fibulin-1 precursor	48	84	0.999997
IPI00382606	Factor VII active site mutant	9405	8775	1
IPI00296534	Splice Isoform D of Fibulin-1 precursor	45	95	1
IPI00383732	VH3 protein	365	445	1
IPI00022371	Histidine-rich glycoprotein precursor	210	280	1
IPI00472610	IGHM protein	9856	9251	1
IPI00448984	Hypothetical protein	9804	9208	1
IPI00000357	Activity-regulated cytoskeleton-associated protein	4	41	1
IPI00218192	Splice Isoform 2 of Inter-alpha-trypsin inhibitor heavy chain H4 precursor	540	634	1
IPI00294193	Splice Isoform 1 of Inter-alpha-trypsin inhibitor heavy chain H4 precursor	540	635	1
IPI00549769	Ig gamma-1 chain C region	10159	9642	1
IPI00448925	Hypothetical protein	10195	9675	1
IPI00549440	Ig gamma-1 chain C region	8685	8311	1
IPI00423445	Hypothetical protein DKFZp686P15220	10097	9594	1
IPI00550438	Ig gamma-1 chain C region	10079	9592	1
IPI00550647	Ig gamma-1 chain C region	10021	9540	1
IPI00550945	Ig gamma-1 chain C region	10045	9572	1
IPI00550509	Ig gamma-1 chain C region	10022	9551	1
IPI00430840	Hypothetical protein	10036	9565	1
IPI00550462	Ig gamma-1 chain C region	10026	9556	1
IPI00550142	Ig gamma-1 chain C region	10023	9554	1

IPI00554615	Anti-RhD monoclonal T125 gamma1 heavy chain precursor	10020	9551	1
IPI00550367	Hypothetical protein DKFZp686I15196	10012	9572	1
IPI00439447	Hypothetical protein	10011	9571	1
IPI00549304	Ig gamma-1 chain C region	8873	8538	1
IPI00384938	Hypothetical protein DKFZp686N02209	10010	9572	1
IPI00426007	Hypothetical protein DKFZp686G11190	10079	9641	1
IPI00423463	Hypothetical protein DKFZp686O01196	10056	9622	1
IPI00304273	Apolipoprotein A-IV precursor	336	560	1
IPI00423464	Hypothetical protein DKFZp686K03196	8134	8065	1
IPI00478761	45 kDa protein	359	542	1
IPI00549462	Ig gamma-1 chain C region	8902	8663	1
IPI00550718	Ig gamma-1 chain C region	8147	8050	1
