# Supplementary material for: Localized-Statistical Quantification of Human Serum Proteome Associated with Type 2 Diabetes
Source: PLoS One. 2008 Sep 16;3(9):e3224. doi: 10.1371/journal.pone.0003224 (PMC2529402; doi:10.1371/journal.pone.0003224)
Supplement: Table S3 — Pathway analysis by mapping 1377 proteins into KEGG pathways. Ratio (%): (100 Ã? Gene number found in pathway) / Totallygene number in pathway. P value: present overall bias of pathways associated with diabetic-serum or non-diabetic serum (0.08 MB PDF) [file pone.0003224.s008.pdf]

Supplementary Table S3

Pathway analysis by mapping 1377 proteins into KEGG pathways. Ratio (%): (100 × Gene number found in pathway) / Totally gene number in pathway. P value: present overall bias of pathways associated with diabetic-serum or non-diabetic serum.

Pathway name	Gene number found in pathway	Totally gene number in pathway	Ratio (%)	Protein number found in pathway	P value
Notch signaling pathway	3	46	6.521739	3	0.83108608
Tryptophan metabolism	4	80	5	4	0.55440482
Citrate cycle (TCA cycle)	1	27	3.703704	1	0.92143184
Dorso-ventral axis formation	1	28	3.571429	1	0.66528913
Glycolysis / Gluconeogenesis	6	61	9.836066	6	0.26835121
Phenylalanine metabolism	2	28	7.142857	2	0.06058192
Wnt signaling pathway	8	145	5.517241	8	0.81577452
gamma-Hexachlorocyclohexane degradation	2	24	8.333333	2	0.99270136
Bisphenol A degradation	2	13	15.38462	2	0.99270136
PPAR signaling pathway	10	67	14.92537	10	2.60E-08
Tyrosine metabolism	1	56	1.785714	1	0.25109763
Histidine metabolism	4	40	10	4	0.18227911
Chronic myeloid leukemia	1	75	1.333333	1	0.37963333
Acute myeloid leukemia	1	51	1.960784	1	0.37963333
Small cell lung cancer	9	86	10.46512	12	0.98974253
Arginine and proline metabolism	5	33	15.15152	5	0.74419946
Non-small cell lung cancer	2	53	3.773585	2	0.24472455
Valine, leucine and isoleucine	3	44	6.818182	3	0.62723768
Colorectal cancer	6	85	7.058824	6	0.40297619

Renal cell carcinoma	3	68	4.411765	3	0.93215142
Pancreatic cancer	2	73	2.739726	2	0.74386084
Endometrial cancer	2	51	3.921569	2	0.14123114
Glioma	2	61	3.278689	2	0.74386084
Methionine metabolism	3	18	16.66667	3	0.85001952
Prostate cancer	4	86	4.651163	4	0.85991733
Cysteine metabolism	2	17	11.76471	2	0.80839113
Thyroid cancer	1	28	3.571429	1	0.25109763
Basal cell carcinoma	2	56	3.571429	2	0.09132949
Melanoma	2	69	2.898551	2	0.74386084
Bladder cancer	2	39	5.128205	2	0.64401805
Lysine degradation	4	44	9.090909	4	0.77439014
Glycine, serine and threonine metabolism	4	43	9.302326	4	0.93501689
Apoptosis	6	84	7.142857	6	0.22443716
Lysine biosynthesis	1	3	33.33333	1	0.25109763
Glutamate metabolism	2	29	6.896552	2	0.52559162
Alanine and aspartate metabolism	1	32	3.125	1	0.12664644
Arachidonic acid metabolism	5	53	9.433962	5	0.98319126
Gap junction	3	93	3.225806	3	0.95130776
Linoleic acid metabolism	2	34	5.882353	2	0.74791995
Pyrimidine metabolism	2	87	2.298851	2	0.09987102
Tight junction	10	115	8.695652	10	0.9925035
Purine metabolism	3	142	2.112676	3	0.84457444
Adherens junction	7	73	9.589041	7	0.84327614
Urea cycle and metabolism of amino	4	30	13.33333	4	0.47828646
Focal adhesion	27	191	14.13613	34	0.99897507
Glycerolipid metabolism	2	56	3.571429	2	0.366048

Inositol phosphate metabolism	5	49	10.20408	5	0.41468817
ECM-receptor interaction	19	85	22.35294	23	0.7861081
Glycosylphosphatidylinositol(GPI)-anchor biosynthesis	1	23	4.347826	1	0.99214866
Glycerophospholipid metabolism	2	64	3.125	2	0.62764397
Cell adhesion molecules (CAMs)	7	122	5.737705	7	0.77963251
Ether lipid metabolism	1	29	3.448276	1	0.82757761
Peptidoglycan biosynthesis	1	2	50	1	0.97399713
Glycosaminoglycan degradation	2	15	13.33333	2	0.366048
Heparan sulfate biosynthesis	1	18	5.555556	1	0.12664644
Neurodegenerative Disorders	4	38	10.52632	4	0.00213209
Streptomycin biosynthesis	1	10	10	1	0.10218227
Glutathione metabolism	3	37	8.108108	3	0.90504689
N-Glycan biosynthesis	2	36	5.555556	2	0.7489258
N-Glycan degradation	1	15	6.666667	1	0.5
Starch and sucrose metabolism	8	81	9.876543	8	0.76818175
Selenoamino acid metabolism	2	29	6.896552	2	0.6544955
Folate biosynthesis	5	39	12.82051	5	0.30271349
Taste transduction	1	53	1.886792	1	0.94928867
Cell Communication	43	130	33.07692	48	2.89E-05
Biotin metabolism	1	4	25	1	0.63849762
Long-term depression	8	74	10.81081	9	0.99957142
Long-term potentiation	5	66	7.575758	5	0.6970797
Leukocyte transendothelial migration	7	106	6.603774	7	0.98883247
Circadian rhythm	1	12	8.333333	1	0.25109763
T cell receptor signaling pathway	4	91	4.395604	4	0.03705357
B cell receptor signaling pathway	3	62	4.83871	3	0.08035597
Fc epsilon RI signaling pathway	5	72	6.944444	5	0.62779054

beta-Alanine metabolism	2	25	8	2	0.5
Natural killer cell mediated cytotoxicity	5	124	4.032258	5	0.04805757
Hematopoietic cell lineage	9	80	11.25	9	0.23274536
Methane metabolism	1	9	11.11111	1	0.06414751
Jak-STAT signaling pathway	5	148	3.378378	5	0.44010543
Reductive carboxylate cycle (CO2	1	10	10	1	0.92143184
Toll-like receptor signaling pathway	8	93	8.602151	8	0.1209601
Carbon fixation	1	20	5	1	0.66528913
Antigen processing and presentation	2	68	2.941176	2	0.929892
Renin-angiotensin system	2	17	11.76471	2	0.8175003
Butanoate metabolism	1	43	2.325581	1	0.5
Propanoate metabolism	3	34	8.823529	3	0.76175864
mTOR signaling pathway	1	47	2.12766	1	0.37963333
Type I diabetes mellitus	2	37	5.405405	2	0.33048645
Benzoate degradation via CoA ligation	1	26	3.846154	1	0.82757761
Pathogenic Escherichia coli infection - EHEC	3	51	5.882353	3	0.66249115
Pathogenic Escherichia coli infection - EPEC	3	51	5.882353	3	0.66249115
Oxidative phosphorylation	2	126	1.587302	2	0.0402752
Metabolism of xenobiotics by cytochrome P450	1	67	1.492537	1	0.5
Type II diabetes mellitus	5	43	11.62791	5	0.54779228
Pyruvate metabolism	2	41	4.878049	2	0.5
Epithelial cell signaling in Helicobacter pylori infection	2	68	2.941176	2	0.34420202
SNARE interactions in vesicular [illegible]	2	36	5.555556	2	0.52559162

Neuroactive ligand-receptor interaction	8	248	3.225806	8	0.92311608
Aminoacyl-tRNA biosynthesis	3	38	7.894737	3	0.10284719
Adipocytokine signaling pathway	8	71	11.26761	8	0.30336797
Cholera - Infection	1	40	2.5	1	0.0359066
Prion disease	1	12	8.333333	1	0.12664644
Ubiquitin mediated proteolysis	3	43	6.976744	3	0.99992212
Phosphatidylinositol signaling system	7	74	9.459459	7	0.35751101
Insulin signaling pathway	13	129	10.07752	13	0.99829598
GnRH signaling pathway	5	93	5.376344	5	0.98802619
Sphingolipid metabolism	2	37	5.405405	2	0.80839113
Melanogenesis	5	99	5.050505	5	0.83742408
Glycosphingolipid biosynthesis - neo-lactoseries	1	20	5	1	0.31414323
Glycosphingolipid biosynthesis - globoseries	1	14	7.142857	1	0.31414323
Dentatorubropallidoluysian atrophy (DRPLA)	1	12	8.333333	1	0.89124382
Glycosphingolipid biosynthesis - ganglioseries	1	15	6.666667	1	0.31414323
Cell cycle	2	113	1.769912	2	0.74791995
Cytokine-cytokine receptor interaction	7	245	2.857143	7	0.02268432
p53 signaling pathway	5	68	7.352941	5	0.70935122
Huntington's disease	2	29	6.896552	2	0.74791995
Androgen and estrogen metabolism	1	52	1.923077	1	0.5
Phenylpropanoid biosynthesis	1	7	14.28571	1	0.06414751
Amyotrophic lateral sclerosis (ALS)	1	18	5.555556	1	0.25109763
Ubiquinone biosynthesis	1	15	6.666667	1	0.25109763

Alzheimer's disease	6	27	22.22222	6	4.33E-15
Bile acid biosynthesis	1	37	2.702703	1	0.5
Calcium signaling pathway	13	171	7.602339	17	0.99677283
Fatty acid metabolism	6	50	12	6	0.70097102
Nitrogen metabolism	4	22	18.18182	4	0.62386575
DNA polymerase	2	25	8	2	0.5
Regulation of actin cytoskeleton	20	204	9.803922	26	0.9917139
Porphyrin and chlorophyll metabolism	1	40	2.5	1	0.00147661
VEGF signaling pathway	4	69	5.797101	4	0.22891054
MAPK signaling pathway	12	253	4.743083	12	0.27468745
ErbB signaling pathway	2	86	2.325581	2	0.74386084
Monoterpenoid biosynthesis	1	2	50	1	0.5
Basal transcription factors	1	11	9.090909	1	0.69921902
Limonene and pinene degradation	2	26	7.692308	2	0.5
Axon guidance	7	123	5.691057	7	0.14050907
Fructose and mannose metabolism	2	39	5.128205	2	0.61863335
Galactose metabolism	2	29	6.896552	2	0.18473402
Ascorbate and aldarate metabolism	1	9	11.11111	1	0.5
TGF-beta signaling pathway	10	88	11.36364	10	0.82531451
Glycan structures - biosynthesis 1	1	107	0.934579	1	0.66528913
Glycan structures - biosynthesis 2	1	60	1.666667	1	0.31414323
Glycan structures - degradation	3	27	11.11111	3	0.38992351
Retinol metabolism	1	4	25	1	0.5
ABC transporters - General	2	44	4.545455	2	0.29474626
Hedgehog signaling pathway	1	55	1.818182	1	0.82757761
Pentose phosphate pathway	3	24	12.5	3	0.31338205
Complement system	23	32		25	1.45E-28
coagulation cascades	23	35		25	0.99141195

Complement and coagulation cascades	46	67	68.65672	50	1.27E-08
