# Supplementary material for: Localized-Statistical Quantification of Human Serum Proteome Associated with Type 2 Diabetes
Source: PLoS One. 2008 Sep 16;3(9):e3224. doi: 10.1371/journal.pone.0003224 (PMC2529402; doi:10.1371/journal.pone.0003224)
Supplement: Table S4 — General and clinical parameters of non-diabetic subjects and type 2 diabetic patients (0.05 MB PDF) [file pone.0003224.s009.pdf]

Supplementary Table S4

General and clinical parameters of non-diabetic subjects and type 2 diabetic patients. Sex: 1 = female, 2 = male, WT: weight (kg), FPG: fasting plasma glucose (mmol/L), PG2H: 2-hour plasma glucose (mmol/L), BMI: Body Mass Index, HOMA: The Homeostasis Model Assessment

Diabetic patients	SEX	AGE	WT	FPG	PG2H	BMI	HOMA	HbA1c	C-Peptide	With diabetes
1	2	53	88	10.82	19.08	28.09	2.48	13	2.38	No
2	2	57	60	14.86	7.13	20.28	3.24	8.9	0.37	Yes
3	1	61	58	6.35	13.1	23.83	3.43	6.1	0.76	No
4	1	38	65	23.7	20.1	26.37	13.88	10.3	1.77	Yes
5	1	50	66	7.21	7.78	26.44	2.58	6.5	3.01	Yes
6	2	42	68	9.9	19.99	23.26	3.37	10.4	1.41	No
7	1	43	65	7.46	10.34	25.08	2.82	8.5	1.26	No
8	2	36	58	12.24	17.74	20.07	5.52	11.5	2.03	Yes
9	1	51	70	10.33	11.59	28.4	4.87	8	1.86	Yes
10	1	54	48	8.1	9.94	22.21	1.86	11	0.7	No
11	2	62	64.5	8.87	14.47	23.13	2.09	9	2.49	Yes
12	2	59	54	7.49	20.49	18.91	0.87	12.8	0.5	Yes
13	1	60	65	11.2	13.6	23.88	6.23	8.7	1.4	No
14	2	44	82.5	11.8	12.22	26.04	2.64	10.3	1.49	No
15	1	66	62.5	11.75	14.54	29.32	6.1	9	2.61	Yes
16	2	45	79	12.94	22.84	23.08	2.2	11.7	2	No
17	2	55	65	13.77	14.74	23.03	5.9	10.4	1.2	Yes
18	2	53	64	5.67	17.81	22.15	1.7	11.1	0.2	Yes
19	1	54	66	8.17	15.49	27.12	4.66	12.9	0.4	Yes
20	2	44	80	8.08	12.05	26.12	4.79	7.1	1.41	No
21	1	56	60	7.29	8.76	24.34	3.64	6.3	1.13	Yes

22	2	60	63	7.3	9.9	21.05	3.52	6.6	2.3	Yes
23	1	59	70	6.12	13.4	29.14	4.44	5.6	3.09	No
24	2	42	65	5.98	11.2	22.49	2.51	6.5	2.26	Yes
Non-diabetic subjects	SEX	AGE	WT	FPG	PG2H	BMI	HOMA	HbA1c	C-Peptide	
1	2	38	62	4.5	3.5	21.71	1.68	5.1	0.32	N.A
2	2	43	67	4.9	5.9	21.15	2.29	5.1	1.03	N.A
3	1	50	56	5.06	5.39	20.57	3.06	5.3	1.44	N.A
4	2	50	70	5.05	5.34	23.39	3.82	5	1.05	N.A
5	1	47	65	5.42	5.86	24.17	3.67	5.5	2.12	N.A
6	1	32	60	5	5.6	22.86	2.71	5.2	1.09	N.A
7	2	57	75	5.24	7.41	24.49	4.46	6	2.84	N.A
8	1	44	53	5.37	6.8	21.23	4.32	5.2	1.86	N.A
9	2	42	65	4.4	5.85	21.97	2.14	5.2	1.41	N.A
10	1	36	64	4.1	4.3	23.8	1.38	5.3	0.54	N.A
11	1	32	60	5.18	6.42	22.86	2.57	5.3	1.1	N.A
12	1	47	50	5.18	7.2	19.53	2.78	5.3	1.12	N.A
13	1	42	55	5	7.23	22.31	2.55	5.2	1.42	N.A
14	1	49	55	5.55	5.15	24.44	3.6	5	2.48	N.A
15	1	53	50	5.55	6.93	23.78	3.22	5.1	0.85	N.A
16	1	50	75	4.42	4.43	27.89	3.14	5.8	2.98	N.A
17	2	55	75	5.05	5.76	25.95	11.16	5	0.98	N.A
18	2	33	86	4.63	5.71	29.07	2.3	5.2	1.3	N.A
19	2	46	61	4.87	3.94	21.61	2.58	5.2	1.02	N.A
20	2	42	75	4.97	5.69	23.67	6.77	5.2	2.59	N.A
21	2	40	70	5	5.06	24.22	5.28	5	2.21	N.A
22	1	42	58	5	5.7	22.66	1.26	5	0.34	N.A
23	2	49	75	5.1	5.6	23.41	1.41	5.4	0.25	N.A

24	1	38	55	5.4	5.3	21.48	1.22	4.8	0.54	N.A
